# Clinical practice recommendations on the management of perioperative cardiac arrest: A report from the PERIOPCA Consortium

**DOI:** 10.1186/s13054-021-03695-2

**Published:** 2021-07-29

**Authors:** Athanasios Chalkias, Nicolas Mongardon, Vladimir Boboshko, Vladimir Cerny, Anne-Laure Constant, Quentin De Roux, Gabriele Finco, Francesca Fumagalli, Eleana Gkamprela, Stéphane Legriel, Vladimir Lomivorotov, Aurora Magliocca, Panagiotis Makaronis, Ioannis Mamais, Iliana Mani, Theodoros Mavridis, Paolo Mura, Giuseppe Ristagno, Salvatore Sardo, Nikolaos Papagiannakis, Theodoros Xanthos, for the PERIOPCA Consortium, Athanasios Chalkias, Nicolas Mongardon, Vladimir Boboshko, Vladimir Cerny, Anne-Laure Constant, Quentin De Roux, Gabriele Finco, Francesca Fumagalli, Eleana Gkamprela, Stéphane Legriel, Vladimir Lomivorotov, Aurora Magliocca, Panagiotis Makaronis, Ioannis Mamais, Iliana Mani, Theodoros Mavridis, Paolo Mura, Giuseppe Ristagno, Salvatore Sardo, Nikolaos Papagiannakis, Theodoros Xanthos

**Affiliations:** 1 Department of Anesthesiology, Faculty of Medicine, University of Thessaly, Larisa, Greece; 2 Outcomes Research Consortium, Cleveland, OH 44195 USA; 3 Hellenic Society of Cardiopulmonary Resuscitation, Athens, Greece; 4 Service D’anesthésie-Réanimation Chirurgicale, DMU CARE, DHU A-TVB, Assistance Publique-Hôpitaux de Paris (AP-HP), Hôpitaux Universitaires Henri Mondor, Univ Paris Est Créteil, Faculté de Santé, 94010 Créteil, France; 5 U955-IMRB, Equipe 03 “Pharmacologie Et Technologies Pour Les Maladies Cardiovasculaires (PROTECT)”, Inserm, Univ Paris Est Créteil (UPEC), Ecole Nationale Vétérinaire d’Alfort (EnVA), 94700 Maisons-Alfort, France; 6 Department of Anesthesiology and Intensive Care, E. Meshalkin National Medical Research Center, Novosibirsk, Russian Federation; 7 Department of Anesthesiology, Perioperative Medicine and Intensive Care, Masaryk Hospital, J.E. Purkinje University, Usti Nad Labem, Czech Republic; 8 Center for Research and Development, University Hospital, Hradec Kralove, Czech Republic; 9 Service D’Anesthésie Et Réanimation, Hôpital Européen Georges Pompidou, Assistance Publique-Hôpitaux de Paris, 75015 Paris, France; 10 Department of Medical Sciences and Public Health, University of Cagliari, Cagliari, Italy; 11 Laboratory of Cardiopulmonary Pathophysiology, Department of Cardiovascular Medicine, Istituto di Ricerche Farmacologiche Mario Negri IRCCS, Milan, Italy; 12 National and Kapodistrian University of Athens, Medical School, Postgraduate Study Course (MSc) “Resuscitation”, Athens, Greece; 13 Medical-Surgical Intensive Care Unit, Centre Hospitalier de Versailles - Site André Mignot, 177 Rue de Versailles, Le Chesnay, France; 14 University Paris-Saclay, UVSQ, INSERM, CESP, Team «PsyDev», Villejuif, France; 15 AfterROSC, Paris, France; 16 Novosibirsk State University, Novosibirsk, Russian Federation; 17 Department of Health Sciences, European University Cyprus, Nicosia, Cyprus; 18 1st Department of Neurology, Medical School, National and Kapodistrian University of Athens, Athens, Greece; 19 Department of Anesthesiology, Intensive Care and Emergency, Fondazione IRCCS Ca’ Granda Ospedale Maggiore Policlinico, Milan, Italy; 20 Department of Pathophysiology and Transplantation, University of Milan, Milan, Italy; 21 School of Medicine, European University Cyprus, Nicosia, Cyprus; 22 Department of Anesthesiology, University Hospital of Larisa, Biopolis, Mezourlo, 41110 Larisa, Greece

**Keywords:** Perioperative, Cardiac arrest, Resuscitation, PERIOPCA

## Abstract

**Background:**

Perioperative cardiac arrest is a rare complication with an incidence of around 1 in 1400 cases, but it carries a high burden of mortality reaching up to 70% at 30 days. Despite its specificities, guidelines for treatment of perioperative cardiac arrest are lacking. Gathering the available literature may improve quality of care and outcome of patients.

**Methods:**

The PERIOPCA Task Force identified major clinical questions about the management of perioperative cardiac arrest and framed them into the therapy population [P], intervention [I], comparator [C], and outcome [O] (PICO) format. Systematic searches of PubMed, Embase, and the Cochrane Library for articles published until September 2020 were performed. Consensus-based treatment recommendations were created using the Grading of Recommendations, Assessment, Development, and Evaluation (GRADE) system. The strength of consensus among the Task Force members about the recommendations was assessed through a modified Delphi consensus process.

**Results:**

Twenty-two PICO questions were addressed, and the recommendations were validated in two Delphi rounds. A summary of evidence for each outcome is reported and accompanied by an overall assessment of the evidence to guide healthcare providers.

**Conclusions:**

The main limitations of our work lie in the scarcity of good quality evidence on this topic. Still, these recommendations provide a basis for decision making, as well as a guide for future research on perioperative cardiac arrest.

**Supplementary Information:**

The online version contains supplementary material available at 10.1186/s13054-021-03695-2.

## Introduction

Perioperative cardiac arrest (PERIOPCA) is a rare but potentially catastrophic event [1, 2]. According to the European Resuscitation Council (ERC), the risk of intraoperative and early postoperative PERIOPCA ranges from 4.3 to 34.6 per 10,000 procedures [3]. Other data suggests that the incidence of PERIOPCA in the surgical population is approximately 1 in 1400 cases [4], while the 30-day mortality may reach up to 70% [5].

PERIOPCA is commonly related to “secondary causes”, but many of them are anesthesia-caused or related, such as hypoxia due to failed airway and ventilation management, loss of the airway during transfer, residual neuromuscular block or severe hemodynamic derangement caused by negative inotropic and vasodilator effects of general anesthesia drugs, severe bradycardia after subarachnoid block, immune-mediated, such as anaphylaxis, and nonimmune-mediated drugs adverse effects, e.g. local anesthetic systemic toxicity (LAST), or iatrogenic pneumothorax due to central line insertion [4]. Another significant source of causes or precipitating factors is the surgical intervention itself that may cause significant bleeding, reduce venous return with visceral manipulation or cavity insufflation, and induce deleterious cardiac reflexes [4].

Although PERIOPCA is usually witnessed, with onset under monitoring in face of healthcare professionals and immediate availability of therapeutic means, aggressive cardiopulmonary resuscitation (CPR) does not always lead to return of spontaneous circulation (ROSC) and optimal long-term outcomes, with 55% of the survivors having poor neurological status [6, 7]. The precipitating causes of PERIOPCA are usually known, but these patients constitute an heterogeneous group and their management often requires modification of traditional CPR algorithms [8]. The heterogeneous nature of PERIOPCA is further demonstrated by the differences in survival between intraoperative and postoperative CPR [8]. Indeed, coagulation and hemostasis disturbances may be a perceived as a potential barrier to controlled hypothermia.

To date the ERC published relevant guidelines for this type of cardiac arrest, but their PICOs were fewer and their treatment recommendations were mainly extrapolated from other settings. Guidelines for treatment of PERIOPCA are lacking and are acknowledged as a gap of knowledge [3]. The purpose of this Clinical Practice Recommendations is to support appropriate decision making for patients with PERIOPCA.

## Methods

An international group of experts in the fields of Anesthesiology, Perioperative Medicine, Critical Care, and Resuscitation was invited by the Hellenic Society of Cardiopulmonary Resuscitation to review and evaluate the evidence on management of PERIOPCA. These experts (Task Force) were selected based on clinical experience and leadership in perioperative patient management; involvement in research, education, and training in PERIOPCA and resuscitation; and familiarity with resuscitation guidelines. The Task Force communicated via e-mail, internet telephony, face to face, and by telephone as required.

### PICO questions

The Task Force members suggested and reviewed topics and questions. Topics were reviewed for areas of controversy, known additional new science, and subject matter not previously evaluated. Finally, 22 therapy population [P], intervention [I], comparator [C], and outcome [O] (PICO) questions [9] were addressed in these recommendations.

### Inclusion and exclusion criteria

#### Search strategy

After identifying and prioritizing the PICO questions to be addressed, an initial search of PubMed, Embase, and the Cochrane Library for articles published until July 01, 2019 was performed. After the initial data was compiled, a refresh repeat search until September 31, 2020 was performed. Both searches were performed with the assistance of information specialists. The “related articles” function and manual review of bibliographies were also used to broaden the searches.

#### Study selection

The Task Force members accessed all abstracts and assessed general relevance. Reviews, viewpoints, and technical papers were excluded. We included prospective randomized trials, case control studies, prospective observational studies, retrospective observational trials, and cohort studies with comparator groups without language restrictions. Inclusion of case reports depended on the availability of other evidence for that particular PICO question.

#### Data extraction and management

The Task Force performed a detailed systematic review based on the Grading of Recommendations, Assessment, Development, and Evaluation (GRADE) system [10]. Data were entered into GRADEpro (Version 3.2, Cochrane Collaboration, Oxford) to generate quality of evidence tables.

#### Assessment of methodological quality and recommendations

The reviewers for each question (Additional File 1) created a reconciled risk of bias assessment for each of the included studies [11–13]. The quality of the evidence (or confidence in the estimate of the effect) was categorized as high, moderate, low, or very low [14], based on the study methodologies and the five core GRADE domains of risk of bias, inconsistency, indirectness, imprecision, and other considerations (including publication bias) [15]. The GRADE evidence profile tables were used to evaluate the evidence in support of each of the critical and important outcomes, create a written summary of evidence for each outcome (the consensus on science statements), and create consensus-based treatment recommendations [16]. These were accompanied by an overall assessment of the evidence and a statement from the Task Force about the values and preferences that underlie the recommendations.

Although we used the GRADE system to perform a more comprehensive assessment of the certainty of evidence, the recommendations and scientific strength were presented with the ACC/AHA Clinical Practice Guideline Recommendation Classification System [17] (Table 1). We used this system to ensure the comprehensive, objective assessment of all available evidence and delivery of recommendations in a uniform format that is most useful at the point of care. Also, this process is more effective in updating recommendations as new evidence emerges, and creation of tools to integrate context-sensitive guideline recommendations with electronic health records [17–19].

**Table 1:** American college of cardiology/American heart association recommendation system

Class (strength) of recommendation	
*Class I (Strong)—Benefit* >>> *Risk*	
Suggested phrases for writing recommendations:	
Is recommended Is indicated/useful/effective/beneficial Should be performed/administered/other Comparative-Effectiveness Phrases	
Treatment/strategy A is recommended/indicated in preference to treatment B Treatment A should be chosen over treatment B	
*Class IIa (Moderate)—Benefit* >> *Risk*	
Suggested phrases for writing recommendations:	
Is reasonable Can be useful/effective/beneficial Should be performed/administered/other Comparative-Effectiveness Phrases	
Treatment/strategy A is probably recommended/indicated in preference to treatment B It is reasonable to choose treatment A over treatment B	
*Class IIb (Weak)—Benefit* ≥ *Risk*	
Suggested phrases for writing recommendations:	
May/might be reasonable May/might be considered Usefulness/effectiveness is unknown/unclear/uncertain or not well established	
*Class III: No Benefit (Moderate)—Benefit* = *Risk*	
Suggested phrases for writing recommendations:	
Is not recommended Is not indicated/useful/effective/beneficial Should not be performed/administered/other	
*Class III: Harm (Strong)—Benefit* < *Risk*	
Suggested phrases for writing recommendations:	
Potentially harmful Causes harm Associated with excess morbidity/mortality Should not be performed/administered/other	
*Level (Quality) of evidence*	
*Level A*	
High-quality evidence from more than 1 RCT Meta-analyses of high-quality RCTs One or more RCTs corroborated by high-quality registry studies	
*Level B-R (randomized)*	
Moderate-quality evidence from 1 or more RCTs Meta-analyses of moderate-quality RCTs	
*Level B-NR (nonrandomized)*	
Moderate-quality evidence from 1 or more well-designed, well-executed nonrandomized studies, observational studies, or registry studies Meta-analyses of such studies	
*Level C-LD (limited data)*	
Randomized or nonrandomized observational or registry studies with limitations of design or execution Meta-analyses of such studies Physiological or mechanistic studies in human subjects	
*Level C-EO (expert opinion)*	
Consensus of expert opinion based on clinical experience	

*COR* class of recommendation, *EO* expert opinion, *LD* limited data, *LOE* level of evidence, *NR* nonrandomized, *R* randomized and *RCT* randomized clinical trial

COR and LOE are determined independently (any COR may be paired with any LOE). A recommendation with LOE C does not simply imply that the recommendation is weak. Many important clinical questions addressed in guidelines do not lend themselves to clinical trials. Although RCTs are unavailable, there may be a very clear clinical consensus that a particular test or therapy is useful or effective

Modified from reference [17]

### Modified Delphi consensus validation

A Delphi round was used to assess the strength of consensus of the 22 statements. The Task Force members were asked to vote and either agree or disagree with each recommendation. Based on published consensus papers, an agreement amongst ≥ 70% of experts was construed as consensus [20–22]. Voting was conducted virtually on Typeform® without identifying individual members’ responses. The voting link was made live on 25 January 2021. All the participants could comment on the statements. After the first round and comments of the participants, the wording of the strength of recommendation for one statement was changed, while the wording, but not the content, of two other statements was changed.

A second-round voting was conducted on 3 February 2021. All comments from the first round were available to the participants in the second round. The Task Force voted again on the 22 statements including one statement where the agreement was ≥ 60.0% but was not enough to reach the consensus threshold of 70%. All questions reached more than 70% consensus from participants after two rounds. The final recommendations and level of agreement are provided in Additional File 2.

## Results

### Search results

Full details of the search results and GRADE analysis are presented in Additional File 3.

## Summary of PERIOPCA recommendations

### ***ETCO***_***2***_*** as a prognosis tool of cardiac arrest***

- In patients with PERIOPCA, it may be reasonable to maintain an ETCO_2_ ≥ 10 mmHg during advanced life support. However, ETCO_2_ should be evaluated in the context of the patient’s clinical status and individualized targets may be necessary considering the cause of arrest, the degree of hypoxia, the quality of CPR and time to ROSC (COR/LOE: IIb/C-EO).

### Monitoring physiological parameters during CPR

- In adults with cardiac arrest in the perioperative setting, the use of physiological feedback may be reasonable to increase CPR quality and improve short- and long-term outcome (COR/LOE: IIb/C-EO).

### Chest compression or defibrillation strategy for ventricular fibrillation (VF) or pulseless ventricular tachycardia (pVT)

- In adult patients with PERIOPCA, ventricular fibrillation/pulseless ventricular tachycardia should be defibrillated within 3 min after the onset of the arrest (COR/LOE: I/C-LD). The use of AEDs in patients with ventricular fibrillation/pulseless ventricular tachycardia can be useful for improving survival (COR/LOE: IIa/C-LD). It is not recommended to defibrillate patients with ventricular fibrillation/pulseless ventricular tachycardia lasting more than 3 min without prior chest compressions (COR/LOE: III/C-LD).

### Timing of administration of epinephrine

- In adult patients with PERIOPCA, epinephrine administration after the 3rd shock can be beneficial (COR/LOE: IIa/C-LD).

### Standard-dose epinephrine (SDE) versus low-dose epinephrine (LDE) or high-dose epinephrine (HDE)

- In patients with PERIOPCA, it may be reasonable to administer 1 mg epinephrine for improving coronary perfusion pressure (COR/LOE: IIb/C-EO).

### No vasopressor versus epinephrine, or vasopressin

- In patients with PERIOPCA, it may be reasonable to administer epinephrine every 3–5 min (COR/LOE: IIb/C-EO).

### Antiarrhythmic drugs for cardiac arrest

- In adult patients with PERIOPCA, it is recommended to administer amiodarone or lidocaine for the treatment of ventricular fibrillation/pulseless ventricular tachycardia (COR/LOE: I/C-LD). Magnesium is not indicated for the treatment of ventricular fibrillation/pulseless ventricular tachycardia in the perioperative setting (COR/LOE: III/C-LD).

### Timing of administration of anti-arrhythmic

- In adult patients with perioperative ventricular fibrillation/pulseless ventricular tachycardia, it might be reasonable to administer amiodarone or lidocaine after the 3^rd^ shock (COR/LOE: IIb/C-EO).

### Ventilation rate during continuous chest compressions

- In adult patients with PERIOPCA and a secure airway, a ventilation rate of 10 breaths/min during CPR may be reasonable (COR/LOE: IIb/C-EO).

### Cardiac arrest associated with pulmonary embolism

- In adult patients with PERIOPCA due to pulmonary embolism or suspected pulmonary embolism, early consideration of thrombolysis and CPR duration of at least 60–90 min with or without the use of a mechanical chest compression device may be reasonable before terminating resuscitation attempts (COR/LOE: IIb/C-LD). The emergency treatment option among fibrinolytic therapy, surgical, or mechanical thrombectomy should be selected based on timing and available expertise, since no clear benefit of one approach over the other has been demonstrated.

### Cardiac arrest during pregnancy

- In pregnant women with PERIOPCA, the effectiveness of any special interventions, compared to standard measures, is uncertain, except probably for manual uterine displacement during chest compressions (COR/LOE: IIb/C-EO). In pregnant women with PERIOPCA due to suspected or proven pulmonary embolism, it may be reasonable to use thrombolysis or other measures to remove clot (e.g., surgical or percutaneous pulmonary embolectomy) (COR/LOE: IIb/C-EO). Extracorporeal membrane oxygenation may be considered as an acceptable salvage therapy for pregnant and postpartum patients with PERIOPCA or those with critical cardiac or pulmonary illness (COR/LOE: IIb/C-EO).

### Opioid toxicity

- In patients with PERIOPCA due to opioid toxicity, it might be reasonable to administer specific agents in addition to advanced life support (COR/LOE: IIb/C-EO).

### Epinephrine, vasopressin, steroids, and their combination during or after CPR

- In adult patients with PERIOPCA, it is reasonable to administer corticosteroid or mineralocorticoid or the combination of vasopressin, epinephrine, and steroids during/after CPR to increase ROSC (COR/LOE: IIa/B-R). In these patients, these drugs can be useful for improving survival to discharge with good functional outcome (COR/LOE: IIa/B-R).

### Lipid therapy for cardiac arrest

- In adult patients with PERIOPCA due to confirmed or suspected LAST, it may be reasonable to use lipid therapy (COR/LOE: IIb/C-LD).

### Ultrasound during CPR

- In patients with PERIOPCA, it may be reasonable to use point-of-care ultrasound to improve CPR and increase survival rates (COR/LOE: IIb/C-EO).

### ECPR versus manual or mechanical CPR

- In adult patients with PERIOPCA, it may be reasonable to use ECPR as a rescue therapy when CPR has failed to provide ROSC or non-sustained ROSC (COR/LOE: IIb/C-LD).

### Postresuscitation hemodynamic support

- In patients with ROSC after PERIOPCA, it may be reasonable to target the hemodynamics goals to optimize tissue perfusion as indicated by an adequate urine output (1 ml kg^−1^ h^−1^) and normal or decreasing plasma lactate values, taking into consideration the patient’s normal blood pressure, the cause of the arrest and the severity of any myocardial dysfunction (COR/LOE: IIb/C-EO).

### Postresuscitation antiarrhythmic drugs

- In the perioperative setting, it may be reasonable to administer antiarrhythmics immediately after ROSC to treat postresuscitation arrhythmias, especially in refractory cases, and prevent recurrences (COR/LOE: IIb/C-EO).

### Postresuscitation permissive hypercapnia

- In patients with ROSC after PERIOPCA, a lung-protective ventilation strategy (reducing tidal volume, plateau pressure, and driving pressure) and mild hypercapnia (PaCO_2_ of 40–50 mmHg) might be reasonable for improving outcome (COR/LOE: IIb/C-EO).

### ***Postresuscitation target of PaO***_***2***_

- In patients with PERIOPCA, it may be reasonable to maintain normoxemia and avoid hyperoxemia (PaO_2_ goal of < 200 mmHg) in order to improve short and long-term outcome (COR/LOE: IIb/C-EO).

### Targeted temperature management

- In comatose patients with PERIOPCA, it may be reasonable to maintain normothermia in order to improve short and long-term outcome (COR/LOE: IIb/C-EO). Potential neurological benefit should be balanced against the hemorrhagic risk related to hypothermia (< 37 °C) in this surgical setting.

### Prognostication in comatose patients treated with hypothermic targeted temperature management

- In patients with PERIOPCA and ROSC, it may be reasonable to use a multimodal strategy for prognostication, giving emphasis on allowing sufficient time for neurological recovery and to enable sedatives/paralytics to be cleared (COR/LOE: IIb/C-EO).

## PERIOPCA recommendations

The full list of PERIOPCA recommendations, including a short introduction, consensus on science, treatment recommendations, and values, preferences, and task force insights are provided in Additional File 4.

## Discussion

Perioperative cardiac arrest may be a predictable event in the critically ill patient undergoing emergency surgery or it may occur suddenly in reasonably healthy patients due to an unknown predisposing factor, such as genetic susceptibility to malignant hyperthermia or unrecognized iatrogenic complication of anesthesia or surgical procedure. The most common cause of anesthesia-related cardiac arrest involves airway management [3, 23, 24], but most PERIOPCAs are due to non-anesthetic causes. The heterogeneous data and nature of perioperative setting (patient comorbidities, surgical procedures) are the main reason for the absence of a universal definition of PERIOPCA [23]. However, patients in the operating room are usually fully monitored and therefore, cardiac arrest is diagnosed early.

In this multinational effort, a consensus on 22 PICO questions specially formulated for the perioperative setting was achieved for the first time. The main limitation of our work lies in the quality and quantity of available evidence. Most of our PICO recommendations about PERIOPCA management derive from observational data, such as case series and case reports, and physiopathology-based reasoning. This is justified by the association of PERIOPCA with emergency surgery on fragile patients, making difficult to design and perform clinical studies on this subject. The data in this setting remain limited and as in other Resuscitation guidelines, these recommendations are based on expert opinion. However, expert advice remains the only tool for clinicians to base their decisions on in areas where robust evidence is lacking [25]. The PERIOPCA recommendations are strengthened by a strict methodology including a modified Delphi consensus-building strategy and are useful for routine decision making using an evidence-based pragmatic course of action. The modified Delphi approach is more robust than traditional consensus-building approaches, and have been successfully used before in other clinical settings [20, 21, 25]. Each of the 22 statements summarizes the Task Force’s expert interpretation of all relevant data and its consensus treatment recommendations.

## Conclusions

In this first multinational attempt at consensus building on PERIOPCA, experts from different countries achieved consensus on 22 PICO questions. These recommendations provide a basis for decision making, as well as a guide for future research on PERIOPCA.

## Supplementary Information

**Additional file 1**: PICO questions and reviewers.

**Additional file 2**: Final recommendations and level of agreement.

**Additional file 3**: Full details of the search results and GRADE analysis.

**Additional file 4**: The full list of PERIOPCA recommendations, including a short introduction, consensus on science, treatment recommendations, and values, preferences, and task force insights.

## Data Availability

All data generated or analyzed during this study are included in this published article (and its supplementary information files).

## Abbreviations

ETCO_2_: End-tidal carbon dioxide
CPR: Cardiopulmonary resuscitation
AED: Automated external defibrillator
ROSC: Return of spontaneous circulation
LAST: Local anesthetic systemic toxicity
ECPR: Extracorporeal cardiopulmonary resuscitation
PaCO_2_: Arterial partial pressure of carbon dioxide
PaO_2_: Arterial partial pressure of oxygen
