# Supplementary material for: Clinical practice recommendations on the management of perioperative cardiac arrest: A report from the PERIOPCA Consortium
Source: Crit Care. 2021 Jul 29;25:265. doi: 10.1186/s13054-021-03695-2 (PMC8323279; doi:10.1186/s13054-021-03695-2)
Supplement: Supplementary file 1 — Additional file 1: PICO questions and reviewers. [file 13054_2021_3695_MOESM1_ESM.docx]

| **PERIOPCA PICOs**  **Perioperative setting: Operating Room, Post-anesthesia Care Unit, and Intensive Care Unit** | | | | |
| --- | --- | --- | --- | --- |
| **PICO ID** | **Evidence reviewers** | **Short title** | **PICO question** | **Included studies** |
| 1 | **Vladimir Cerny** | **ETCO_2_ as a prognosis tool of cardiac arrest** | Among adults who are in cardiac arrest in the perioperative setting (P), does any ETCO_2_ level value, when present (I), compared with any ETCO_2_ level below that value (C), change survival with favorable neurologic/functional outcome at discharge, 30 days, 60 days, 180 days, and/or 1 year; survival only at discharge, 30 days, 60 days, 180 days, and/or 1 year; ROSC (O)? | **-** |
| 2 | **Athanasios Chalkias, Theodoros Xanthos** | Monitoring physiological parameters during CPR | Among adults who are in cardiac arrest in the perioperative setting (P), does the use of physiological feedback regarding CPR quality (e.g., arterial lines, ETCO_2_ monitoring, SpO_2_ waveforms, or others) (I), compared with no feedback (C), change survival with favorable neurologic/functional outcome at discharge, 30 days, 60 days, 180 days, and/or 1 year; survival only at discharge, 30 days, 60 days, 180 days, and/or 1 year; ROSC; change in physiologic values by modifications in CPR (O)? | **1. Maillard J, Sologashvili T, Diaper J, Licker MJ, Keli Barcelos G. A Case of Persistence of Normal Tissue Oxygenation Monitored by Near-Infrared Spectroscopy (NIRS) Values Despite Prolonged Perioperative Cardiac Arrest. Am J Case Rep 2019;20:21-25.**  **2. Paarmann H, Heringlake M, Sier H, Schön J. The association of non-invasive cerebral and mixed venous oxygen saturation during cardiopulmonary resuscitation. Interact Cardiovasc Thorac Surg 2010;11:371-373.**  **3. Yunoki K, Sasaki R, Taguchi A, Maekawa S, Ueta H, Yamazaki K. Successful recovery without any neurological complication after intraoperative cardiopulmonary resuscitation for an extended period of time in the lateral position: a case report. JA Clin Rep 2016;2:7.** |
| 3 | **Vladimir Lomivorotov, Vladimir Boboshko** | Chest compression or Defibrillation strategy for VF or pVT | Among adults who are in ventricular fibrillation or pulseless ventricular tachycardia in the perioperative setting (P), does any interval of CPR first (e.g., 2 min) (I), compared with defibrillation first (C), change survival with favorable neurological/functional outcome at discharge, 30 days, 60 days, 180 days and/or 1 year, survival only at discharge, 30 days, 60days, 180 days and/or 1 year, ROSC, termination of arrhythmia (O)? | **1. Peberdy MA, Kaye W, Ornato JP, Larkin GL, Nadkarni V, Mancini ME, Berg RA, Nichol G, Lane-Trultt T. Cardiopulmonary resuscitation of adults in the hospital: a report of 14720 cardiac arrests from the National Registry of Cardiopulmonary Resuscitation. Resuscitation 2003;58:297-308.**  **2. Skogvoll E, Nordseth T. The early minutes of in-hospital cardiac arrest: shock or CPR? A population based prospective study. Scand J Trauma Resusc Emerg Med 2008;16:11.**  **3. Chan PS, Krumholz HM, Nichol G, Nallamothu BK; American Heart Association National Registry of Cardiopulmonary Resuscitation Investigators. Delayed time to defibrillation after in-hospital cardiac arrest. N Engl J Med 2008;358:9-17.**  **4. Chan PS, Krumholz HM, Spertus JA, Jones PG, Cram P, Berg RA, Peberdy MA, Nadkarni V, Mancini ME, Nallamothu BK; American Heart Association National Registry of Cardiopulmonary Resuscitation (NRCPR) Investigators. Automated external defibrillators and survival after in-hospital cardiac arrest. JAMA 2010;304:2129-2136.**  **5. Davis D, Aguilar SA, Sell R, Minokadeh A, Husa R. A focused investigation of expedited, stack of three shocks versus chest compressions first followed by single shocks for monitored ventricular fibrillation/ventricular tachycardia cardiopulmonary arrest in an in-hospital setting. J Hosp Med 2016;11:264-268.** |
| 4 | **Athanasios Chalkias, Theodoros Xanthos** | Timing of administration of epinephrine | Among adults who are in cardiac arrest in the perioperative setting (P), does early epinephrine delivery by IV or IO route (e.g., less than 10 min after the beginning of resuscitation) (I), compared with delayed timing of epinephrine delivery (e.g., more than 10 min after the beginning of resuscitation) (C), change survival with favorable neurologic/functional outcome at discharge, 30 days, 60 days, 180 days, and/or 1 year; survival only at discharge, 30 days, 60 days, 180 days, and/or 1 year; ROSC (O)? | **1. Bircher NG, Chan PS, Xu Y, American Heart Association's Get With The Guidelines-Resuscitation I. Delays in Cardiopulmonary Resuscitation, Defibrillation, and Epinephrine Administration All Decrease Survival in In-hospital Cardiac Arrest. Anesthesiology 2019;130:414-422.**  **2. Andersen LW, Kurth T, Chase M, Berg KM, Cocchi MN, Callaway C, Donnino MW; American Heart Association’s Get With The Guidelines-Resuscitation Investigators. Early administration of epinephrine (adrenaline) in patients with cardiac arrest with initial shockable rhythm in hospital: propensity score matched analysis. BMJ 2016;353:i1577.** |
| 5 | **Athanasios Chalkias, Theodoros Xanthos** | Standard-dose epinephrine vs. low-dose epinephrine or high-dose epinephrine | In adult patients in cardiac arrest in the perioperative setting (P), does low-dose epinephrine (< 1 mg) or high-dose epinephrine (> 1mg) (I), compared with standard-dose epinephrine (1 mg bolus dose) (C), change survival to 180 days with good neurologic outcome, survival to 180 days, survival to hospital discharge with good neurologic outcome, survival to hospital discharge, ROSC (O)? | **-** |
| 6 | **Athanasios Chalkias, Theodoros Xanthos** | **No vasopressor versus epinephrine, or vasopressin** | Among adults who are in cardiac arrest in the perioperative setting (P), does avoiding the use of any vasopressor during CPR (I), compared with using epinephrine alone or vasopressin alone, or vasopressin in combination with epinephrine (C), change survival with favorable neurologic/functional outcome at discharge, 30 days, 60 days, 180 days, and/or 1 year; survival only at discharge, 30 days, 60 days, 180 days, and/or 1 year (O)? | **-** |
| 7 | **Vladimir Lomivorotov, Vladimir Boboshko** | Antiarrhythmic drugs for cardiac arrest | Among adults who are in cardiac arrest in the perioperative setting (P), does administration of antiarrhythmic drugs (e.g., amiodarone, lidocaine, other) (I), compared with not using antiarrhythmic drugs (no drug or placebo) (C), change survival with favorable neurologic/functional outcome at discharge, 30 days, 60 days, 180 days, and/or 1 year; survival only at discharge, 30 days, 60 days, 180 days, and/or 1 year; ROSC (O)? | **1. Thel MC, Armstrong AL, McNulty SE, Califf RM, O'Connor CM. Randomised trial of magnesium in in-hospital cardiac arrest. Duke Internal Medicine Housestaff. Lancet 1997;350:1272-1276.**  **2. Pollak PT, Wee V, Al-Hazmi A, Martin J, Zarnke KB. The use of amiodarone for in-hospital cardiac arrest at two tertiary care centres. Can J Cardiol 2006;22:199-202.**  **3. Rea RS, Kane-Gill SL, Rudis MI, Seybert AL, Oyen LJ, Ou NN, Stauss JL, Kirisci L, Idrees U, Henderson SO. Comparing intravenous amiodarone or lidocaine, or both, outcomes for inpatients with pulseless ventricular arrhythmias. Crit Care Med 2006;34:1617-1623.**  **4. Shiga T, Tanaka K, Kato R, Amino M, Matsudo Y, Honda T, Sagara K, Takahashi A, Katoh T, Urashima M, Ogawa S, Takano T, Kasanuki H; Refractory VT/VF, Prospective Evaluation to Differentiate Lidocaine Efficacy from Nifekalant (RELIEF) Study Investigators. Nifekalant versus lidocaine for in-hospital shock-resistant ventricular fibrillation or tachycardia. Resuscitation 2010;81:47-52.** |
| 8 | **Vladimir Lomivorotov, Vladimir Boboshko** | Timing of administration of anti-arrhythmic | Among adults who are in cardiac arrest in the perioperative setting (P), does early antiarrhythmic (e.g., amiodarone, lidocaine, other) delivery by IV or IO route (e.g., less than 6 min after the beginning of resuscitation) (I), compared with delayed timing of antiarrhythmic delivery (e.g., more than 6 min after the beginning of resuscitation) (C), change survival with favorable neurologic/functional outcome at discharge, 30 days, 60 days, 180 days, and/or 1 year; survival only at discharge, 30 days, 60 days, 180 days, and/or 1 year; ROSC (O)? | **-** |
| 9 | **Giuseppe Ristagno, Francesca Fumagalli, Aurora Magliocca** | Ventilation rate during continuous chest compressions | Among adults with cardiac arrest with a secure airway receiving chest compressions (in the perioperative setting, and with standard tidal volume) (P), does a ventilation rate of 10 breaths/min (I), compared with any other ventilation rate (C), change survival with favorable neurologic/functional outcome at discharge, 30 days, 60 days, 180 days, and/or 1 year; survival only at discharge, 30 days, 60 days, 180 days, and/or 1 year; ROSC (O)? | **-** |
| 10 | **Giuseppe Ristagno, Francesca Fumagalli, Aurora Magliocca** | Cardiac arrest associated with pulmonary embolism | Among adults who are in cardiac arrest due to pulmonary embolism or suspected pulmonary embolism in the perioperative setting (P), does any specific alteration in treatment algorithm (e.g., fibrinolytics, or any other) (I), compared with standard care (C), change survival with favorable neurologic/functional outcome at discharge, 30 days, 60 days, 180 days, and/or 1 year; survival only at discharge, 30 days, 60 days, 180 days, and/or 1 year; ROSC (O)? | **-** |
| 11 | **Vladimir Cerny** | Cardiac arrest during pregnancy | Among pregnant women who are in cardiac arrest in the perioperative setting (P), do any specific interventions (I), compared with standard care (usual resuscitation practice) (C), change survival with favorable neurologic/functional outcome at discharge, 30 days, 60 days, 180 days, and/or 1 year; survival only at discharge, 30 days, 60 days, 180 days, and/or 1 year; ROSC (O)? | **-** |
| 12 | **Gabriele Finco, Salvatore Sardo, Paolo Mura** | Opioid toxicity | Among adults who are in cardiac arrest or respiratory arrest due to opioid toxicity in the perioperative setting (P), does any specific therapy (e.g., naloxone, bicarbonate, or other drugs) (I), compared with usual ALS (C), change survival with favorable neurologic/functional outcome at discharge, 30 days, 60 days, 180 days, and/or 1 year; survival only at discharge, 30 days, 60 days, 180 days, and/or 1 year; ROSC (O)? | **-** |
| 13 | **Athanasios Chalkias, Theodoros Xanthos** | Epinephrine, vasopressin, steroids, and their combination during or after CPR | Among adults who are in cardiac arrest in the perioperative setting (P), does corticosteroid or mineralocorticoid administration during and/or after CPR or the combined use of vasopressin, epinephrine, and steroids during and/or after CPR (I), compared with not using steroids or epinephrine alone during CPR and no steroids after CPR (C), change survival with favorable neurologic/functional outcome at discharge, 30 days, 60 days, 180 days, and/or 1 year; survival only at discharge, 30 days, 60 days, 180 days, and/or 1 year; ROSC (O)? | **1. Mentzelopoulos SD, Malachias S, Chamos C, Konstantopoulos D, Ntaidou T, Papastylianou A, Kolliantzaki I, Theodoridi M, Ischaki H, Makris D, Zakynthinos E, Zintzaras E, Sourlas S, Aloizos S, Zakynthinos SG. Vasopressin, steroids, and epinephrine and neurologically favorable survival after in-hospital cardiac arrest: a randomized clinical trial. JAMA.2013;310:270-279.**  **2. Mentzelopoulos SD, Zakynthinos SG, Tzoufi M, Katsios N, Papastylianou A, Gkisioti S, Stathopoulos A, Kollintza A, Stamataki E, Roussos C. Vasopressin, epinephrine, and corticosteroids for in-hospital cardiac arrest. Arch Intern Med 2009;169:15-24.** |
| 14 | **Gabriele Finco, Salvatore Sardo, Paolo Mura** | Lipid therapy for cardiac arrest | In adult patients with cardiac arrest due to suspected drug toxicity (e.g., local anesthetics, tricyclic antidepressants, others) in the perioperative setting (P), does administration of IV lipid (I), compared with no IV lipid (C), change survival with favorable neurologic/functional outcome at discharge, 30 days, 60 days, 180 days, and/or 1 year; survival only at discharge, 30 days, 60 days, 180 days, and/or 1 year; ROSC (O)? | **1. Rosenblatt MA, Abel M, Fischer GW, Itzkovich CJ, Eisenkraft JB. Successful use of a 20% lipid emulsion to resuscitate a patient after a presumed bupivacaine-related cardiac arrest. Anesthesiology 2006;105:217-218.**  **2. Smith HM, Jacob AK, Segura LG, Dilger JA, Torsher LC. Simulation education in anesthesia training: a case report of successful resuscitation of bupivacaine-induced cardiac arrest linked to recent simulation training. Anesth Analg 2008;106:1581-1584, table of contents.**  **3. Marwick PC, Levin AI, Coetzee AR. Recurrence of cardiotoxicity after lipid rescue from bupivacaine-induced cardiac arrest. Anesth Analg 2009;108:1344-1346.**  **4. Whiteman DM, Kushins SI. Successful Resuscitation With Intralipid After Marcaine Overdose. Aesthet Surg J 2014;34:738-740.**  **5. Weber F, Guha R, Weinberg G, Steinbach F, Gitman M. Prolonged Pulseless Electrical Activity Cardiac Arrest After Intranasal Injection of Lidocaine With Epinephrine: A Case Report A A Pract 2019;12:438-440.**  **6. Hasan B, Asif T, Hasan M. Lidocaine-Induced Systemic Toxicity: A Case Report and Review of Literature. Cureus 2017;9:e1275.**  **7. Gnaho A, Eyrieux S, Gentili M. Cardiac arrest during an ultrasound-guided sciatic nerve block combined with nerve stimulation. Reg Anesth Pain Med 2009;34:278.**  **8. Warren JA, Thoma RB, Georgescu A, Shah SJ. Intravenous lipid infusion in the successful resuscitation of local anesthetic-induced cardiovascular collapse after supraclavicular brachial plexus block. Anesth Analg 2008;106:1578-1580, table of contents.**  **9. Sonsino DH, Fischler M. Immediate intravenous lipid infusion in the successful resuscitation of ropivacaine-induced cardiac arrest after infraclavicular brachial plexus block. Reg Anesth Pain Med 2009;34:276-277.**  **10. Scherrer V, Compere V, Loisel C, Dureuil B. Cardiac arrest from local anesthetic toxicity after a field block and transversus abdominis plane block: a consequence of miscommunication between the anesthesiologist and surgeon. A A Case Rep 2013;1:75-76.**  **11. Mazoit JX. Arrêt cardiaque et anesthésiques locaux [Cardiac arrest and local anaesthetics]. Presse Med 2013;42:280-286.**  **12. Litz RJ, Popp M, Stehr SN, Koch T. Successful resuscitation of a patient with ropivacaine-induced asystole after axillary plexus block using lipid infusion. Anaesthesia 2006;61:800-801.**  **13. Markowitz S, Neal JM. Immediate lipid emulsion therapy in the successful treatment of bupivacaine systemic toxicity. Reg Anesth Pain Med 2009;34:276.** |
| 15 | **Athanasios Chalkias, Theodoros Xanthos** | Ultrasound during CPR | Among adults who are in cardiac arrest in the perioperative setting (P), does use of ultrasound (including echocardiography or other organ assessments) during CPR (I), compared with conventional CPR and resuscitation without use of ultrasound (C), change survival with favorable neurologic/functional outcome at discharge, 30 days, 60 days, 180 days, and/or 1 year; survival only at discharge, 30 days, 60 days, 180 days, and/or 1 year; ROSC (O)? | **-** |
| 16 | **Athanasios Chalkias, Theodoros Xanthos** | ECPR versus manual or mechanical CPR | Among adults who are in cardiac arrest in the perioperative setting (P), does the use of ECPR techniques (including extracorporeal membrane oxygenation or cardiopulmonary bypass) (I), compared with manual CPR or mechanical CPR (C), change survival with favorable neurologic/functional outcome at discharge, 30 days, 60 days, 180 days, and/or 1 year; survival only at discharge, 30 days, 60 days, 180 days, and/or 1 year; ROSC (O)? | **1. Lin JW, Wang MJ, Yu HY, Wang CH, Chang WT, Jerng JS, Huang SC, Chou NK, Chi NH, Ko WJ, Wang YC, Wang SS, Hwang JJ, Lin FY, Chen YS. Comparing the survival between extracorporeal rescue and conventional resuscitation in adult in-hospital cardiac arrests: propensity analysis of three-year data. Resuscitation 2010;81:796-803.**  **2. Avalli L, Maggioni E, Formica F, Redaelli G, Migliari M, Scanziani M, Celotti S, Coppo A, Caruso R, Ristagno G, Fumagalli R. Favourable survival of in-hospital compared to out-of-hospital refractory cardiac arrest patients treated with extracorporeal membrane oxygenation: an Italian tertiary care centre experience. Resuscitation 2012;83:579-583.** |
| 17 | **Athanasios Chalkias, Theodoros Xanthos** | Postresuscitation hemodynamic Support | Among adults with ROSC after cardiac arrest in the perioperative setting (P), does titration of therapy to achieve a specific hemodynamic goal (e.g., MAP greater than 65 mm Hg) (I), compared with no hemodynamic goal (C), change survival with favorable neurologic/functional outcome at discharge, 30 days, 60 days, 180 days, and/or 1 year; survival at discharge, 30 days, 60 days, 180 days, and/or 1 year (O)? | **-** |
| 18 | **Athanasios Chalkias, Theodoros Xanthos** | Postresuscitation antiarrhythmic drugs | Among adults with ROSC after cardiac arrest in the perioperative setting (P), do prophylactic antiarrhythmic drugs given immediately after ROSC (I), compared with not giving antiarrhythmic drugs (C), change survival with favorable neurologic/functional outcome at discharge, 30 days, 60days, 180 days, and/or 1 year; development of cardiac arrest; survival only at discharge, 30 days, 60 days, 180 days, and/or 1 year; recurrence of ventricular fibrillation/pulseless ventricular tachycardia; incidence of arrhythmias (O)? | **-** |
| 19 | **Gabriele Finco, Salvatore Sardo, Paolo Mura** | Postresuscitation permissive hypercapnia | Among successfully resuscitated perioperative cardiac arrest patients who receive mechanical ventilation (P), does permissive hypercapnia in the context of a lung-protective ventilatory strategy with low tidal volumes (i.e., 6 mL/kg predicted body weight) and PEEP/FiO_2_ set according to the ARDSnet protocol (I), compared with normocapnia in the context of a ventilatory strategy employing moderate tidal volumes (i.e., 7.5-10 mL/kg predicted body weight and PEEP levels of 5-10 cmH_2_O (C), change survival with favorable neurologic/functional outcome at discharge, 30 days, 60 days, 180 days, and/or 1 year; survival only at discharge, 30 days, 60 days, 180 days, and/or 1 year (O)? | **-** |
| 20 | **Nicolas Mongardon, Stéphane Legriel,**  **Anne-Laure Constant** | Postresuscitation target of PaO_2_ | Among adults with ROSC after cardiac arrest in the perioperative setting (P), does ventilation to a PaO_2_ goal of <200 mmHg (I), compared to PaO_2_ goal of >200 mmHg (C), change survival at discharge, 30days, 60 days, 180 days, and/or 1 year; survival with favorable neurologic/functional outcome at discharge, 30 days, 60 days, 180 days, and/or 1 year (O)? | **-** |
| 21 | **Nicolas Mongardon, Stéphane Legriel,**  **Anne-Laure Constant** | Targeted temperature management | In patients with ROSC after cardiac arrest in the perioperative setting (P), does induction of TTM (target temperature 32-36°C) for any duration or before some time point (e.g., 1 h after ROSC (I), compared with normothermia (C), change survival with favorable neurologic/functional outcome at discharge, 30 days, 60 days, 180 days, and/or 1 year; survival only at discharge, 30 days, 60 days, 180 days, and/or 1 year (O)? | **1. Constant AL, Mongardon N, Morelot Q, Pichon N, Grimaldi D, Bordenave L, Soummer A, Sauneuf B, Merceron S, Ricome S, Misset B, Bruel C, Schnell D, Boisramé-Helms J, Dubuisson E, Brunet J, Lasocki S, Cronier P, Bouhemad B, Carreira S, Begot E, Vandenbunder B, Dhonneur G, Jullien P, Resche-Rigon M, Bedos JP, Montlahuc C, Legriel S. Targeted temperature management after intraoperative cardiac arrest: a multicenter retrospective study. Intensive Care Med 2017;43:485-495.** |
| 22 | **Athanasios Chalkias, Theodoros Xanthos** | Prognostication in comatose patients treated with hypothermic targeted temperature management | Among adults with ROSC who are treated or not with TTM in the perioperative setting (P), does any clinical variable when abnormal (e.g., clinical exam, EEG, somatosensory evoked potentials [SSEPs], imaging, other) (I), compared with any clinical variable when normal (C), reliably predict death or poor neurologic outcome at discharge, 30 days, 60 days, 180 days, and/or 1year; death only at discharge, 30 days, 60 days, 180 days, and/or 1 year (O)? | **-** |

ETCO_2_, end-tidal carbon dioxide; CPR, cardiopulmonary resuscitation; SpO_2_, peripheral capillary oxygen saturation; VF, ventricular fibrillation; pVT, pulseless ventricular tachycardia; IV, intravenous; IO, intraosseous; ALS, advanced life support; ECPR, extracorporeal cardiopulmonary resuscitation; ROSC, return of spontaneous circulation; PEEP, positive end-expiratory pressure; FiO_2_, fraction of inspired oxygen; PaO_2_, arterial oxygen partial pressure; TTM, targeted temperature management; EEG, electroencephalography
