# Supplementary material for: Clinical practice recommendations on the management of perioperative cardiac arrest: A report from the PERIOPCA Consortium
Source: Crit Care. 2021 Jul 29;25:265. doi: 10.1186/s13054-021-03695-2 (PMC8323279; doi:10.1186/s13054-021-03695-2)
Supplement: Supplementary file 2 — Additional file 2: Final recommendations and level of agreement. [file 13054_2021_3695_MOESM2_ESM.docx]

Supplementary Table 2. Modified Delphi consensus validation

| **PICO** | **Recommendation** | **Level of agreement** |
| --- | --- | --- |
| **ETCO_2_ as a prognosis tool of cardiac arrest** | In patients with PERIOPCA, it may be reasonable to maintain an ETCO_2_ ≥ 10 mmHg during advanced life support. However, ETCO_2_ should be evaluated in the context of the patient’s clinical status and individualized targets may be necessary considering the cause of arrest, the degree of hypoxia, the quality of CPR and time to ROSC (COR/LOE: IIb/C-EO) | 100% |
| Monitoring physiological parameters during CPR | In adults with cardiac arrest in the perioperative setting, the use of physiological feedback may be reasonable to increase CPR quality and improve short- and long-term outcome (COR/LOE: IIb/C-EO) | 92.9% |
| Chest compression or defibrillation strategy for ventricular fibrillation or pulseless ventricular  tachycardia | In adult patients with PERIOPCA, ventricular fibrillation/pulseless ventricular tachycardia should be defibrillated within 3 minutes after the onset of the arrest (COR/LOE: I/C-LD). The use of AEDs in patients with ventricular fibrillation/pulseless ventricular tachycardia can be useful for improving survival (COR/LOE: IIa/C-LD). It is not recommended to defibrillate patients with ventricular fibrillation/pulseless ventricular tachycardia lasting more than 3 minutes without prior chest compressions (COR/LOE: III/C-LD) | 100% |
| Timing of administration of epinephrine | In adult patients with PERIOPCA, epinephrine administration after the 3^rd^ shock can be beneficial (COR/LOE: IIa/C-LD) | 92.9% |
| Standard-dose epinephrine vs. low-dose epinephrine or high-dose epinephrine | In patients with PERIOPCA, it may be reasonable to administer 1 mg epinephrine for improving coronary perfusion pressure (COR/LOE: IIb/C-EO) | 100% |
| **No vasopressor versus epinephrine, or vasopressin** | In patients with PERIOPCA, it may be reasonable to administer epinephrine every 3 to 5 minutes (COR/LOE: IIb/C-EO) | 100% |
| Antiarrhythmic drugs for cardiac arrest | In adult patients with PERIOPCA, it is recommended to administer amiodarone or lidocaine for the treatment of ventricular fibrillation/pulseless ventricular tachycardia (COR/LOE: I/C-LD). Magnesium is not indicated for the treatment of ventricular fibrillation/pulseless ventricular tachycardia in the perioperative setting (COR/LOE: III/C-LD) | 100% |
| Timing of administration of anti-arrhythmic | In adult patients with perioperative ventricular fibrillation/pulseless ventricular tachycardia, it might be reasonable to administer amiodarone or lidocaine after the 3^rd^ shock (COR/LOE: IIb/C-EO) | 100% |
| Ventilation rate during continuous chest compressions | In adult patients with PERIOPCA and a secure airway, a ventilation rate of 10 breaths/min during CPR may be reasonable (COR/LOE: IIb/C-EO) | 100% |
| Cardiac arrest associated with pulmonary embolism | In adult patients with PERIOPCA due to pulmonary embolism or suspected pulmonary embolism, early consideration of thrombolysis and CPR duration of at least 60-90 minutes with or without the use of a mechanical chest compression device may be reasonable before terminating resuscitation attempts (COR/LOE: IIb/C-LD). The emergency treatment option among fibrinolytic therapy, surgical, or mechanical thrombectomy should be selected based on timing and available expertise, since no clear benefit of one approach over the other has been demonstrated | 100% |
| Cardiac arrest during pregnancy | In pregnant women with PERIOPCA, the effectiveness of any special interventions, compared to standard measures, is uncertain, except probably for manual uterine displacement during chest compressions (COR/LOE: IIb/C-EO). In pregnant women with PERIOPCA due to suspected or proven pulmonary embolism, it may be reasonable to use thrombolysis or other measures to remove clot (e.g., surgical or percutaneous pulmonary embolectomy) (COR/LOE: IIb/C-EO). Extracorporeal membrane oxygenation may be considered as an acceptable salvage therapy for pregnant and postpartum patients with PERIOPCA or those with critical cardiac or pulmonary illness (COR/LOE: IIb/C-EO) | 100% |
| Opioid toxicity | In patients with PERIOPCA due to opioid toxicity, it might be reasonable to administer specific agents in addition to advanced life support (COR/LOE: IIb/C-EO) | 100% |
| Epinephrine, vasopressin, steroids, and their combination during or after CPR | In adult patients with PERIOPCA, it is reasonable to administer corticosteroid or mineralocorticoid or the combination of vasopressin, epinephrine, and steroids during/after CPR to increase ROSC (COR/LOE: IIa/B-R). In these patients, these drugs can be useful for improving survival to discharge with good functional outcome (COR/LOE: IIa/B-R) | 78.6% |
| Lipid therapy for cardiac arrest | In adult patients with PERIOPCA due to confirmed or suspected LAST, it may be reasonable to use lipid therapy (COR/LOE: IIb/C-LD) | 100% |
| Ultrasound during CPR | In patients with PERIOPCA, it may be reasonable to use point-of-care ultrasound to improve CPR and increase survival rates (COR/LOE: IIb/C-EO) | 92.9% |
| ECPR *vs.* manual or mechanical CPR | In adult patients with PERIOPCA, it may be reasonable to use ECPR as a rescue therapy when CPR has failed to provide ROSC or non-sustained ROSC (COR/LOE: IIb/C-LD) | 100% |
| Postresuscitation hemodynamic Support | In patients with ROSC after PERIOPCA, it may be reasonable to target the hemodynamics goals to optimize tissue perfusion as indicated by an adequate urine output (1 ml kg^−1^ h^−1^) and normal or decreasing plasma lactate values, taking into consideration the patient’s normal blood pressure, the cause of the arrest and the severity of any myocardial dysfunction (COR/LOE: IIb/C-EO) | 100% |
| Postresuscitation antiarrhythmic drugs | In the perioperative setting, it may be reasonable to administer antiarrhythmics immediately after ROSC to treat postresuscitation arrhythmias, especially in refractory cases, and prevent recurrences (COR/LOE: IIb/C-EO) | 100% |
| Postresuscitation permissive hypercapnia | In patients with ROSC after PERIOPCA, a lung-protective ventilation strategy (reducing tidal volume, plateau pressure, and driving pressure) and mild hypercapnia (PaCO_2_ of 40-50 mmHg) might be reasonable for improving outcome (COR/LOE: IIb/C-EO) | 92.9% |
| Postresuscitation target of PaO_2_ | In patients with PERIOPCA, it may be reasonable to maintain normoxemia and avoid hyperoxemia (PaO_2_ goal of <200 mmHg) in order to improve short and long-term outcome (COR/LOE: IIb/C-EO) | 100% |
| Targeted temperature management | In comatose patients with PERIOPCA, it may be reasonable to maintain normothermia in order to improve short and long-term outcome (COR/LOE: IIb/C-EO). Potential neurological benefit should be balanced against the hemorrhagic risk related to hypothermia (<37°C) in this surgical setting | 100% |
| Prognostication in comatose patients treated with hypothermic targeted temperature management | In patients with PERIOPCA and ROSC, it may be reasonable to use a multimodal strategy for prognostication, giving emphasis on allowing sufficient time for neurological recovery and to enable sedatives/paralytics to be cleared (COR/LOE: IIb/C-EO) | 100% |
