# Supplementary material for: Clinical practice recommendations on the management of perioperative cardiac arrest: A report from the PERIOPCA Consortium
Source: Crit Care. 2021 Jul 29;25:265. doi: 10.1186/s13054-021-03695-2 (PMC8323279; doi:10.1186/s13054-021-03695-2)
Supplement: Supplementary file 3 — Additional file 3: Full details of the search results and GRADE analysis. [file 13054_2021_3695_MOESM3_ESM.docx]

**Clinical practice recommendations on the management of perioperative cardiac arrest: A report from the PERIOPCA Consortium**

**SEARCH STRATEGIES AND STUDY SELECTION**

**PICO 1**

Among adults who are in cardiac arrest in the perioperative setting (P), does any ETCO_2_ level value, when present (I), compared with any ETCO_2_ level below that value (C), change survival with favorable neurologic/functional outcome at discharge, 30 days, 60 days, 180 days, and/or 1 year; survival only at discharge, 30 days, 60 days, 180 days, and/or 1 year; ROSC (O)?

**Search equation PUBMED**

"Heart Arrest"[Mesh:NoExp] OR "Cardiopulmonary Resuscitation"[Mesh:NoExp] OR “Cardiac arrest” [Title/Abstract] OR “Cardiac arrests” [Title/Abstract] OR “Heart Arrest” [Title/Abstract] OR “Heart Arrests” [Title/Abstract] OR “cardiopulmonary arrest"[Title/Abstract] OR “cardiopulmonary arrests”[Title/Abstract] OR "Cardiopulmonary resuscitation"[Title/Abstract] OR “Cardio Pulmonary resuscitation” [Title/Abstract] AND (((“end-tidal carbon dioxide”[Title/Abstract] OR “ETCO_2_”[Title/Abstract] OR "carbon dioxide"[Mesh] AND ("mortality"[Subheading] OR "mortality” [Title/Abstract] OR survival[Title/Abstract] OR "survival"[MeSH Terms] OR “return spontaneous circulation” [Title/Abstract] OR resuscitation[Title/Abstract] OR resuscitated[Title/Abstract] OR "Resuscitation"[Mesh] OR “Cerebral performance category” [Title/Abstract] OR "cerebral performance categories"[Title/Abstract] NOT "Out-of-Hospital Cardiac Arrest"[Mesh] OR “out of hospital”.

**Search equation EMBASE**

'heart arrest'/de OR 'cardiopulmonary arrest'/de OR 'cardiac arrest*':ti,ab OR 'heart arrest*':ti,ab OR 'cardiopulmonary arrest*':ti,ab OR 'cardiopulmonary resuscitation':ti,ab OR 'cardio pulmonary resuscitation':ti,ab AND 'mortality'/exp OR mortality:ti,ab OR survival:ti,ab OR 'survival'/exp OR 'return spontaneous circulation':ti,ab OR resuscitation:ti,ab OR resuscitated:ti,ab OR 'resuscitation'/exp AND 'end-tidal carbon dioxide ':ti,ab OR 'ETCO_2_':ti,ab OR 'carbon dioxide'/exp NOT 'out of hospital cardiac arrest'/exp OR 'out of hospital' OR 'out of hospitals'

**Search equation Cochrane Library**

“Cardiac arrest” OR “Cardiac arrests” OR “Heart Arrest” OR “Heart Arrests” OR “cardiopulmonary arrest" OR “cardiopulmonary arrests" in Title Abstract Keyword AND "Cardiopulmonary resuscitation" OR “Cardio Pulmonary resuscitation” in Title Abstract Keyword AND “end-tidal carbon dioxide” OR “etco2” OR “carbon dioxide” in Title Abstract Keyword - (Word variations have been searched)

**Study selection**

213 records screened

213 articles discarded (duplicates or excluded after title and abstract evaluation)

0 full-text article excluded due to absence of comparator

0 studies included in qualitative synthesis

**Characteristics of included studies**

| Study | Design | Patients | Interventions | Outcomes |
| --- | --- | --- | --- | --- |
| - | - | - | - | Primary: - |
|  |  |  |  | Secondary: - |

**Grade evidence table**

| **Outcomes** | **Quality assessment** | | | | | | | **Summary of findings** | | | | | | |
| --- | --- | --- | --- | --- | --- | --- | --- | --- | --- | --- | --- | --- | --- | --- |
|  |  |  |  |  |  |  |  | **No of patients** | | **Effect** | | | **Quality of evidence**  **(GRADE)** | |
|  | **No of participants**  **(studies)** | **Design** | **Limitations** | **Inconsistency** | **Indirectness** | **Imprecision** | **Other**  **considerations** | **ETCO_2_ level** | **No ETCO_2_ level** | **Relative**  **(95% CI)** | **Risk**  **difference with ETCO_2_ level** | **Absolute** |  |  |
| Survival with favorable neurologic/functional outcome at discharge, 30 days, 60 days, 180 days, and/or 1 year; survival only at discharge, 30 days, 60 days, 180 days, and/or 1 year; ROSC | – | - | - | - | - | - | - | - | - | - | - | - | - |  |

**Consensus of science statement**

In patients with PERIOPCA, it may be reasonable to maintain an ETCO_2_ ≥ 10 mmHg during advanced life support. However, ETCO_2_ should be evaluated in the context of the patient’s clinical status and individualized targets may be necessary considering the cause of arrest, the degree of hypoxia, the quality of CPR and time to ROSC (COR/LOE: IIb/C-EO).

**References**

None.

**PICO 2**

Among adults who are in cardiac arrest in the perioperative setting (P), does the use of physiological feedback regarding CPR quality (e.g., arterial lines, ETCO_2_ monitoring, SpO_2_ waveforms, or others) (I), compared with no feedback (C), change survival with favorable neurologic/functional outcome at discharge, 30 days, 60 days, 180 days, and/or 1 year; survival only at discharge, 30 days, 60 days, 180 days, and/or 1 year; ROSC; change in physiologic values by modifications in CPR (O)?

**Search equation PUBMED**

((((((("cardiac arrest"[Title/Abstract]) OR "cardiopulmonary arrest"[Title/Abstract]) OR "circulatory arrest"[Title/Abstract]) OR "heart arrest"[Title/Abstract])) AND (((((((((((((CPR[Title/Abstract]) OR "systolic arterial pressure"[Title/Abstract]) OR "diastolic arterial pressure"[Title/Abstract]) OR "mean arterial pressure"[Title/Abstract]) OR "systemic vascular resistance"[Title/Abstract]) OR "ETCO2 monitoring"[Title/Abstract]) OR "SpO2 waveforms"[Title/Abstract]) OR "central venous pressure"[Title/Abstract]) OR "central venous oxygen saturation"[Title/Abstract]) OR "mixed venous oxygen saturation"[Title/Abstract]) OR "airway pressure"[Title/Abstract]) OR "near-infrared spectroscopy"[Title/Abstract]) OR "arterial lines"[Title/Abstract])) AND survival[Title/Abstract]) AND ((((("neurological outcome"[Title/Abstract]) OR "functional outcome"[Title/Abstract]) OR "Hospital discharge"[Title/Abstract]) OR discharge[Title/Abstract]))

**Search equation EMBASE**

('cardiac arrest':ti,ab,kw OR 'cardiopulmonary arrest':ti,ab,kw OR 'circulatory arrest':ti,ab,kw OR 'heart arrest':ti,ab,kw) AND (cpr:ti,ab,kw OR 'systolic arterial pressure':ti,ab,kw OR 'diastolic arterial pressure':ti,ab,kw OR 'mean arterial pressure':ti,ab,kw OR 'systemic vascular resistance':ti,ab,kw OR 'etco2 monitoring':ti,ab,kw OR 'spo2 waveforms':ti,ab,kw OR 'central venous pressure':ti,ab,kw OR 'central venous oxygen saturation':ti,ab,kw OR 'mixed venous oxygen saturation':ti,ab,kw OR 'airway pressure':ti,ab,kw OR 'near-infrared spectroscopy':ti,ab,kw OR 'arterial lines':ti,ab,kw) AND survival:ti,ab,kw AND ('neurological outcome':ti,ab,kw OR 'functional outcome':ti,ab,kw OR 'hospital discharge':ti,ab,kw OR discharge:ti,ab,kw)

**Search equation Cochrane Library**

"cardiac arrest" OR "cardiopulmonary arrest" OR "circulatory arrest" OR "heart arrest" AND CPR OR "systolic arterial pressure" OR "diastolic arterial pressure" OR "mean arterial pressure" OR "systemic vascular resistance" OR "ETCO2 monitoring" OR "SpO2 waveforms" OR "central venous pressure" OR "central venous oxygen saturation" OR "mixed venous oxygen saturation" OR "airway pressure" OR "near-infrared spectroscopy" OR "arterial lines" AND survival AND "neurological outcome" OR "functional outcome" OR "Hospital discharge" OR discharge

**Study selection**

3144 records screened

3141 articles discarded (duplicates or excluded after title and abstract evaluation)

0 full-text article excluded due to absence of comparator

3 study included in qualitative synthesis

**Characteristics of included studies**

| Study | Design | Patients | Interventions | Outcomes |
| --- | --- | --- | --- | --- |
| Maillard et al. | Case Report / Observational | 1 | None / Oxygen Saturation Observation | Primary: Survival at discharge |
|  |  |  |  | Secondary: |
| Paarmann et al. | Case Report / Observational | 1 | None / Oxygen Saturation Observation | Primary: Survival at discharge |
|  |  |  |  | Secondary: |
| Yunoki et al. | Case Report / Observational | 1 | None / Oxygen Saturation Observation | Primary: Survival at discharge |
|  |  |  |  | Secondary: |

**Grade evidence table**

| **Outcomes** | **Quality assessment** | | | | | | | **Summary of findings** | | | | | | |
| --- | --- | --- | --- | --- | --- | --- | --- | --- | --- | --- | --- | --- | --- | --- |
|  |  |  |  |  |  |  |  | **No of patients** | | **Effect** | | | **Quality of evidence**  **(GRADE)** | |
|  | **No of participants**  **(studies)** | **Design** | **Risk of bias** | **Inconsistency** | **Indirectness** | **Imprecision** | **Other**  **considerations** | **Physiological feedback regarding CPR quality** | **No physiological feedback regarding CPR quality** | **Relative**  **(95% CI)** | **Risk**  **difference with physiological feedback regarding CPR quality** | **Absolute** |  |  |
| Survival - discharge | 1 (2) | Observational | Very serious^a^ | Not serious | Not serious | Not serious | None | 1/2 | - | - | - | - | ⊕🌕🌕🌕  Very low |  |
| Survival - discharge with good functional outcomes | 1 (1) | Observational | Very serious^b^ | Not serious | Not serious | Not serious | None | 1/1 (100.0%) | - | - | - | - | ⊕🌕🌕🌕  Very low |  |

a. Two studies are case reports

b. Case report. Two interventions at the same time (lateral position + blood pressure feedback)

**Consensus of science statement**

In adults with cardiac arrest in the perioperative setting, the use of physiological feedback may be reasonable to increase CPR quality and improve short- and long-term outcome (COR/LOE: IIb/C-EO).

**References**

1. Maillard J, Sologashvili T, Diaper J, Licker MJ, Keli Barcelos G. A Case of Persistence of Normal Tissue Oxygenation Monitored by Near-Infrared Spectroscopy (NIRS) Values Despite Prolonged Perioperative Cardiac Arrest. Am J Case Rep 2019;20:21-25.
2. Paarmann H, Heringlake M, Sier H, Schön J. The association of non-invasive cerebral and mixed venous oxygen saturation during cardiopulmonary resuscitation. Interact Cardiovasc Thorac Surg 2010;11:371-373.
3. Yunoki K, Sasaki R, Taguchi A, Maekawa S, Ueta H, Yamazaki K. Successful recovery without any neurological complication after intraoperative cardiopulmonary resuscitation for an extended period of time in the lateral position: a case report. JA Clin Rep 2016;2:7.

**PICO 3**

Among adults who are in ventricular fibrillation or pulseless ventricular tachycardia in the perioperative setting (P), does any interval of CPR first (e.g., 2 min) (I), compared with defibrillation first (C), change survival with favorable neurological/functional outcome at discharge, 30 days, 60 days, 180 days and/or 1 year, survival only at discharge, 30 days, 60days, 180 days and/or 1 year, ROSC, termination of arrhythmia (O)?

**Search equation PUBMED**

((((((((((((ventricular fibrillation) OR (pulseless ventricular tachycardia)) AND (perioperative)) AND (cpr)) OR (defibrillation)) NOT (animals)) NOT (pediatric)) NOT (children)) NOT (out-of-hospital))) NOT (implantable)) Filters: Clinical Trial

**Search equation EMBASE**

(('ventricular fibrillation':ti OR 'pulseless ventricular tachycardia':ti) AND defibrillation:ti OR cpr:ti) NOT pediatric:ti NOT animal:ti,ab,kw NOT 'out of hospital':ti,ab,kw NOT implantable:ti,ab,kw NOT 'perioperative period':ti,ab,kw NOT child:ti,ab,kw AND ([controlled clinical trial]/lim OR [randomized controlled trial]/lim) AND [1967-2020]/py

**Search equation Cochrane Library**

"Ventricular fibrillation" in Title Abstract Keyword OR "pulseless electrical activity" in Title Abstract Keyword AND "perioperative" in Record Title AND in Title Abstract Keyword AND "defibrillation" in Record Title

**Study selection**

796 records screened

779 articles discarded (duplicates or excluded after title and abstract evaluation)

12 full-text articles excluded due to absence of comparator

5 study included in qualitative synthesis

**Characteristics of included studies**

| Study | Design | Patients | Interventions | Outcomes |
| --- | --- | --- | --- | --- |
| Peberdy et al. | Prospective, multicenter, observational | 75 | Defibrillation - Time to first shock (< or > 3 min) | Primary: Survival to hospital discharge |
| Skogvoll and Nordseth | Observational | 219 | Defibrillation - BLS vs defibrillation | Primary: Survival |
| Chan et al. | Prospective, multicenter registry | 6789 | Defibrillation -Time to defibrillation early (<2 min), or delayed (>2 min) | Primary: Survival to hospital discharge |
|  |  |  |  | Secondary: ROSC (20 min after cardiac arrest); survival at 24 h; neurologic status at discharge |
| Chan et al. | Cohort study, National registry | 814 | AED use vs no AED | Primary: Survival to hospital discharge |
| Davis et al. | Retrospective, observational | 661 | Defibrillation - Stacked Shock (SS) vs Initial Chest Compression (ICC) vs Modified Stacked Shock (MSS) | Primary: Survival to hospital discharge |
|  |  |  |  | Secondary: ROSC |

**Grade evidence table**

| **Outcomes** | **Quality assessment** | | | | | | | **Summary of findings** | | | | | | |
| --- | --- | --- | --- | --- | --- | --- | --- | --- | --- | --- | --- | --- | --- | --- |
|  |  |  |  |  |  |  |  | **No of patients** | | **Effect** | | | **Quality of evidence**  **(GRADE)** | |
|  | **No of participants**  **(studies)** | **Design** | **Risk of bias** | **Inconsistency** | **Indirectness** | **Imprecision** | **Other**  **considerations** | **Interval of CPR** | **No interval of CPR** | **Relative**  **(95% CI)** | **Risk**  **difference with interval of CPR** | **Absolute** |  |  |
| Return of spontaneous circulation | 219 (2)  6789 (3)  75(5) | Prospective single center  Prospective, multicenter registry  Retrospective, observational | Very serious  Serious  Very serious | Not serious  Not serious  Serious | Not serious  Not serious  Serious | Serious  Serious  Serious | None  None  None | n/a  1003/2045  33/75 | n/a  3165/4744  42/75 | n/a  0.48 (0,43-0.53)  n/a | n/a  Not significant  Not significant | n/a  0.55  n/a | ⊕⊕🌕🌕  Low  ⊕⊕⊕🌕  Moderate  ⊕🌕🌕🌕  Very low |  |
| Survival to discharge | 1144 (1)  6789 (3)  2079 (4)  75(5) | Prospective, multicenter, observational  Prospective, multicenter registry  Prospective, multicenter, observational  Retrospective, observational | Very serious  Serious  Very serious  Very serious | Not serious  Not serious  Not serious  Serious | Not serious  Not serious  Not serious  Serious | Serious  Serious  Serious  Serious | None  None  None  None | 107/500  455/2045  368/804  33/75 | 1037/2714  1863/4744  286/593  42/75 | 2,16 (1.80-2.50)  0.44 (0.39-0.50)  1.05 (0.94-1.18)  n/a | Not significant  Not significant  Not significant  Significant | 0.42  0.48  1.03  n/a | ⊕⊕🌕🌕  Low  ⊕⊕⊕🌕  Moderate  ⊕⊕🌕🌕  Low  ⊕🌕🌕🌕  Very low |  |
| Survival at 30 days | - | - | - | - | - | - | - | - | - | - | - | - | - |  |
| Survival at 60 days | - | - | - | - | - | - | - | - | - | - | - | - | - |  |
| Survival at 180 days | - | - | - | - | - | - | - | - | - | - | - | - | - |  |
| Survival at 1 year | - | - | - | - | - | - | - | - | - | - | - | - | - |  |
| Survival to discharge with good neurologic outcome | 1587 (3) | Prospective, multicenter registry | Serious | Not serious | Not serious | Serious | None | 100/381 | 533/1542 | 0.67(0.52-0.87) | Not significant | 0.74 | ⊕⊕⊕🌕  Moderate |  |
| Survival at 30 days with good neurologic outcome | - | - | - | - | - | - | - | - | - | - | - | - | - |  |
| Survival at 60 days with good neurologic outcome | - | - | - | - | - | - | - | - | - | - | - | - | - |  |
| Survival at 180 days with good neurologic outcome | - | - | - | - | - | - | - | - | - | - | - | - | - |  |
| Survival at 1 year with good neurologic outcome | - | - | - | - | - | - | - | - | - | - | - | - | - |  |

**Consensus of science statement**

In adult patients with PERIOPCA, ventricular fibrillation/pulseless ventricular tachycardia should be defibrillated within 3 minutes after the onset of the arrest (COR/LOE: I/C-LD). The use of AEDs in patients with ventricular fibrillation/pulseless ventricular tachycardia can be useful for improving survival (COR/LOE: IIa/C-LD). It is not recommended to defibrillate patients with ventricular fibrillation/pulseless ventricular tachycardia lasting more than 3 minutes without prior chest compressions (COR/LOE: III/C-LD).

**References**

1. Peberdy MA, Kaye W, Ornato JP, Larkin GL, Nadkarni V, Mancini ME, Berg RA, Nichol G, Lane-Trultt T. Cardiopulmonary resuscitation of adults in the hospital: a report of 14720 cardiac arrests from the National Registry of Cardiopulmonary Resuscitation. Resuscitation 2003;58:297-308.
2. Skogvoll E, Nordseth T. The early minutes of in-hospital cardiac arrest: shock or CPR? A population based prospective study. Scand J Trauma Resusc Emerg Med 2008;16:11.
3. Chan PS, Krumholz HM, Nichol G, Nallamothu BK; American Heart Association National Registry of Cardiopulmonary Resuscitation Investigators. Delayed time to defibrillation after in-hospital cardiac arrest. N Engl J Med 2008;358:9-17.
4. Chan PS, Krumholz HM, Spertus JA, Jones PG, Cram P, Berg RA, Peberdy MA, Nadkarni V, Mancini ME, Nallamothu BK; American Heart Association National Registry of Cardiopulmonary Resuscitation (NRCPR) Investigators. Automated external defibrillators and survival after in-hospital cardiac arrest. JAMA 2010;304:2129-2136.
5. Davis D, Aguilar SA, Sell R, Minokadeh A, Husa R. A focused investigation of expedited, stack of three shocks versus chest compressions first followed by single shocks for monitored ventricular fibrillation/ventricular tachycardia cardiopulmonary arrest in an in-hospital setting. J Hosp Med 2016;11:264-268.

**PICO 4**

Among adults who are in cardiac arrest in the perioperative setting (P), does early epinephrine delivery by IV or IO route (e.g., less than 10 min after the beginning of resuscitation) (I), compared with delayed timing of epinephrine delivery (e.g., more than 10 min after the beginning of resuscitation) (C), change survival with favorable neurologic/functional outcome at discharge, 30 days, 60 days, 180 days, and/or 1 year; survival only at discharge, 30 days, 60 days, 180 days, and/or 1 year; ROSC (O)?

**Search equation PUBMED**

((((((((("cardiac arrest"[Title/Abstract]) OR "cardiopulmonary arrest"[Title/Abstract]) OR "circulatory arrest"[Title/Abstract]) OR "heart arrest"[Title/Abstract])) AND (((epinephrine[Title/Abstract]) OR adrenaline[Title/Abstract]))) AND ((((((((delayed[Title/Abstract]) OR early[Title/Abstract]) OR delivery[Title/Abstract]) OR "early delivery"[Title/Abstract]) OR "delayed delivery"[Title/Abstract]) OR time[Title/Abstract]) OR timing[Title/Abstract]))) AND survival[Title/Abstract]) AND ((((("neurological outcome"[Title/Abstract]) OR "functional outcome"[Title/Abstract]) OR "Hospital discharge"[Title/Abstract]) OR discharge[Title/Abstract])))

**Search equation EMBASE**

('cardiac arrest':ti,ab,kw OR 'cardiopulmonary arrest':ti,ab,kw OR 'circulatory arrest':ti,ab,kw OR 'heart arrest':ti,ab,kw) AND (epinephrine:ti,ab,kw OR adrenaline:ti,ab,kw) AND (delayed:ti,ab,kw OR early:ti,ab,kw OR delivery:ti,ab,kw OR 'early delivery':ti,ab,kw OR 'delayed delivery':ti,ab,kw OR time:ti,ab,kw OR timing:ti,ab,kw) AND survival:ti,ab,kw AND ('neurological outcome':ti,ab,kw OR 'functional outcome':ti,ab,kw OR 'hospital discharge':ti,ab,kw OR discharge:ti,ab,kw)

**Search equation Cochrane Library**

"cardiac arrest" OR "cardiopulmonary arrest" OR "circulatory arrest" OR "heart arrest" AND epinephrine OR adrenaline AND delayed OR early OR delivery OR "early delivery" OR "delayed delivery" OR time OR timing AND survival AND "neurological outcome" OR "functional outcome" OR "Hospital discharge" OR discharge

**Study selection**

348 records screened

338 articles discarded (duplicates or excluded after title and abstract evaluation)

8 full-text articles excluded due to absence of comparator

2 study included in qualitative synthesis

**Characteristics of included studies**

| Study | Design | Patients | Interventions | Outcomes |
| --- | --- | --- | --- | --- |
| Bircher et al. | Observational | 46310 | Epinephrine within five minutes after the commencement of CPR | Primary: Survival - discharge with good functional outcome |
|  |  |  |  | Secondary: Survival - Discharge |
| Andersen et al. | Observational | 2974 | Epinephrine within two minutes after the first  defibrillation | Primary: Return of spontaneous circulation |
|  |  |  |  | Secondary: Survival - discharge |

**Grade evidence table**

| **Outcomes** | **Quality assessment** | | | | | | | **Summary of findings** | | | | | |
| --- | --- | --- | --- | --- | --- | --- | --- | --- | --- | --- | --- | --- | --- |
|  |  |  |  |  |  |  |  | **No of patients** | | **Effect** | | | **Quality of evidence**  **(GRADE)** |
|  | **No of participants**  **(studies)** | **Design** | **Risk of bias** | **Inconsistency** | **Indirectness** | **Imprecision** | **Other**  **considerations** | **Early epinephrine delivery** | **Delayed epinephrine delivery** | **Relative**  **(95% CI)** | **Absolute** | **Risk**  **with early epinephrine delivery** |  |
| Survival - discharge with good functional outcome | 2834 (1) | Observational | Not serious | Not serious | Not serious | Not serious | None | 357/1445 | 567/1389 | RR 0.605  (0.542 - 0.676) | 247 per 1.000 (221 to 276) | 161 fewer per 1.000 (from 187 fewer to 132 fewer) | ⊕⊕🌕🌕  Low |
| Survival - discharge | 49284 (2) | Observational | Serious | Not serious | Not serious | Not serious | All plausible residual confounding would reduce the demonstrated effect | 4588/35272 | 2203/14012 | RR 0.827  (0.789 - 0.867) | 130 per 1.000 (124 to 136) | 27 fewer per 1.000 (from 33 fewer to 21 fewer) | ⊕⊕🌕🌕  Low |
| Return of spontaneous circulation | 2974 (1) | Observational | Not serios | Not serious | Not serious | Not serious | None | 1118/1510 | 1158/1464 | RR 0.936  (0.900 - 0.974) | 740 per 1.000 (712 to 770) | 51 fewer per 1.000 (from 79 fewer to 21 fewer) | ⊕⊕🌕🌕  Low |

**Consensus of science statement**

In adult patients with PERIOPCA, epinephrine administration after the 3^rd^ shock can be beneficial (COR/LOE: IIa/C-LD).


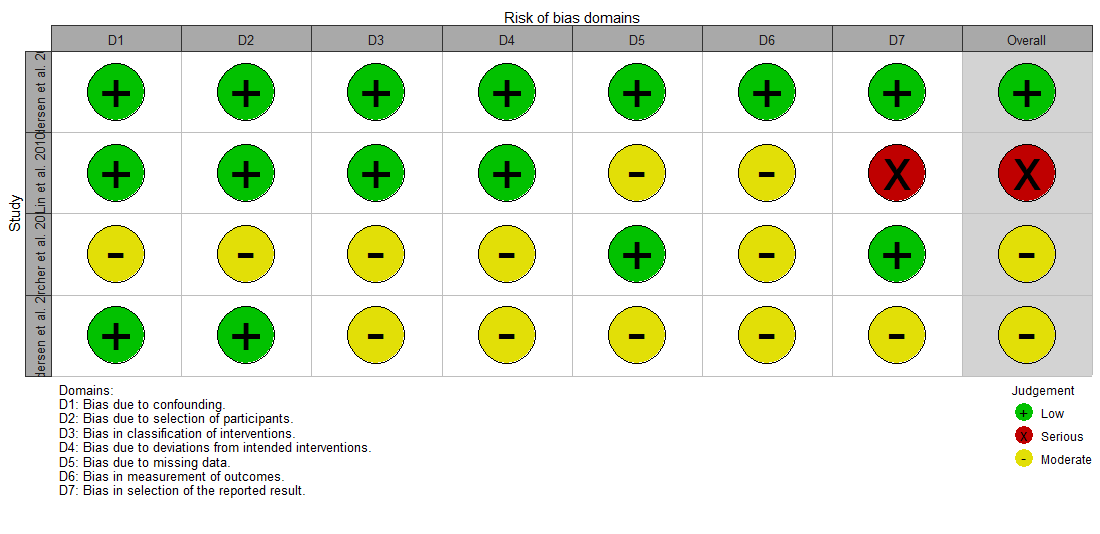


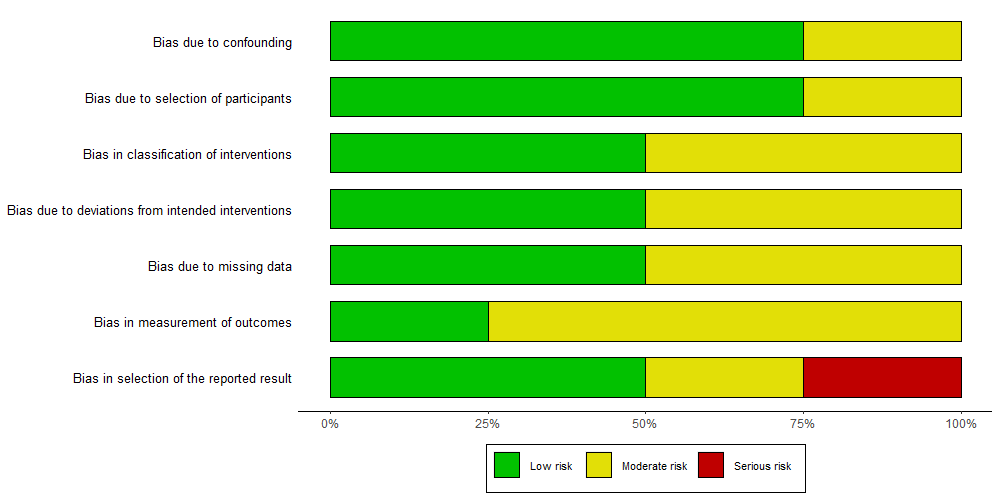


**References**

1. Bircher NG, Chan PS, Xu Y, American Heart Association's Get With The Guidelines-Resuscitation I. Delays in Cardiopulmonary Resuscitation, Defibrillation, and Epinephrine Administration All Decrease Survival in In-hospital Cardiac Arrest. Anesthesiology 2019;130:414-422.
2. Andersen LW, Kurth T, Chase M, Berg KM, Cocchi MN, Callaway C, Donnino MW; American Heart Association’s Get With The Guidelines-Resuscitation Investigators. Early administration of epinephrine (adrenaline) in patients with cardiac arrest with initial shockable rhythm in hospital: propensity score matched analysis. BMJ 2016;353:i1577.

**PICO 5**

In adult patients in cardiac arrest in the perioperative setting (P), does low-dose epinephrine (< 1 mg) or high-dose epinephrine (> 1mg) (I), compared with standard-dose epinephrine (1 mg bolus dose) (C), change survival to 180 days with good neurologic outcome, survival to 180 days, survival to hospital discharge with good neurologic outcome, survival to hospital discharge, ROSC (O)?

**Search equation PUBMED**

(((((((("cardiac arrest"[Title/Abstract]) OR "cardiopulmonary arrest"[Title/Abstract]) OR "circulatory arrest"[Title/Abstract]) OR "heart arrest"[Title/Abstract]))) AND (((((((((("High-dose EPI"[Title/Abstract]) OR "High dose EPI"[Title/Abstract]) OR "High-dose epinephrine"[Title/Abstract]) OR "High dose epinephrine"[Title/Abstract]) OR HDE[Title/Abstract]) OR "Low-dose EPI"[Title/Abstract]) OR "Low dose EPI"[Title/Abstract]) OR "Low-dose epinephrine"[Title/Abstract]) OR "Low dose epinephrine"[Title/Abstract]) OR LDE[Title/Abstract])) AND ((((("Standard-dose EPI"[Title/Abstract]) OR "Standard dose EPI"[Title/Abstract]) OR "Standard-dose epinephrine"[Title/Abstract]) OR "Standard dose epinephrine"[Title/Abstract]) OR SDE[Title/Abstract])) AND survival[Title/Abstract]

**Search equation EMBASE**

('cardiac arrest':ti,ab,kw OR 'cardiopulmonary arrest':ti,ab,kw OR 'circulatory arrest':ti,ab,kw OR 'heart arrest':ti,ab,kw) AND ('high-dose epi':ti,ab,kw OR 'high dose epi':ti,ab,kw OR 'high-dose epinephrine':ti,ab,kw OR 'high dose epinephrine':ti,ab,kw OR hde:ti,ab,kw OR 'low-dose epi':ti,ab,kw OR 'low dose epi':ti,ab,kw OR 'low-dose epinephrine':ti,ab,kw OR 'low dose epinephrine':ti,ab,kw OR lde:ti,ab,kw) AND ('standard-dose epi':ti,ab,kw OR 'standard dose epi':ti,ab,kw OR 'standard-dose epinephrine':ti,ab,kw OR 'standard dose epinephrine':ti,ab,kw OR sde:ti,ab,kw) AND survival:ti,ab,kw

**Search equation Cochrane Library**

"cardiac arrest" OR "cardiopulmonary arrest" OR "circulatory arrest" OR "heart arrest" AND "High-dose EPI" OR "High dose EPI" OR "High-dose epinephrine" OR "High dose epinephrine" OR HDE OR "Low-dose EPI" OR "Low dose EPI" OR "Low-dose epinephrine" OR "Low dose epinephrine" OR LDE AND "Standard-dose EPI" OR "Standard dose EPI" OR "Standard-dose epinephrine" OR "Standard dose epinephrine" OR SDE AND survival

**Study selection**

43 records screened

43 articles discarded (duplicates or excluded after title and abstract evaluation)

0 full-text article excluded due to absence of comparator

0 study included in qualitative synthesis

**Characteristics of included studies**

| Study | Design | Patients | Interventions | Outcomes |
| --- | --- | --- | --- | --- |
| - | - | - | - | Primary: - |
|  |  |  |  | Secondary: - |

**Grade evidence table**

| **Outcomes** | **Quality assessment** | | | | | | | **Summary of findings** | | | | | | |
| --- | --- | --- | --- | --- | --- | --- | --- | --- | --- | --- | --- | --- | --- | --- |
|  |  |  |  |  |  |  |  | **No of patients** | | **Effect** | | | **Quality of evidence**  **(GRADE)** | |
|  | **No of participants**  **(studies)** | **Design** | **Risk of bias** | **Inconsistency** | **Indirectness** | **Imprecision** | **Other**  **considerations** | **low-dose epinephrine (< 1 mg) or high-dose epinephrine (> 1mg)** | **standard-dose epinephrine (1 mg bolus dose)** | **Relative**  **(95% CI)** | **Risk**  **difference with low-dose epinephrine (< 1 mg) or high-dose epinephrine (> 1mg)** | **Absolute** |  |  |
| Survival to 180 days with good neurologic outcome, survival to 180 days, survival to hospital discharge with good neurologic outcome, survival to hospital discharge, ROSC | – | - | - | - | - | - | - | - | - | - | - | - | - |  |

**Consensus of science statement**

In patients with PERIOPCA, it may be reasonable to administer 1 mg epinephrine for improving coronary perfusion pressure (COR/LOE: IIb/C-EO).

**References**

None.

**PICO 6**

Among adults who are in cardiac arrest in in the perioperative setting (P), does avoiding the use of any vasopressor during CPR (I), compared with using epinephrine alone or vasopressin alone, or vasopressin in combination with epinephrine (C), change survival with favorable neurologic/functional outcome at discharge, 30 days, 60 days, 180 days, and/or 1 year; survival only at discharge, 30 days, 60 days, 180 days, and/or 1 year (O)?

**Search equation PUBMED**

((((((((("cardiac arrest"[Title/Abstract]) OR "cardiopulmonary arrest"[Title/Abstract]) OR "circulatory arrest"[Title/Abstract]) OR "heart arrest"[Title/Abstract]))) AND ((((((((((((((CPR[Title/Abstract]) OR "systolic arterial pressure"[Title/Abstract]) OR "diastolic arterial pressure"[Title/Abstract]) OR "mean arterial pressure"[Title/Abstract]) OR "systemic vascular resistance"[Title/Abstract]) OR "ETCO2 monitoring"[Title/Abstract]) OR "SpO2 waveforms"[Title/Abstract]) OR "central venous pressure"[Title/Abstract]) OR "central venous oxygen saturation"[Title/Abstract]) OR "mixed venous oxygen saturation"[Title/Abstract]) OR "airway pressure"[Title/Abstract]) OR "arterial lines"[Title/Abstract]))) AND vasopressor[Title/Abstract]) AND ((Epinephrine[Title/Abstract]) OR adrenaline[Title/Abstract])) OR vasopressin[Title/Abstract])) AND (((((((survival[Title/Abstract]) OR "neurological outcome"[Title/Abstract]) OR "functional outcome"[Title/Abstract]) OR "Hospital discharge"[Title/Abstract]) OR discharge[Title/Abstract]))

**Search equation EMBASE**

"('cardiac arrest':ti,ab,kw OR 'cardiopulmonary arrest':ti,ab,kw OR 'circulatory arrest':ti,ab,kw OR 'heart arrest':ti,ab,kw) AND (cpr:ti,ab,kw OR 'systolic arterial pressure':ti,ab,kw OR 'diastolic arterial pressure':ti,ab,kw OR 'mean arterial pressure':ti,ab,kw OR 'systemic vascular resistance':ti,ab,kw OR 'etco2 monitoring':ti,ab,kw OR 'spo2 waveforms':ti,ab,kw OR 'central venous pressure':ti,ab,kw OR 'central venous oxygen saturation':ti,ab,kw OR 'mixed venous oxygen saturation':ti,ab,kw OR 'airway pressure':ti,ab,kw OR 'arterial lines':ti,ab,kw) AND vasopressor:ti,ab,kw AND (epinephrine:ti,ab,kw OR adrenaline:ti,ab,kw OR vasopressin:ti,ab,kw) AND (survival:ti,ab,kw OR 'neurological outcome':ti,ab,kw OR 'functional outcome':ti,ab,kw OR 'hospital discharge':ti,ab,kw OR discharge:ti,ab,kw)"

**Search equation Cochrane Library**

“cardiac arrest” OR “heart arrest” in Title Abstract Keyword AND “perioperative” OR “operating room” OR “intraoperative” in Title Abstract Keyword AND “no epinephrine” OR “no vasopressor” OR “epinephrine” OR “vasopressin” OR “epinephrine” and “vasopressin” in Title Abstract Keyword - (Word variations have been searched)

**Study selection**

85 records screened

85 articles discarded (duplicates or excluded after title and abstract evaluation)

0 full-text article excluded due to absence of comparator

0 study included in qualitative synthesis

**Characteristics of included studies**

| Study | Design | Patients | Interventions | Outcomes |
| --- | --- | --- | --- | --- |
| - | - | - | - | Primary: - |
|  |  |  |  | Secondary: - |

**Grade evidence table**

| **Outcomes** | **Quality assessment** | | | | | | | **Summary of findings** | | | | | | |
| --- | --- | --- | --- | --- | --- | --- | --- | --- | --- | --- | --- | --- | --- | --- |
|  |  |  |  |  |  |  |  | **No of patients** | | **Effect** | | | **Quality of evidence**  **(GRADE)** | |
|  | **No of participants**  **(studies)** | **Design** | **Risk of bias** | **Inconsistency** | **Indirectness** | **Imprecision** | **Other**  **considerations** | **No vasopressor during CPR** | **Epinephrine alone or vasopressin alone, or vasopressin in combination with epinephrine** | **Relative**  **(95% CI)** | **Risk**  **difference with no vasopressor during CPR** | **Absolute** |  |  |
| Survival with favorable neurologic/functional outcome at discharge, 30 days, 60 days, 180 days, and/or 1 year; survival only at discharge, 30 days, 60 days, 180 days, and/or 1 year | – | - | - | - | - | - | - | - | - | - | - | - | - |  |

**Consensus of science statement**

In patients with PERIOPCA, it may be reasonable to administer epinephrine every 3 to 5 minutes (COR/LOE: IIb/C-EO).

**References**

None.

**PICO 7**

Among adults who are in cardiac arrest in the perioperative setting (P), does administration of antiarrhythmic drugs (e.g., amiodarone, lidocaine, other) (I), compared with not using antiarrhythmic drugs (no drug or placebo) (C), change survival with favorable neurologic/functional outcome at discharge, 30 days, 60 days, 180 days, and/or 1 year; survival only at discharge, 30 days, 60 days, 180 days, and/or 1 year; ROSC (O)?

**Search equation PUBMED**

(((((cardiac arrest) AND (antiarrhythmic)) NOT (out-of-hospital)) NOT (pediatric)) NOT (animals)) NOT (implantable)

**Search equation EMBASE**

(('cardiac arrest'/exp OR 'cardiac arrest' OR (cardiac AND ('arrest'/exp OR arrest))) AND antiarrhythmic:ti,ab,kw OR amiodarone:ti,ab,kw OR lidocaine:ti,ab,kw OR magnesium:ab,ti) NOT 'out of hospital':ab,ti NOT pediatric:ti,ab,kw NOT ('animal'/exp OR animal) NOT implantable:ti,ab,kw AND ('clinical trial'/exp OR 'clinical trial')

**Search equation Cochrane Library**

"cardiac-arrest" in Title Abstract Keyword AND "antiarrhythmic drug" in Title Abstract Keyword NOT "out of hospital" in Title Abstract Keyword NOT pediatric in Title Abstract Keyword NOT animals in Title Abstract Keyword

**Study selection**

228 records screened

221 articles discarded (duplicates or excluded after title and abstract evaluation)

3 full-text articles excluded due to absence of comparator

4 study included in qualitative synthesis

**Characteristics of included studies**

| Study | Design | Patients | Interventions | Outcomes |
| --- | --- | --- | --- | --- |
| Thel et al. | Prospective, randomized, single centre | 156 | Magnesium vs. placebo | Primary: ROSC |
|  |  |  |  | Secondary: Survival to 24h; Survival to hospital discharge |
| Pollak et al. | Retrospective | 374 | Amiodarone vs. lidocaine | Primary: ROSC |
|  |  |  |  | Secondary: Survival to discharge |
| Rea et al. | Retrospective multicenter | 194 | Amiodarone vs. lidocaine or combination of amiodarone and lidocaine | Primary: Survival to 24h |
|  |  |  |  | Secondary: Survival to discharge |
| Shiga et al. | Prospective, two-arm, observational | 55 | Nifekalant vs. lidocaine | Primary: Termination without shock; Termination with/without shock |
|  |  |  |  | Secondary: ROSC; 1-month survival; survival to discharge |

**Grade evidence table**

| **Outcomes** | **Quality assessment** | | | | | | | **Summary of findings** | | | | | |
| --- | --- | --- | --- | --- | --- | --- | --- | --- | --- | --- | --- | --- | --- |
|  |  |  |  |  |  |  |  | **No of patients** | | **Effect** | | | **Quality of evidence**  **(GRADE)** |
|  | **No of participants**  **(studies)** | **Design** | **Limitations** | **Inconsistency** | **Indirectness** | **Imprecision** | **Other**  **considerations** | **Antiarrhythmic drugs** | **No antiarrhythmic drugs** | **Relative**  **(95% CI)** | **Risk**  **difference with antiarrhythmic drugs** | **Absolute** |  |
| Return of spontaneous circulation | 156 (1) | Prospective, randomized, single centre | Low | Not serious | Not serious | Not serious | None | 41/156 | 48/156 | 0·78 (0·41–1·47) | Not significant | 0.49 | ⊕⊕⊕🌕  Moderate |
| Survival to discharge | 156 (1) | Prospective, randomized, single centre | Low | Not serious | Not serious | Not serious | None | 16/156 | 17/156 | 0·99 (0·46–2·13) | Not significant | 0.10 | ⊕⊕⊕🌕  Moderate |
| Survival at 30 days | - | - | - | - | - | - | - | - | - | - | - | - | - |
| Survival at 60 days | - | - | - | - | - | - | - | - | - | - | - | - | - |
| Survival at 180 days | - | - | - | - | - | - | - | - | - | - | - | - | - |
| Survival at 1 year | - | - | - | - | - | - | - | - | - | - | - | - | - |
| Survival to discharge with good neurologic outcome | - | - | - | - | - | - | - | - | - | - | - | - | - |
| Survival at 30 days with good neurologic outcome | - | - | - | - | - | - | - | - | - | - | - | - | - |
| Survival at 60 days with good neurologic outcome | - | - | - | - | - | - | - | - | - | - | - | - | - |
| Survival at 180 days with good neurologic outcome | - | - | - | - | - | - | - | - | - | - | - | - | - |
| Survival at 1 year with good neurologic outcome | - | - | - | - | - | - | - | - | - | - | - | - | - |

**Consensus of science statement**

In adult patients with PERIOPCA, it is recommended to administer amiodarone or lidocaine for the treatment of ventricular fibrillation/pulseless ventricular tachycardia (COR/LOE: I/C-LD). Magnesium is not indicated for the treatment of ventricular fibrillation/pulseless ventricular tachycardia in the perioperative setting (COR/LOE: III/C-LD).

**References**

1. Thel MC, Armstrong AL, McNulty SE, Califf RM, O'Connor CM. Randomised trial of magnesium in in-hospital cardiac arrest. Duke Internal Medicine Housestaff. Lancet 1997;350:1272-1276.
2. Pollak PT, Wee V, Al-Hazmi A, Martin J, Zarnke KB. The use of amiodarone for in-hospital cardiac arrest at two tertiary care centres. Can J Cardiol 2006;22:199-202.
3. Rea RS, Kane-Gill SL, Rudis MI, Seybert AL, Oyen LJ, Ou NN, Stauss JL, Kirisci L, Idrees U, Henderson SO. Comparing intravenous amiodarone or lidocaine, or both, outcomes for inpatients with pulseless ventricular arrhythmias. Crit Care Med 2006;34:1617-1623.
4. Shiga T, Tanaka K, Kato R, Amino M, Matsudo Y, Honda T, Sagara K, Takahashi A, Katoh T, Urashima M, Ogawa S, Takano T, Kasanuki H; Refractory VT/VF, Prospective Evaluation to Differentiate Lidocaine Efficacy from Nifekalant (RELIEF) Study Investigators. Nifekalant versus lidocaine for in-hospital shock-resistant ventricular fibrillation or tachycardia. Resuscitation 2010;81:47-52.

**PICO 8**

Among adults who are in cardiac arrest in the perioperative setting (P), does early antiarrhythmic (e.g., amiodarone, lidocaine, other) delivery by IV or IO route (e.g., less than 6 min after the beginning of resuscitation) (I), compared with delayed timing of antiarrhythmic delivery (e.g., more than 6 min after the beginning of resuscitation) (C), change survival with favorable neurologic/functional outcome at discharge, 30 days, 60 days, 180 days, and/or 1 year; survival only at discharge, 30 days, 60 days, 180 days, and/or 1 year; ROSC (O)?

**Search equation PUBMED**

(((((cardiac arrest) AND (antiarrhythmic)) NOT (out-of-hospital)) NOT (pediatric)) NOT (animals)) NOT (implantable)

**Search equation EMBASE**

(('cardiac arrest'/exp OR 'cardiac arrest' OR (cardiac AND ('arrest'/exp OR arrest))) AND antiarrhythmic:ti,ab,kw OR amiodarone:ti,ab,kw OR lidocaine:ti,ab,kw OR magnesium:ab,ti) NOT 'out of hospital':ab,ti NOT pediatric:ti,ab,kw NOT ('animal'/exp OR animal) NOT implantable:ti,ab,kw AND ('clinical trial'/exp OR 'clinical trial')

**Search equation COCHRANE LIBRARY**

"cardiac-arrest" in Title Abstract Keyword AND "antiarrhythmic drug" in Title Abstract Keyword NOT "out of hospital" in Title Abstract Keyword NOT pediatric in Title Abstract Keyword NOT animals in Title Abstract Keyword

**Study selection**

228 records screened

228 articles discarded (duplicates or excluded after title and abstract evaluation)

0 full-text article excluded due to absence of comparator

0 study included in qualitative synthesis

**Characteristics of included studies**

| Study | Design | Patients | Interventions | Outcomes |
| --- | --- | --- | --- | --- |
| - | - | - |  | Primary: - |
|  |  |  |  | Secondary: - |

**Grade evidence table**

| **Outcomes** | **Quality assessment** | | | | | | | **Summary of findings** | | | | | |
| --- | --- | --- | --- | --- | --- | --- | --- | --- | --- | --- | --- | --- | --- |
|  |  |  |  |  |  |  |  | **No of patients** | | **Effect** | | | **Quality of evidence**  **(GRADE)** |
|  | **No of participants**  **(studies)** | **Design** | **Risk of bias** | **Inconsistency** | **Indirectness** | **Imprecision** | **Other**  **considerations** | **Early antiarrhythmic delivery** | **Delayed antiarrhythmic delivery** | **Relative**  **(95% CI)** | **Risk**  **difference with early antiarrhythmic delivery** | **Absolute** |  |
| Survival with favorable neurologic/functional outcome at discharge, 30 days, 60 days, 180 days, and/or 1 year; survival only at discharge, 30 days, 60 days, 180 days, and/or 1 year; ROSC | – | - | - | - | - | - | - | - | - | - | - | - | - |

**Consensus of science statement**

In adult patients with perioperative ventricular fibrillation/pulseless ventricular tachycardia, it might be reasonable to administer amiodarone or lidocaine after the 3^rd^ shock (COR/LOE: IIb/C-EO).

**References**

None.

**PICO 9**

Among adults with cardiac arrest with a secure airway receiving chest compressions (in the perioperative setting, and with standard tidal volume) (P), does a ventilation rate of 10 breaths/min (I), compared with any other ventilation rate (C), change survival with favorable neurologic/functional outcome at discharge, 30 days, 60 days, 180 days, and/or 1 year; survival only at discharge, 30 days, 60 days, 180 days, and/or 1 year; ROSC (O)?

**Search equation PUBMED**

("Heart Arrest"[Mesh:NoExp]) OR "Cardiopulmonary Resuscitation"[Mesh:NoExp] OR “Cardiac arrest” [Title/Abstract] OR “Cardiac arrests” [Title/Abstract] OR “Heart Arrest” [Title/Abstract] OR “Heart Arrests” [Title/Abstract] OR “cardiopulmonary arrest"[Title/Abstract] OR “cardiopulmonary arrests”[Title/Abstract] OR "Cardiopulmonary resuscitation"[Title/Abstract] OR “Cardio Pulmonary resuscitation” [Title/Abstract]) AND (“Secure airway” [Title/Abstract] OR “Secure airways” [Title/Abstract] OR “laryngeal mask” [Title/Abstract] OR “laryngeal masks” [Title/Abstract] OR "Laryngeal Masks"[Mesh] OR “tracheal intubation” [Title/Abstract] OR “tracheal intubated” [Title/Abstract] OR "Intubation, Intratracheal"[Mesh] OR “tracheal tube” [Title/Abstract] OR “Ventilation rate” [Title/Abstract]) AND ("mortality"[Subheading] OR "mortality” [Title/Abstract] OR survival[Title/Abstract] OR "survival"[MeSH Terms] OR “return spontaneous circulation” [Title/Abstract] OR resuscitation[Title/Abstract] OR resuscitated[Title/Abstract] OR "Resuscitation"[Mesh] OR “Cerebral performance category” [Title/Abstract] OR "cerebral performance categories"[Title/Abstract] OR “Modified ranking” [Title/Abstract]) NOT (("Out-of-Hospital Cardiac Arrest"[Mesh]) OR "out of hospital") OR "out of hospitals". Filters: English; Greek, Modern; Italian; Adult: 19+ years OR (adult OR adults).

**Search equation EMBASE**

**#1** 'heart arrest'/de OR 'cardiopulmonary arrest'/de OR 'cardiac arrest*':ti,ab OR 'heart arrest*':ti,ab OR 'cardiopulmonary arrest*':ti,ab OR 'cardiopulmonary resuscitation':ti,ab OR 'cardio pulmonary resuscitation':ti,ab

**#2** 'secure airway*':ti,ab

**#3** 'laryngeal mask*':ti,ab

**#4** 'laryngeal mask'/exp

**#5** 'tracheal intubated':ti,ab OR 'endotracheal intubation'/exp OR 'tracheal tube':ti,ab OR 'ventilation rate':ti,ab OR 'tracheal intubation':ti,ab

**#6** #2 OR #3 OR #4 OR #5

**#7** 'mortality'/exp OR mortality:ti,ab OR survival:ti,ab OR 'survival'/exp OR 'return spontaneous circulation':ti,ab OR resuscitation:ti,ab OR resuscitated:ti,ab OR 'resuscitation'/exp OR 'cerebral performance category':ti,ab OR 'cerebral performance categories':ti,ab OR 'modified ranking':ti,ab

**#8** #1 AND #6 AND #7

**#9** 'out of hospital cardiac arrest'/exp OR 'out of hospital' OR 'out of hospitals'

**#10** #8 NOT #9

**#11** #8 NOT #9 AND ([english]/lim OR [greek]/lim OR [italian]/lim)

**#12** #11 AND ([adult]/lim OR [aged]/lim OR [middle aged]/lim OR [very elderly]/lim OR [young adult]/lim)

**#13** #11 AND ('adult' OR adults)

**#14** #12 OR #13

**#15** [embase]/lim NOT [medline]/lim

**#16** #14 AND #15

**Search equation Cochrane Library**

**#1** “Cardiac arrest” OR “Cardiac arrests” OR “Heart Arrest” OR “Heart Arrests” OR “cardiopulmonary arrest " OR “cardiopulmonary arrests "

**#2** "Cardiopulmonary resuscitation " OR “Cardio Pulmonary resuscitation”

**#3** MeSH descriptor: [Heart Arrest] explode all trees

**#4** MeSH descriptor: [Cardiopulmonary Resuscitation] this term only

**#5** #1 or #2 or #3 or #4

**#6** “Secure airway” OR “Secure airways” OR “laryngeal mask” OR “laryngeal masks”

**#7** “tracheal intubation” OR “tracheal intubated” OR “tracheal tube*” OR “Ventilation rate

**#8** MeSH descriptor: [Laryngeal Masks] explode all trees

**#9** MeSH descriptor: [Resuscitation] explode all trees

**#10** #6 or #7 or #8 or #9

**#11** MeSH descriptor: [Mortality] explode all trees

**#12** MeSH descriptor: [Survival] explode all trees

**#13** mortality or survival

**#14** #11 or #12 or #13

**#15** “Cerebral performance category” OR "cerebral performance categories" OR “Modified ranking”

**#16** #14 or #15

**#17** #5 and #10 and #16

**#18** MeSH descriptor: [Out-of-Hospital Cardiac Arrest] explode all trees

**#19** "out of hospital*"

**#20** #18 or #19

**#21** #17 not #20

**#22** #21 and (english OR italian OR greek)

**#23** adult Or adults

**#24** MeSH descriptor: [Adult] explode all trees

**#25** #23 or #24

**#26** #22 and #25

**#27** "accession number" near pubmed

**#28** "accession number" near2 embase

**#29** #27 or #28

**#30** #26 not #29

**Study selection**

256 records screened

256 articles discarded (duplicates or excluded after title and abstract evaluation)

0 full-text article excluded due to absence of comparator

0 study included in qualitative synthesis

**Characteristics of included studies**

| Study | Design | Patients | Interventions | Outcomes |
| --- | --- | --- | --- | --- |
| - | - | - |  | Primary: - |
|  |  |  |  | Secondary: - |

**Grade evidence table**

| **Outcomes** | **Quality assessment** | | | | | | | **Summary of findings** | | | | | | |
| --- | --- | --- | --- | --- | --- | --- | --- | --- | --- | --- | --- | --- | --- | --- |
|  |  |  |  |  |  |  |  | **No of patients** | | **Effect** | | | **Quality of evidence**  **(GRADE)** | |
|  | **No of participants**  **(studies)** | **Design** | **Risk of bias** | **Inconsistency** | **Indirectness** | **Imprecision** | **Other**  **considerations** | **Ventilation rate of 10 breaths/min** | **Any other ventilation rate** | **Relative**  **(95% CI)** | **Risk**  **difference with ventilation rate of 10 breaths/min** | **Absolute** |  |  |
| Survival with favorable neurologic/functional outcome at discharge, 30 days, 60 days, 180 days, and/or 1 year; survival only at discharge, 30 days, 60 days, 180 days, and/or 1 year; ROSC | – | - | - | - | - | - | - | - | - | - | - | - | - |  |

**Consensus of science statement**

In adult patients with PERIOPCA and a secure airway, a ventilation rate of 10 breaths/min during CPR may be reasonable (COR/LOE: IIb/C-EO).

**References**

None.

**PICO 10**

Among adults who are in cardiac arrest due to pulmonary embolism or suspected pulmonary embolism in the perioperative setting (P), does any specific alteration in treatment algorithm (e.g., fibrinolytics, or any other) (I), compared with standard care (C), change survival with favorable neurologic/functional outcome at discharge, 30 days, 60 days, 180 days, and/or 1 year; survival only at discharge, 30 days, 60 days, 180 days, and/or 1 year; ROSC (O)?

**Search equation PUBMED**

"Heart Arrest"[Mesh:NoExp] OR "Cardiopulmonary Resuscitation"[Mesh:NoExp] OR “Cardiac arrest” [Title/Abstract] OR “Cardiac arrests” [Title/Abstract] OR “Heart Arrest” [Title/Abstract] OR “Heart Arrests” [Title/Abstract] OR “cardiopulmonary arrest"[Title/Abstract] OR “cardiopulmonary arrests”[Title/Abstract] OR "Cardiopulmonary resuscitation"[Title/Abstract] OR “Cardio Pulmonary resuscitation” [Title/Abstract]AND (((“Pulmonary embolism”[Title/Abstract] OR “Pulmonary embolisms”[Title/Abstract] OR "Pulmonary Embolism"[Mesh] OR trombolysis[Title/Abstract] OR "Thrombolytic Therapy"[Mesh] OR streptokinase[Title/Abstract] OR "Streptokinase"[Mesh] OR urokinase[Title/Abstract] OR "Urokinase-Type Plasminogen Activator"[Mesh] OR ”recombinant tissue plasminogen activator”[Title/Abstract] OR "Tissue Plasminogen Activator"[Mesh] OR “surgical embolectomy”[Title/Abstract] OR “mechanical thrombectomy”[Title/Abstract])) OR (("Mechanical Thrombolysis"[Mesh]) AND "Thrombectomy"[Mesh])) OR (("Pulmonary Embolism"[Mesh]) AND "Thrombectomy"[Mesh])AND("mortality"[Subheading] OR "mortality” [Title/Abstract] OR survival[Title/Abstract] OR "survival"[MeSH Terms] OR “return spontaneous circulation” [Title/Abstract] OR resuscitation[Title/Abstract] OR resuscitated[Title/Abstract] OR "Resuscitation"[Mesh] OR “Cerebral performance category” [Title/Abstract] OR "cerebral performance categories"[Title/Abstract] OR “Modified ranking” [Title/Abstract])NOT"Out-of-Hospital Cardiac Arrest"[Mesh] OR “out of hospital”.

**Search equation EMBASE**

'heart arrest'/de OR 'cardiopulmonary arrest'/de OR 'cardiac arrest*':ti,ab OR 'heart arrest*':ti,ab OR 'cardiopulmonary arrest*':ti,ab OR 'cardiopulmonary resuscitation':ti,ab OR 'cardio pulmonary resuscitation':ti,ab AND 'mortality'/exp OR mortality:ti,ab OR survival:ti,ab OR 'survival'/exp OR 'return spontaneous circulation':ti,ab OR resuscitation:ti,ab OR resuscitated:ti,ab OR 'resuscitation'/exp OR 'cerebral performance category':ti,ab OR 'cerebral performance categories':ti,ab OR 'modified ranking':ti,ab AND 'pulmonary embolism':ti,ab OR 'pulmonary embolisms':ti,ab OR 'lung embolism'/exp OR trombolysis:ti,ab OR 'fibrinolytic therapy'/exp OR streptokinase:ti,ab OR 'streptokinase'/exp OR urokinase:ti,ab OR 'prourokinase'/exp OR 'recombinant tissue plasminogen activator':ti,ab OR 'tissue plasminogen activator'/exp OR 'surgical embolectomy':ti,ab OR 'mechanical thrombectomy':ti,ab OR 'mechanical thrombectomy'/exp OR 'pulmonary thrombectomy' NOT 'out of hospital cardiac arrest'/exp OR 'out of hospital' OR 'out of hospitals' AND ([english]/lim OR [greek]/lim OR [italian]/lim) AND ([adult]/lim OR [aged]/lim OR [middle aged]/lim OR [very elderly]/lim OR [young adult]/lim)

**Search equation Cochrane Library**

“Cardiac arrest” OR “Cardiac arrests” OR “Heart Arrest” OR “Heart Arrests” OR “cardiopulmonary arrest" OR “cardiopulmonary arrests" OR "Cardiopulmonary resuscitation " OR “Cardio Pulmonary resuscitation” in Title Abstract Keyword AND “pulmonary embolism” OR “pulmonary embolisms” OR “thrombolysis” OR “streptokinase” OR “urokinase” OR “recombinant tissue plasminogen activator” OR “surgical embolectomy” OR “mechanical thrombectomy” OR “pulmonary thrombectomy”' in Title Abstract Keyword in Title Abstract Keyword - (Word variations have been searched)

**Study selection**

1136 records screened

1136 articles discarded (duplicates or excluded after title and abstract evaluation)

0 full-text article excluded due to absence of comparator

0 study included in qualitative synthesis

**Characteristics of included studies**

| Study | Design | Patients | Interventions | Outcomes |
| --- | --- | --- | --- | --- |
| - | - | - |  | Primary: - |
|  |  |  |  | Secondary: - |

**Grade evidence table**

| **Outcomes** | **Quality assessment** | | | | | | | **Summary of findings** | | | | | | |
| --- | --- | --- | --- | --- | --- | --- | --- | --- | --- | --- | --- | --- | --- | --- |
|  |  |  |  |  |  |  |  | **No of patients** | | **Effect** | | | **Quality of evidence**  **(GRADE)** | |
|  | **No of participants**  **(studies)** | **Design** | **Risk of bias** | **Inconsistency** | **Indirectness** | **Imprecision** | **Other**  **considerations** | **Any specific alteration in treatment algorithm** | **Standard care** | **Relative**  **(95% CI)** | **Risk**  **difference with any specific alteration in treatment algorithm** | **Absolute** |  |  |
| Survival with favorable neurologic/functional outcome at discharge, 30 days, 60 days, 180 days, and/or 1 year; survival only at discharge, 30 days, 60 days, 180 days, and/or 1 year; ROSC | – | - | - | - | - | - | - | - | - | - | - | - | - |  |

**Consensus of science statement**

In adult patients with PERIOPCA due to pulmonary embolism or suspected pulmonary embolism, early consideration of thrombolysis and CPR duration of at least 60-90 minutes with or without the use of a mechanical chest compression device may be reasonable before terminating resuscitation attempts (COR/LOE: IIb/C-LD). The emergency treatment option among fibrinolytic therapy, surgical, or mechanical thrombectomy should be selected based on timing and available expertise, since no clear benefit of one approach over the other has been demonstrated.

**References**

None.

**PICO 11**

Among pregnant women who are in cardiac arrest in the perioperative setting (P), do any specific interventions (I), compared with standard care (usual resuscitation practice) (C), change survival with favorable neurologic/functional outcome at discharge, 30 days, 60 days, 180 days, and/or 1 year; survival only at discharge, 30 days, 60 days, 180 days, and/or 1 year; ROSC (O)?

**Search equation PUBMED**

"Heart Arrest"[Mesh:NoExp] OR "Cardiopulmonary Resuscitation"[Mesh:NoExp] OR “Cardiac arrest” [Title/Abstract] OR “Cardiac arrests” [Title/Abstract] OR “Heart Arrest” [Title/Abstract] OR “Heart Arrests” [Title/Abstract] OR “cardiopulmonary arrest"[Title/Abstract] OR “cardiopulmonary arrests”[Title/Abstract] OR "Cardiopulmonary resuscitation"[Title/Abstract] OR “Cardio Pulmonary resuscitation” [Title/Abstract] AND (((“pregnancy”[Title/Abstract] OR “pregnant”[Title/Abstract] OR "birth"[Mesh] AND ("mortality"[Subheading] OR "mortality” [Title/Abstract] OR survival[Title/Abstract] OR "survival"[MeSH Terms] OR “return spontaneous circulation” [Title/Abstract] OR resuscitation[Title/Abstract] OR resuscitated[Title/Abstract] OR "Resuscitation"[Mesh] OR “Cerebral performance category” [Title/Abstract] OR "cerebral performance categories"[Title/Abstract] NOT "Out-of-Hospital Cardiac Arrest"[Mesh] OR “out of hospital”.

**Search equation EMBASE**

'heart arrest'/de OR 'cardiopulmonary arrest'/de OR 'cardiac arrest*':ti,ab OR 'heart arrest*':ti,ab OR 'cardiopulmonary arrest*':ti,ab OR 'cardiopulmonary resuscitation':ti,ab OR 'cardio pulmonary resuscitation':ti,ab AND 'mortality'/exp OR mortality:ti,ab OR survival:ti,ab OR 'survival'/exp OR 'return spontaneous circulation':ti,ab OR resuscitation:ti,ab OR resuscitated:ti,ab OR 'resuscitation'/exp AND 'pregnancy':ti,ab OR 'pregnant':ti,ab OR 'birth'/exp NOT 'out of hospital cardiac arrest'/exp OR 'out of hospital' OR 'out of hospitals'

**Search equation Cochrane Library**

“Cardiac arrest” OR “Cardiac arrests” OR “Heart Arrest” OR “Heart Arrests” OR “cardiopulmonary arrest" OR “cardiopulmonary arrests" OR "Cardiopulmonary resuscitation" OR “Cardio Pulmonary resuscitation” in Title Abstract Keyword AND “pregnancy” OR “pregnant” OR “birth” in Title Abstract Keyword AND “survival” OR "return spontaneous circulation" in Title Abstract Keyword - (Word variations have been searched)

**Study selection**

198 records screened

198 articles discarded (duplicates or excluded after title and abstract evaluation)

0 full-text article excluded due to absence of comparator

0 studies included in qualitative synthesis

**Characteristics of included studies**

| Study | Design | Patients | Interventions | Outcomes |
| --- | --- | --- | --- | --- |
| - | - | - | - | Primary: - |
|  |  |  |  | Secondary: - |

**Grade evidence table**

| **Outcomes** | **Quality assessment** | | | | | | | **Summary of findings** | | | | | | |
| --- | --- | --- | --- | --- | --- | --- | --- | --- | --- | --- | --- | --- | --- | --- |
|  |  |  |  |  |  |  |  | **No of patients** | | **Effect** | | | **Quality of evidence**  **(GRADE)** | |
|  | **No of participants**  **(studies)** | **Design** | **Risk of bias** | **Inconsistency** | **Indirectness** | **Imprecision** | **Other**  **considerations** | **Any specific alteration in treatment algorithm** | **Standard care** | **Relative**  **(95% CI)** | **Risk**  **difference with any specific alteration in treatment algorithm** | **Absolute** |  |  |
| Survival with favorable neurologic/functional outcome at discharge, 30 days, 60 days, 180 days, and/or 1 year; survival only at discharge, 30 days, 60 days, 180 days, and/or 1 year; ROSC | – | - | - | - | - | - | - | - | - | - | - | - | - |  |

**Consensus of science statement**

In pregnant women with PERIOPCA, the effectiveness of any special interventions, compared to standard measures, is uncertain, except probably for manual uterine displacement during chest compressions (COR/LOE: IIb/C-EO). In pregnant women with PERIOPCA due to suspected or proven pulmonary embolism, it may be reasonable to use thrombolysis or other measures to remove clot (e.g., surgical or percutaneous pulmonary embolectomy) (COR/LOE: IIb/C-EO). Extracorporeal membrane oxygenation may be considered as an acceptable salvage therapy for pregnant and postpartum patients with PERIOPCA or those with critical cardiac or pulmonary illness (COR/LOE: IIb/C-EO).

**References**

None.

**PICO 12**

Among adults who are in cardiac arrest or respiratory arrest due to opioid toxicity in the perioperative setting (P), does any specific therapy (e.g., naloxone, bicarbonate, or other drugs) (I), compared with usual ALS (C), change survival with favorable neurologic/functional outcome at discharge, 30 days, 60 days, 180 days, and/or 1 year; survival only at discharge, 30 days, 60 days, 180 days, and/or 1 year; ROSC (O)?

**Search equation PUBMED**

(Heart Arrest[MeSH Terms] OR Cardiopulmonary Arrest[MeSH Terms] OR cardiac arrest[tiab] OR cardiopulmonary resuscit*[tiab]) AND (Surgery[MeSH Terms] OR Surgical Procedures, Operative[MeSH Terms] OR surgery[tiab] OR surg*[tiab] OR anesthesia[tiab] OR anaesthesia[tiab] OR anesth*[tiab] OR Intensive Care[tiab] OR ICU[tiab] OR critical*[tiab]) AND (Analgesics, Opioid[MeSH Terms] OR morphine[tiab] OR fentanyl[tiab] OR remifentan*[tiab] OR sufentanyl[tiab] OR alfentanyl[tiab] OR codeine[tiab] OR opioid[tiab] OR opiate[tiab] OR naloxone[tiab])

**Search equation EMBASE**

('cardiac arrest'/exp OR 'cardiac arrest' OR 'respiratory arrest'/exp OR 'respiratory arrest') AND ('surgery'/exp OR 'diagnosis, surgical' OR 'diagnostic techniques, surgical' OR 'operation' OR 'operation care' OR 'operative intervention' OR 'operative repair' OR 'operative restoration' OR 'operative surgical procedure' OR 'operative treatment' OR 'research surgery' OR 'resection' OR 'specialties, surgical' OR 'surgery' OR 'surgery, operative' OR 'surgical care' OR 'surgical correction' OR 'surgical exposure' OR 'surgical intervention' OR 'surgical management' OR 'surgical operation' OR 'surgical practice' OR 'surgical procedures, operative' OR 'surgical repair' OR 'surgical research' OR 'surgical restoration' OR 'surgical service' OR 'surgical speciality' OR 'surgical specialty' OR 'surgical therapy' OR 'surgical treatment' OR 'anesthesia'/exp OR 'anaesthesia' OR 'anaesthesia, auto' OR 'anaesthetic action' OR 'anesthesia' OR 'anesthesia, auto' OR 'anesthetic action' OR 'anesthetization' OR 'animal anaesthesia' OR 'animal anesthesia' OR 'autoanaesthesia' OR 'autoanesthesia' OR 'drop mask anaesthesia' OR 'drop mask anesthesia' OR 'narcosis' OR 'neuroanaesthesia' OR 'neuroanesthesia' OR 'short anaesthesia' OR 'short anesthesia' OR 'short duration anaesthesia' OR 'short duration anesthesia' OR 'intensive care'/exp OR 'care, intensive' OR 'critical care' OR 'intensive care' OR 'intensive care, paediatric' OR 'intensive care, pediatric' OR 'intensive therapy' OR 'paediatric intensive care' OR 'pediatric intensive care' OR 'therapy, intensive') AND ('opioid'/exp OR 'opioid')

**Search equation COCHRANE LIBRARY**

"Heart Arrest" in Title Abstract Keyword AND "Surgery" in Title Abstract Keyword AND "naloxone" in Title Abstract Keyword - (Word variations have been searched)

**Characteristics of included studies**

| Study | Design | Patients | Interventions | Outcomes |
| --- | --- | --- | --- | --- |
| - | - | - | - | Primary: - |
|  |  |  |  | Secondary: - |

**Grade evidence table**

| **Outcomes** | **Quality assessment** | | | | | | | **Summary of findings** | | | | | | |
| --- | --- | --- | --- | --- | --- | --- | --- | --- | --- | --- | --- | --- | --- | --- |
|  |  |  |  |  |  |  |  | **No of patients** | | **Effect** | | | **Quality of evidence**  **(GRADE)** | |
|  | **No of participants**  **(studies)** | **Design** | **Risk of bias** | **Inconsistency** | **Indirectness** | **Imprecision** | **Other**  **considerations** | **Any specific alteration in treatment algorithm** | **Standard care** | **Relative**  **(95% CI)** | **Risk**  **difference with any specific alteration in treatment algorithm** | **Absolute** |  |  |
| Survival with favorable neurologic/functional outcome at discharge, 30 days, 60 days, 180 days, and/or 1 year; survival only at discharge, 30 days, 60 days, 180 days, and/or 1 year; ROSC | – | - | - | - | - | - | - | - | - | - | - | - | - |  |

**Consensus of science statement**

In patients with PERIOPCA due to opioid toxicity, it might be reasonable to administer specific agents in addition to advanced life support (COR/LOE: IIb/C-EO).

**References**

None.

**PICO 13**

Among adults who are in cardiac arrest in the perioperative setting (P), does corticosteroid or mineralocorticoid administration during and/or after CPR or the combined use of vasopressin, epinephrine, and steroids during and/or after CPR (I), compared with not using steroids or epinephrine alone during CPR and no steroids after CPR (C), change survival with favorable neurologic/functional outcome at discharge, 30 days, 60 days, 180 days, and/or 1 year; survival only at discharge, 30 days, 60 days, 180 days, and/or 1 year; ROSC (O)?

**Search equation PUBMED**

(((((((("cardiac arrest"[Title/Abstract]) OR "cardiopulmonary arrest"[Title/Abstract]) OR "circulatory arrest"[Title/Abstract]) OR "heart arrest"[Title/Abstract]))) AND ((corticosteroid[Title/Abstract]) OR mineralocorticoid[Title/Abstract])) AND ((((vasopressin[Title/Abstract]) OR epinephrine[Title/Abstract]) OR adrenaline[Title/Abstract]) OR steroids[Title/Abstract])) AND ((((((((survival[Title/Abstract]) OR "neurological outcome"[Title/Abstract]) OR "functional outcome"[Title/Abstract]) OR "Hospital discharge"[Title/Abstract]) OR discharge[Title/Abstract])))

**Search equation EMBASE**

"('cardiac arrest':ti,ab,kw OR 'cardiopulmonary arrest':ti,ab,kw OR 'circulatory arrest':ti,ab,kw OR 'heart arrest':ti,ab,kw) AND (corticosteroid:ti,ab,kw OR mineralocorticoid:ti,ab,kw) AND (vasopressin:ti,ab,kw OR epinephrine:ti,ab,kw OR adrenaline:ti,ab,kw OR steroids:ti,ab,kw) AND (survival:ti,ab,kw OR 'neurological outcome':ti,ab,kw OR 'functional outcome':ti,ab,kw OR 'hospital discharge':ti,ab,kw OR discharge:ti,ab,kw)"

**Search equation Cochrane Library**

“cardiac arrest” OR “heart arrest” in Title Abstract Keyword AND “perioperative” OR “operating room” OR “intraoperative” in Title Abstract Keyword AND “corticosteroid” OR “mineralocorticoid” OR “vasopressin and epinephrine and steroids” - (Word variations have been searched)

**Study selection**

24 records screened

22 articles discarded (duplicates or excluded after title and abstract evaluation)

0 full-text article excluded due to absence of comparator

2 study included in qualitative synthesis

**Characteristics of included studies**

| Study | Design | Patients | Interventions | Outcomes |
| --- | --- | --- | --- | --- |
| Mentzelopoulos et al, 2009 | Randomized controlled trial | 100 | combined use of vasopressin, and epinephrine after 5^th^ cycle of CPR | Primary: Return of spontaneous circulation |
|  |  |  |  | Secondary: Survival to discharge |
| Mentzelopoulos et al, 2013 | Randomized controlled trial | 268 | combined use of vasopressin, and epinephrine after 1^st^ cycle of CPR. After that Methylprednisolone | Primary: Return of spontaneous circulation |
|  |  |  |  | Secondary:  Survival to discharge with good functional outcomes |

**Grade evidence table**

| **Outcomes** | **Quality assessment** | | | | | | | **Summary of findings** | | | | | |
| --- | --- | --- | --- | --- | --- | --- | --- | --- | --- | --- | --- | --- | --- |
|  |  |  |  |  |  |  |  | **No of patients** | | **Effect** | | | **Quality of evidence**  **(GRADE)** |
|  | **No of participants**  **(studies)** | **Design** | **Risk of bias** | **Inconsistency** | **Indirectness** | **Imprecision** | **Other**  **considerations** | **Corticosteroid or mineralocorticoid administration or the combined use of vasopressin, epinephrine, and steroids during/after CPR** | **Not using steroids or epinephrine alone during CPR and no steroids after CPR** | **Relative**  **(95% CI)** | **Absolute** | **Risk**  **difference with corticosteroid or mineralocorticoid administration or the combined use of vasopressin, epinephrine, and steroids during/after CPR** |  |
| Return of spontaneous circulation | 368 (2) | Randomized controlled trial | Not serious | Not serious | Not serious | Not serious | None | 148/178 | 118/190 | RR 1.34  (1.18 - 1.52) | 149 per 1.000 (59 to 330) | 98 more per 1.000 (from 8 more to 279 more) | ⊕⊕⊕⊕  High |
| Survival to discharge | 100 (1) | Randomized controlled trial | Not serious | Not serious | Not serious | Serious^a^ | None | 9/48 | 2/52 | RR 4.88  (1.11 - 21.43) | 188 per 1.000 (43 to 824) | 149 more per 1.000 (from 4 more to 786 more) | ⊕⊕⊕⊕  Moderate |
| Survival to discharge with good functional outcomes | 268 (1) | Randomized controlled trial | Not serious | Not serious | Not serious | Serious^b^ | None | 18/130 | 7/138 | RR 2.94  (1.16 - 6.50) | 832 per 1.000 (733 to 944) | 211 more per 1.000 (from 112 more to 323 more) | ⊕⊕⊕⊕  Moderate |

a Very wide CIs due to small number of events and participants. A case can be made for downgrading by two levels. Because it is consistent with the other study, it was reduced by only one level.

b Wide CIs due to small number of events.

**Consensus of science statement**

In adult patients with PERIOPCA, it is reasonable to administer corticosteroid or mineralocorticoid or the combination of vasopressin, epinephrine, and steroids during/after CPR to increase ROSC (COR/LOE: IIa/B-R). In these patients, these drugs can be useful for improving survival to discharge with good functional outcome (COR/LOE: IIa/B-R).


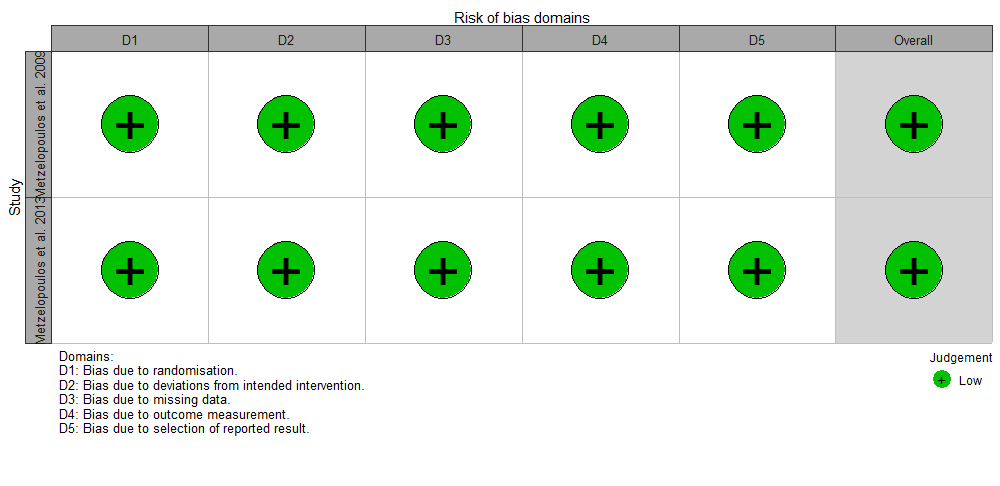


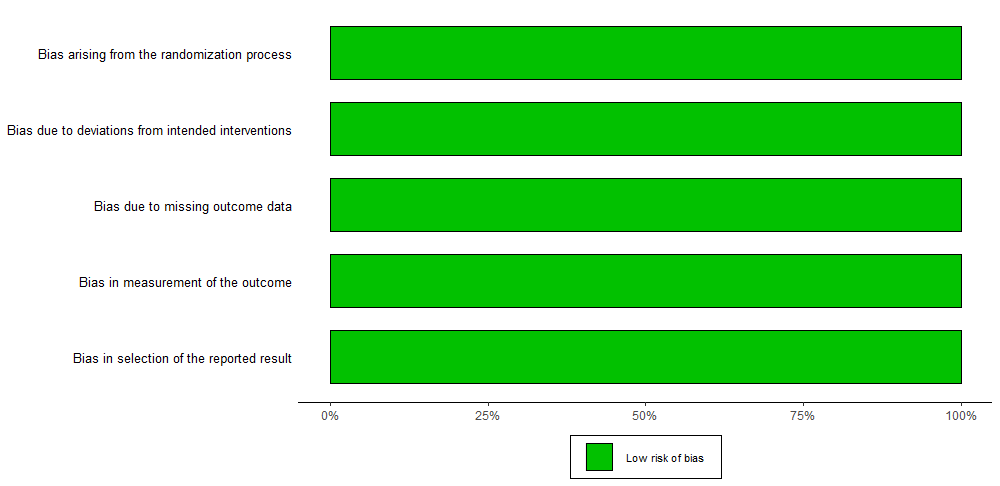


**References**

1. Mentzelopoulos SD, Malachias S, Chamos C, Konstantopoulos D, Ntaidou T, Papastylianou A, Kolliantzaki I, Theodoridi M, Ischaki H, Makris D, Zakynthinos E, Zintzaras E, Sourlas S, Aloizos S, Zakynthinos SG. Vasopressin, steroids, and epinephrine and neurologically favorable survival after in-hospital cardiac arrest: a randomized clinical trial. JAMA.2013;310:270-279.
2. Mentzelopoulos SD, Zakynthinos SG, Tzoufi M, Katsios N, Papastylianou A, Gkisioti S, Stathopoulos A, Kollintza A, Stamataki E, Roussos C. Vasopressin, epinephrine, and corticosteroids for in-hospital cardiac arrest. Arch Intern Med 2009;169:15-24.

**PICO 14**

In adult patients with cardiac arrest due to suspected drug toxicity (e.g., local anesthetics, tricyclic antidepressants, others) in the perioperative setting (P), does administration of IV lipid (I), compared with no IV lipid (C), change survival with favorable neurologic/functional outcome at discharge, 30 days, 60 days, 180 days, and/or 1 year; survival only at discharge, 30 days, 60 days, 180 days, and/or 1 year; ROSC (O)?

**Search equation PUBMED**

(Heart Arrest[MeSH Terms] OR Cardiopulmonary Arrest[MeSH Terms] OR cardiac arrest[tiab] OR cardiopulmonary resuscit*[tiab]) AND (Surgery[MeSH Terms] OR Surgical Procedures, Operative[MeSH Terms] OR surgery[tiab] OR surg*[tiab] OR anesthesia[tiab] OR anaesthesia[tiab] OR anesth*[tiab] OR ICU[tiab] OR intensive care[tiab] OR critical*[tiab]) AND (Fat Emulsions, Intravenous/administration & dosage*)

**Search equation EMBASE**

('heart arrest'/exp OR 'arrest, heart' OR 'asystole' OR 'asystolia' OR 'asystoly' OR 'cardiac arrest' OR 'circulation arrest' OR 'circulatory arrest' OR 'heart arrest' OR 'heart arrest, induced' OR 'heart asystole' OR 'heart standstill') AND ('drug toxicity and intoxication'/exp OR 'drug toxicity and intoxication') AND ('intralipid'/exp OR 'intralipid' OR 'intralipid 10' OR 'intralipid 10%' OR 'intralipid 20%' OR 'intralipid 30%' OR 'intralipide' OR 'oil emulsion infusion' OR 'soybean oil emulsion infusion' OR 'lipid emulsion'/exp OR 'emulsion, fat' OR 'emulsion, lipid' OR 'fat emulsion' OR 'fat emulsions, intravenous' OR 'lipid emulsion') AND ('surgery'/exp OR 'diagnosis, surgical' OR 'diagnostic techniques, surgical' OR 'operation' OR 'operation care' OR 'operative intervention' OR 'operative repair' OR 'operative restoration' OR 'operative surgical procedure' OR 'operative treatment' OR 'research surgery' OR 'resection' OR 'specialties, surgical' OR 'surgery' OR 'surgery, operative' OR 'surgical care' OR 'surgical correction' OR 'surgical exposure' OR 'surgical intervention' OR 'surgical management' OR 'surgical operation' OR 'surgical practice' OR 'surgical procedures, operative' OR 'surgical repair' OR 'surgical research' OR 'surgical restoration' OR 'surgical service' OR 'surgical speciality' OR 'surgical specialty' OR 'surgical therapy' OR 'surgical treatment' OR 'anesthesia'/exp OR 'anaesthesia' OR 'anaesthesia, auto' OR 'anaesthetic action' OR 'anesthesia' OR 'anesthesia, auto' OR 'anesthetic action' OR 'anesthetization' OR 'animal anaesthesia' OR 'animal anesthesia' OR 'autoanaesthesia' OR 'autoanesthesia' OR 'drop mask anaesthesia' OR 'drop mask anesthesia' OR 'narcosis' OR 'neuroanaesthesia' OR 'neuroanesthesia' OR 'short anaesthesia' OR 'short anesthesia' OR 'short duration anaesthesia' OR 'short duration anesthesia' OR 'intensive care'/exp OR 'care, intensive' OR 'critical care' OR 'intensive care' OR 'intensive care, paediatric' OR 'intensive care, pediatric' OR 'intensive therapy' OR 'paediatric intensive care' OR 'pediatric intensive care' OR 'therapy, intensive')

**Search equation COCHRANE LIBRARY**

"Heart Arrest" in Title Abstract Keyword AND "Surgery" in Title Abstract Keyword AND "lipid" OR "intralipid" in Title Abstract Keyword - (Word variations have been searched)

**Characteristics of included studies**

| Study | Design | Patients | Interventions | Outcomes |
| --- | --- | --- | --- | --- |
| Rosenblatt et al. | Case report | 1 | 20% lipid emulsion (Intralipid) | Primary: Return of spontaneous circulation, Survival with favorable neurologic/functional at discharge, Survival at discharge |
|  |  |  |  | Secondary: |
| Smith et al. | Case report | 1 | 20% lipid emulsion (Intralipid) | Primary: Return of spontaneous circulation, Survival with favorable neurologic/functional at discharge, Survival at discharge |
|  |  |  |  | Secondary: |
| Marwick et al. | Case report | 1 | 20% lipid emulsion (Intralipid) | Primary: Return of spontaneous circulation, Survival with favorable neurologic/functional at discharge, Survival at discharge |
|  |  |  |  | Secondary: |
| Whiteman et al. | Case report | 1 | 20% lipid emulsion (Intralipid) | Primary: Return of spontaneous circulation, Survival with favorable neurologic/functional at discharge, Survival at discharge |
|  |  |  |  | Secondary: |
| Weber et al. | Case report | 1 | 20% lipid emulsion (Intralipid) | Primary: Return of spontaneous circulation, Survival with favorable neurologic/functional at discharge, Survival at discharge |
|  |  |  |  | Secondary: |
| Hasan et al. | Case report | 1 | 20% lipid emulsion (Intralipid) | Primary: Return of spontaneous circulation, Survival with favorable neurologic/functional at discharge, Survival at discharge |
|  |  |  |  | Secondary: |
| Gnaho et al. | Case report | 1 | 20% lipid emulsion (Intralipid) | Primary: Return of spontaneous circulation, Survival with favorable neurologic/functional at discharge, Survival at discharge |
|  |  |  |  | Secondary: |
| Warren et al. | Case report | 1 | 20% lipid emulsion (Liposyn III) | Primary: Return of spontaneous circulation, Survival with favorable neurologic/functional at discharge, Survival at discharge |
|  |  |  |  | Secondary: |
| Sonsino et al. | Case report | 1 | lipid emulsion (Kabiven) | Primary: Return of spontaneous circulation* (patient died of bronchopneumonia during hospital admission) |
|  |  |  |  | Secondary: |
| Scherrer et al. | Case report | 1 | 20% lipid emulsion | Primary: Return of spontaneous circulation, Survival with favorable neurologic/functional at discharge, Survival at discharge |
|  |  |  |  | Secondary: |
| Mazoit et al. | Case report | 1 | 20% lipid emulsion (Intralipid) | Primary: Return of spontaneous circulation, Survival with favorable neurologic/functional at discharge, Survival at discharge |
|  |  |  |  | Secondary: |
| Litz et al. | Case report | 1 | 20% lipid emulsion (Intralipid) | Primary: Return of spontaneous circulation, Survival with favorable neurologic/functional at discharge, Survival at discharge |
|  |  |  |  | Secondary: |
| Markowitz et al. | Case report | 1 | 20% lipid emulsion (Intralipid) | Primary: Return of spontaneous circulation, Survival with favorable neurologic/functional at discharge, Survival at discharge |
|  |  |  |  | Secondary: |

**Grade evidence table**

| **Outcomes** | **Quality assessment** | | | | | | | **Summary of findings** | | | | | | |
| --- | --- | --- | --- | --- | --- | --- | --- | --- | --- | --- | --- | --- | --- | --- |
|  |  |  |  |  |  |  |  | **No of patients** | | **Effect** | | | **Quality of evidence**  **(GRADE)** | |
|  | **No of participants**  **(studies)** | **Design** | **Risk of bias** | **Inconsistency** | **Indirectness** | **Imprecision** | **Other**  **considerations** | **Administration of IV lipid** | **No IV lipid** | **Relative**  **(95% CI)** | **Risk**  **difference with IV lipid** | **Absolute** |  |  |
| Return of spontaneous circulation | 13 (13) | Observational studies | Serious^a^ | Not serious | Not serious | Very serious^a^ | - | 13 | 0 | - | - | - | ⊕🌕🌕🌕  Very low |  |
| Survival with favorable neurologic/functional at discharge | 12 (13) | Observational studies | Serious^a,b^ | Not serious | Not serious | Very serious^a^ | - | 13 | 0 | - | - | - | ⊕🌕🌕🌕  Very low |  |
| Survival at discharge | 12 (13) | Observational studies | Serious^a,b^ | Not serious | Not serious | Very serious^a^ | - | 13 | 0 | - | - | - | ⊕🌕🌕🌕  Very low |  |

^a^ All the collected evidence for the specified outcomes derives from case reports describing perioperative cardiac arrest cases due to local anesthetic systemic toxicity

^b^ In one of the sited studies (Sonsino et al.), the patient’s death was not directly related to cardiac arrest due to drug toxicity (bronchopneumonia)

**Consensus of science statement**

In adult patients with PERIOPCA due to confirmed or suspected LAST, it may be reasonable to use lipid therapy (COR/LOE: IIb/C-LD).

**References**

1. Rosenblatt MA, Abel M, Fischer GW, Itzkovich CJ, Eisenkraft JB. Successful use of a 20% lipid emulsion to resuscitate a patient after a presumed bupivacaine-related cardiac arrest. Anesthesiology 2006;105:217-218.
2. Smith HM, Jacob AK, Segura LG, Dilger JA, Torsher LC. Simulation education in anesthesia training: a case report of successful resuscitation of bupivacaine-induced cardiac arrest linked to recent simulation training. Anesth Analg 2008;106:1581-1584, table of contents.
3. Marwick PC, Levin AI, Coetzee AR. Recurrence of cardiotoxicity after lipid rescue from bupivacaine-induced cardiac arrest. Anesth Analg 2009;108:1344-1346.
4. Whiteman DM, Kushins SI. Successful Resuscitation With Intralipid After Marcaine Overdose. Aesthet Surg J 2014;34:738-740.
5. Weber F, Guha R, Weinberg G, Steinbach F, Gitman M. Prolonged Pulseless Electrical Activity Cardiac Arrest After Intranasal Injection of Lidocaine With Epinephrine: A Case Report A A Pract 2019;12:438-440.
6. Hasan B, Asif T, Hasan M. Lidocaine-Induced Systemic Toxicity: A Case Report and Review of Literature. Cureus 2017;9:e1275.
7. Gnaho A, Eyrieux S, Gentili M. Cardiac arrest during an ultrasound-guided sciatic nerve block combined with nerve stimulation. Reg Anesth Pain Med 2009;34:278.
8. Warren JA, Thoma RB, Georgescu A, Shah SJ. Intravenous lipid infusion in the successful resuscitation of local anesthetic-induced cardiovascular collapse after supraclavicular brachial plexus block. Anesth Analg 2008;106:1578-1580, table of contents.
9. Sonsino DH, Fischler M. Immediate intravenous lipid infusion in the successful resuscitation of ropivacaine-induced cardiac arrest after infraclavicular brachial plexus block. Reg Anesth Pain Med 2009;34:276-277.
10. Scherrer V, Compere V, Loisel C, Dureuil B. Cardiac arrest from local anesthetic toxicity after a field block and transversus abdominis plane block: a consequence of miscommunication between the anesthesiologist and surgeon. A A Case Rep 2013;1:75-76.
11. Mazoit JX. Arrêt cardiaque et anesthésiques locaux [Cardiac arrest and local anaesthetics]. Presse Med 2013;42:280-286.
12. Litz RJ, Popp M, Stehr SN, Koch T. Successful resuscitation of a patient with ropivacaine-induced asystole after axillary plexus block using lipid infusion. Anaesthesia 2006;61:800-801.
13. Markowitz S, Neal JM. Immediate lipid emulsion therapy in the successful treatment of bupivacaine systemic toxicity. Reg Anesth Pain Med 2009;34:276.

**PICO 15**

Among adults who are in cardiac arrest in the perioperative setting (P), does use of ultrasound (including echocardiography or other organ assessments) during CPR (I), compared with conventional CPR and resuscitation without use of ultrasound (C), change survival with favorable neurologic/functional outcome at discharge, 30 days, 60 days, 180 days, and/or 1 year; survival only at discharge, 30 days, 60 days, 180 days, and/or 1 year; ROSC (O)?

**Search equation PUBMED**

"Heart Arrest"[Mesh:NoExp] OR "Cardiopulmonary Resuscitation"[Mesh:NoExp] OR “Cardiac arrest” [Title/Abstract] OR “Cardiac arrests” [Title/Abstract] OR “Heart Arrest” [Title/Abstract] OR “Heart Arrests” [Title/Abstract] OR “cardiopulmonary arrest"[Title/Abstract] OR “cardiopulmonary arrests”[Title/Abstract] OR "Cardiopulmonary resuscitation"[Title/Abstract] OR “Cardio Pulmonary resuscitation” [Title/Abstract] AND (((“echocardiography”[Title/Abstract] OR “ultrasound”[Title/Abstract] OR “organ assessment”[Title/Abstract] OR "echo"[Mesh] AND ("mortality"[Subheading] OR "mortality” [Title/Abstract] OR survival[Title/Abstract] OR "survival"[MeSH Terms] OR “return spontaneous circulation” [Title/Abstract] OR resuscitation[Title/Abstract] OR resuscitated[Title/Abstract] OR "Resuscitation"[Mesh] OR “Cerebral performance category” [Title/Abstract] OR "cerebral performance categories"[Title/Abstract] NOT "Out-of-Hospital Cardiac Arrest"[Mesh] OR “out of hospital”.

**Search equation EMBASE**

'heart arrest'/de OR 'cardiopulmonary arrest'/de OR 'cardiac arrest*':ti,ab OR 'heart arrest*':ti,ab OR 'cardiopulmonary arrest*':ti,ab OR 'cardiopulmonary resuscitation':ti,ab OR 'cardio pulmonary resuscitation':ti,ab AND 'mortality'/exp OR mortality:ti,ab OR survival:ti,ab OR 'survival'/exp OR 'return spontaneous circulation':ti,ab OR resuscitation:ti,ab OR resuscitated:ti,ab OR 'resuscitation'/exp AND 'echocardiography ':ti,ab OR 'ultrasound':ti,ab OR 'organ assessment:ti,ab OR 'echo'/exp NOT 'out of hospital cardiac arrest'/exp OR 'out of hospital' OR 'out of hospitals'

**Search equation Cochrane Library**

“Cardiac arrest” OR “Cardiac arrests” OR “Heart Arrest” OR “Heart Arrests” OR “cardiopulmonary arrest" OR “cardiopulmonary arrests” OR "Cardiopulmonary resuscitation" OR “Cardio Pulmonary resuscitation” in Title Abstract Keyword AND “ultrasound” OR “echocardiography” OR “echo” OR “organ assessment” in Title Abstract Keyword - (Word variations have been searched)

**Study selection**

160 records screened

160 articles discarded (duplicates or excluded after title and abstract evaluation)

0 full-text article excluded due to absence of comparator

0 study included in qualitative synthesis

**Characteristics of included studies**

| Study | Design | Patients | Interventions | Outcomes |
| --- | --- | --- | --- | --- |
| - | - | - | - | Primary: - |
|  |  |  |  | Secondary: - |

**Grade evidence table**

| **Outcomes** | **Quality assessment** | | | | | | | **Summary of findings** | | | | | | |
| --- | --- | --- | --- | --- | --- | --- | --- | --- | --- | --- | --- | --- | --- | --- |
|  |  |  |  |  |  |  |  | **No of patients** | | **Effect** | | | **Quality of evidence**  **(GRADE)** | |
|  | **No of participants**  **(studies)** | **Design** | **Risk of bias** | **Inconsistency** | **Indirectness** | **Imprecision** | **Other**  **considerations** | **Use of ultrasound during CPR** | **Conventional CPR and resuscitation without use of ultrasound** | **Relative**  **(95% CI)** | **Risk**  **difference with use of ultrasound during CPR** | **Absolute** |  |  |
| Survival with favorable neurologic/functional outcome at discharge, 30 days, 60 days, 180 days, and/or 1 year; survival only at discharge, 30 days, 60 days, 180 days, and/or 1 year; ROSC | – | - | - | - | - | - | - | - | - | - | - | - | - |  |

**Consensus of science statement**

In patients with PERIOPCA, it may be reasonable to use point-of-care ultrasound to improve CPR and increase survival rates (COR/LOE: IIb/C-EO).

**References**

None.

**PICO 16**

Among adults who are in cardiac arrest in the perioperative setting (P), does the use of ECPR techniques (including extracorporeal membrane oxygenation or cardiopulmonary bypass) (I), compared with manual CPR or mechanical CPR (C), change survival with favorable neurologic/functional outcome at discharge, 30 days, 60 days, 180 days, and/or 1 year; survival only at discharge, 30 days, 60 days, 180 days, and/or 1 year; ROSC (O)?

**Search equation PUBMED**

(((((((("cardiac arrest"[Title/Abstract]) OR "cardiopulmonary arrest"[Title/Abstract]) OR "circulatory arrest"[Title/Abstract]) OR "heart arrest"[Title/Abstract]))) AND ((((CPR[Title/Abstract]) OR "conventional-CPR"[Title/Abstract]) OR "conventional CPR"[Title/Abstract]) OR "mechanical CPR"[Title/Abstract])) AND (((("extracorporeal cardiopulmonary resuscitation"[Title/Abstract]) OR ECPR[Title/Abstract]) OR "extracorporeal membrane oxygenation"[Title/Abstract]) OR "cardiopulmonary bypass"[Title/Abstract])) AND (((((((((survival[Title/Abstract]) OR "neurological outcome"[Title/Abstract]) OR "functional outcome"[Title/Abstract]) OR "Hospital discharge"[Title/Abstract]) OR discharge[Title/Abstract]))))

**Search equation EMBASE**

"('cardiac arrest':ti,ab,kw OR 'cardiopulmonary arrest':ti,ab,kw OR 'circulatory arrest':ti,ab,kw OR 'heart arrest':ti,ab,kw) AND (cpr:ti,ab,kw OR 'conventional-cpr':ti,ab,kw OR 'conventional cpr':ti,ab,kw OR 'mechanical cpr':ti,ab,kw) AND ('extracorporeal cardiopulmonary resuscitation':ti,ab,kw OR ecpr:ti,ab,kw OR 'extracorporeal membrane oxygenation':ti,ab,kw OR 'cardiopulmonary bypass':ti,ab,kw) AND (survival:ti,ab,kw OR 'neurological outcome':ti,ab,kw OR 'functional outcome':ti,ab,kw OR 'hospital discharge':ti,ab,kw OR discharge:ti,ab,kw)"

**Search equation Cochrane Library**

“Cardiac arrest” OR “Cardiac arrests” OR “Heart Arrest” OR “Heart Arrests” OR “cardiopulmonary arrest" OR “cardiopulmonary arrests" OR "Cardiopulmonary resuscitation" OR “Cardio Pulmonary resuscitation” in Title Abstract Keyword AND “extracorporeal membrane oxygenation” OR “cardiopulmonary bypass” OR “extracorporeal cardiopulmonary resuscitation” OR “extracorporeal CPR” OR “ECPR” in Title Abstract Keyword - (Word variations have been searched)

**Study selection**

585 records screened

583 articles discarded (duplicates or excluded after title and abstract evaluation)

0 full-text article excluded due to absence of comparator

2 studies included in qualitative synthesis

**Characteristics of included studies**

| Study | Design | Patients | Interventions | Outcomes |
| --- | --- | --- | --- | --- |
| Lin et al. | Observational study | 118 | Extracorporeal rescue and conventional  resuscitation | Primary: ROSB - ROSC |
|  |  |  |  | Secondary: Survival at 30 days, one, two, six months and one year |
| Avalli et al. | Observational study | 42 | Extracorporeal rescue and conventional  resuscitation | Primary: Survival - one year with good neurological outcomes |
|  |  |  |  | Secondary: |

ROSB, return of spontaneous beating

**Grade evidence table**

| **Outcomes** | **Quality assessment** | | | | | | | **Summary of findings** | | | | | |
| --- | --- | --- | --- | --- | --- | --- | --- | --- | --- | --- | --- | --- | --- |
|  |  |  |  |  |  |  |  | **No of patients** | | **Effect** | | | **Quality of evidence**  **(GRADE)** |
|  | **No of participants**  **(studies)** | **Design** | **Risk of bias** | **Inconsistency** | **Indirectness** | **Imprecision** | **Other**  **considerations** | **ECPR techniques** | **Manual or mechanical CPR** | **Relative**  **(95% CI)** | **Absolute** | **Risk**  **difference with ECPR techniques** |  |
| Return of spontaneous circulation | 118 (1) | Observational study | Very serious^a^ | Not serious | Not serious | Serious^b^ | None | 63/63 | 55/55 | RR 1.00  (0.97 - 1.03) | 1000 per 1.000  (970 to 1.000) | 0 fewer per 1.000 (from 30 fewer to 30 more) | ⊕🌕🌕🌕  Very low |
| Survival to discharge | 118 (1) | Observational study | Serious^c^ | Not serious | Not serious | Serious^b^ | None | 16/63 | 14/55 | RR 1.00  (0.54 - 1.85) | 255 per 1.000  (137 to 471) | 0 fewer per 1.000 (from 117 fewer to 216 more) | ⊕🌕🌕🌕  Very low |
| Survival to discharge with good neurologic outcome | 118 (1) | Observational study | Serious^c^ | Not serious | Not serious | Serious^b^ | None | 13/63 | 12/55 | RR 0.95  (0.47 - 1.90) | 207 per 1.000  (103 to 415) | 11 fewer per 1.000 (from 116 fewer to 196 more) | ⊕🌕🌕🌕  Very low |
| Survival at 30 days | 118 (1) | Observational study | Serious^c^ | Not serious | Not serious | Serious^b^ | None | 19/63 | 17/55 | RR 0.98  (0.57 - 1.68) | 303 per 1.000  (176 to 519) | 6 more per 1.000 (from 113 fewer to 210 more) | ⊕🌕🌕🌕  Very low |
| Survival at 6 months | 118 (1) | Observational study | Serious^c^ | Not serious | Not serious | Serious^b^ | None | 16/63 | 13/55 | RR 1.07  (0.57 - 2.03) | 253 per 1.000  (135 to 480) | 17 more per 1.000 (from 102 fewer to 243 more) | ⊕🌕🌕🌕  Very low |
| Survival at one year | 118 (1) | Observational study | Serious^c^ | Not serious | Not serious | Serious^b^ | None | 11/63 | 11/55 | RR 0.87  (0.41 - 1.85) | 174 per 1.000  (82 to 370) | 26 fewer per 1.000 (from 118 fewer to 170 more) | ⊕🌕🌕🌕  Very low |
| Survival at one year with good neurological outcome | 126 (1) | Observational study | Serious^c^ | Not serious | Not serious | Serious^b^ | None | 8/63 | 10/63 | RR 0.70  (0.30 - 1.64) | 111 per 1.000  (48 to 260) | 48 fewer per 1.000 (from 111 fewer to 102 more) | ⊕🌕🌕🌕  Very low |

^a^ It was a prerequisite to have ROSC, in order to be included in the study

^b^ CIs cross the 1

^c^ It is measured only in the subpopulation of those who had ROSC

**Consensus of science statement**

In adult patients with PERIOPCA, it may be reasonable to use ECPR as a rescue therapy when CPR has failed to provide ROSC or non-sustained ROSC (COR/LOE: IIb/C-LD).


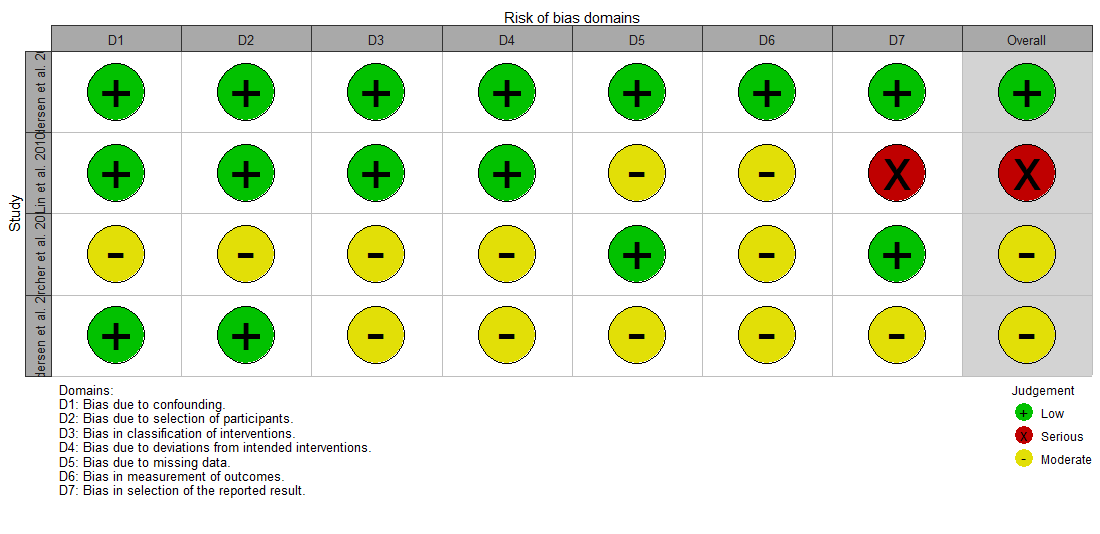


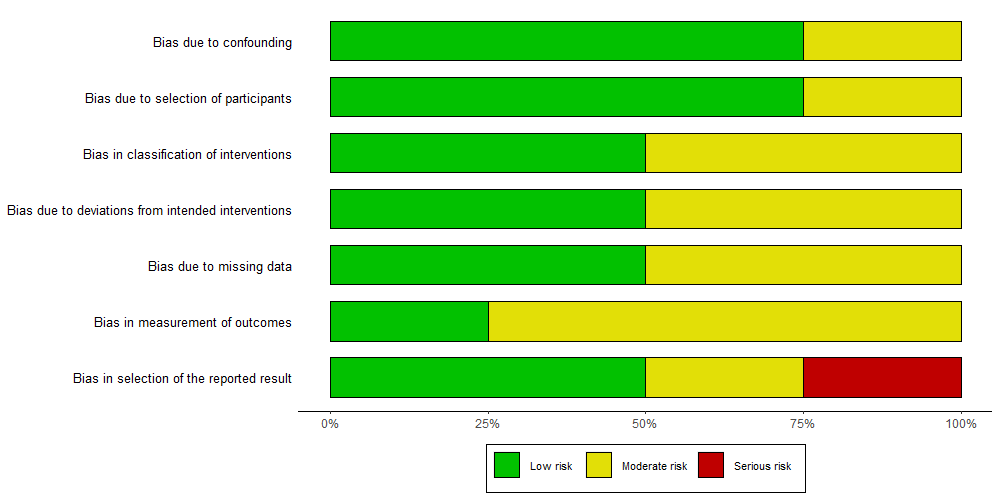


**References**

1. Lin JW, Wang MJ, Yu HY, Wang CH, Chang WT, Jerng JS, Huang SC, Chou NK, Chi NH, Ko WJ, Wang YC, Wang SS, Hwang JJ, Lin FY, Chen YS. Comparing the survival between extracorporeal rescue and conventional resuscitation in adult in-hospital cardiac arrests: propensity analysis of three-year data. Resuscitation 2010;81:796-803.
2. Avalli L, Maggioni E, Formica F, Redaelli G, Migliari M, Scanziani M, Celotti S, Coppo A, Caruso R, Ristagno G, Fumagalli R. Favourable survival of in-hospital compared to out-of-hospital refractory cardiac arrest patients treated with extracorporeal membrane oxygenation: an Italian tertiary care centre experience. Resuscitation 2012;83:579-583.

**PICO 17**

Among adults with ROSC after cardiac arrest in the perioperative setting (P), does titration of therapy to achieve a specific hemodynamic goal (e.g., MAP greater than 65 mm Hg) (I), compared with no hemodynamic goal (C), change survival with favorable neurologic/functional outcome at discharge, 30 days, 60 days, 180 days, and/or 1 year; survival at discharge, 30 days, 60 days, 180 days, and/or 1 year (O)?

**Search equation PUBMED**

(((((((("cardiac arrest"[Title/Abstract]) OR "cardiopulmonary arrest"[Title/Abstract]) OR "circulatory arrest"[Title/Abstract]) OR "heart arrest"[Title/Abstract]))) AND (("Return of spontaneous circulation"[Title/Abstract]) OR ROSC[Title/Abstract])) AND ((((((("hemodynamic goals"[Title/Abstract]) OR "mean arterial pressure"[Title/Abstract]) OR "systolic blood pressure"[Title/Abstract]) OR SBP[Title/Abstract]) OR MAP[Title/Abstract]) OR "goal-directed therapy"[Title/Abstract]) OR "goal directed therapy")) AND ((((((((survival[Title/Abstract]) OR "neurological outcome"[Title/Abstract]) OR "functional outcome"[Title/Abstract]) OR "Hospital discharge"[Title/Abstract]) OR discharge[Title/Abstract])))

**Search equation EMBASE**

"('cardiac arrest':ti,ab,kw OR 'cardiopulmonary arrest':ti,ab,kw OR 'circulatory arrest':ti,ab,kw OR 'heart arrest':ti,ab,kw) AND ('return of spontaneous circulation':ti,ab,kw OR rosc:ti,ab,kw) AND ('hemodynamic goals':ti,ab,kw OR 'mean arterial pressure':ti,ab,kw OR 'systolic blood pressure':ti,ab,kw OR sbp:ti,ab,kw OR map:ti,ab,kw OR 'goal-directed therapy':ti,ab,kw OR 'goal directed therapy':ti,ab,kw) AND (survival:ti,ab,kw OR 'neurological outcome':ti,ab,kw OR 'functional outcome':ti,ab,kw OR 'hospital discharge':ti,ab,kw OR discharge:ti,ab,kw)"

**Search equation Cochrane Library**

“Cardiac arrest” OR “Cardiac arrests” OR “Heart Arrest” OR “Heart Arrests” OR “cardiopulmonary arrest" OR “cardiopulmonary arrests" OR "Cardiopulmonary resuscitation" OR “Cardio Pulmonary resuscitation” in Title Abstract Keyword AND “hemodynamic goal” OR “mean arterial pressure” OR “systolic blood pressure” OR “goal-directed therapy” OR “goal directed therapy” in Title Abstract Keyword - (Word variations have been searched)

**Study selection**

302 records screened

302 articles discarded (duplicates or excluded after title and abstract evaluation)

0 full-text article excluded due to absence of comparator

0 studies included in qualitative synthesis

**Characteristics of included studies**

| Study | Design | Patients | Interventions | Outcomes |
| --- | --- | --- | --- | --- |
| - | - | - | - | Primary: - |
|  |  |  |  | Secondary: - |

**Grade evidence table**

| **Outcomes** | **Quality assessment** | | | | | | | **Summary of findings** | | | | | | |
| --- | --- | --- | --- | --- | --- | --- | --- | --- | --- | --- | --- | --- | --- | --- |
|  |  |  |  |  |  |  |  | **No of patients** | | **Effect** | | | **Quality of evidence**  **(GRADE)** | |
|  | **No of participants**  **(studies)** | **Design** | **Risk of bias** | **Inconsistency** | **Indirectness** | **Imprecision** | **Other**  **considerations** | **Ventilation to a PaO_2_ goal of <200 mmHg** | **PaO_2_ goal of >200 mmHg** | **Relative**  **(95% CI)** | **Risk**  **difference with ventilation to a PaO_2_ goal of <200 mmHg** | **Absolute** |  |  |
| Survival with favorable neurologic/functional outcome at discharge, 30 days, 60 days, 180 days, and/or 1 year; survival at discharge, 30 days, 60 days, 180 days, and/or 1 year | – | - | - | - | - | - | - | - | - | - | - | - | - |  |

**Consensus of science statement**

In patients with ROSC after PERIOPCA, it may be reasonable to target the hemodynamics goals to optimize tissue perfusion as indicated by an adequate urine output (1 ml kg^−1^ h^−1^) and normal or decreasing plasma lactate values, taking into consideration the patient’s normal blood pressure, the cause of the arrest and the severity of any myocardial dysfunction (COR/LOE: IIb/C-EO).

**References**

None.

**PICO 18**

Among adults with ROSC after cardiac arrest in the perioperative setting (P), do prophylactic antiarrhythmic drugs given immediately after ROSC (I), compared with not giving antiarrhythmic drugs (C), change survival with favorable neurologic/functional outcome at discharge, 30 days, 60days, 180 days, and/or 1 year; development of cardiac arrest; survival only at discharge, 30 days, 60 days, 180 days, and/or 1 year; recurrence of ventricular fibrillation/pulseless ventricular tachycardia; incidence of arrhythmias (O)?

**Search equation PUBMED**

(((((((("cardiac arrest"[Title/Abstract]) OR "cardiopulmonary arrest"[Title/Abstract]) OR "circulatory arrest"[Title/Abstract]) OR "heart arrest"[Title/Abstract]))) AND (("Return of spontaneous circulation"[Title/Abstract]) OR ROSC[Title/Abstract])) AND (((((amiodarone[Title/Abstract]) OR lidocaine[Title/Abstract]) OR "anti-arrhythmic drugs"[Title/Abstract]) OR "antiarrhythmic drugs"[Title/Abstract]) OR "anti arrhythmic drugs"[Title/Abstract])) AND ((((((((((survival[Title/Abstract]) OR "neurological outcome"[Title/Abstract]) OR "functional outcome"[Title/Abstract]) OR "Hospital discharge"[Title/Abstract]) OR discharge[Title/Abstract]) OR "recurrence of VF"[Title/Abstract]) OR "incidence of arrhythmias"[Title/Abstract]))))

**Search equation EMBASE**

('cardiac arrest':ti,ab,kw OR 'cardiopulmonary arrest':ti,ab,kw OR 'circulatory arrest':ti,ab,kw OR 'heart arrest':ti,ab,kw) AND ('return of spontaneous circulation':ti,ab,kw OR rosc:ti,ab,kw) AND (amiodarone:ti,ab,kw OR lidocaine:ti,ab,kw OR 'anti-arrhythmic drugs':ti,ab,kw OR 'antiarrhythmic drugs':ti,ab,kw OR 'anti arrhythmic drugs':ti,ab,kw) AND (survival:ti,ab,kw OR 'neurological outcome':ti,ab,kw OR 'functional outcome':ti,ab,kw OR 'hospital discharge':ti,ab,kw OR discharge:ti,ab,kw OR 'recurrence of vf':ti,ab,kw OR 'incidence of arrhythmias':ti,ab,kw)

**Search equation Cochrane Library**

"cardiac arrest" OR "cardiopulmonary arrest" OR "circulatory arrest" OR "heart arrest" AND "Return of spontaneous circulation" OR ROSC AND amiodarone OR lidocaine OR "anti-arrhythmic drugs" OR "antiarrhythmic drugs" OR "anti arrhythmic drugs" AND survival OR "neurological outcome" OR "functional outcome" OR "Hospital discharge" OR discharge OR "recurrence of VF" OR "incidence of arrhythmias"

**Study selection**

122 records screened

122 articles discarded (duplicates or excluded after title and abstract evaluation)

0 full-text article excluded due to absence of comparator

0 studies included in qualitative synthesis

**Characteristics of included studies**

| Study | Design | Patients | Interventions | Outcomes |
| --- | --- | --- | --- | --- |
| - | - | - | - | Primary: - |
|  |  |  |  | Secondary: - |

**Grade evidence table**

| **Outcomes** | **Quality assessment** | | | | | | | **Summary of findings** | | | | | | |
| --- | --- | --- | --- | --- | --- | --- | --- | --- | --- | --- | --- | --- | --- | --- |
|  |  |  |  |  |  |  |  | **No of patients** | | **Effect** | | | **Quality of evidence**  **(GRADE)** | |
|  | **No of participants**  **(studies)** | **Design** | **Risk of bias** | **Inconsistency** | **Indirectness** | **Imprecision** | **Other**  **considerations** | **Ventilation to a PaO_2_ goal of <200 mmHg** | **PaO_2_ goal of >200 mmHg** | **Relative**  **(95% CI)** | **Risk**  **difference with ventilation to a PaO_2_ goal of <200 mmHg** | **Absolute** |  |  |
| Survival with favorable neurologic/functional outcome at discharge, 30 days, 60days, 180 days, and/or 1 year; development of cardiac arrest; survival only at discharge, 30 days, 60 days, 180 days, and/or 1 year; recurrence of ventricular fibrillation/pulseless ventricular tachycardia; incidence of arrhythmias | – | - | - | - | - | - | - | - | - | - | - | - | - |  |

**Consensus of science statement**

In the perioperative setting, it may be reasonable to administer antiarrhythmics immediately after ROSC to treat postresuscitation arrhythmias, especially in refractory cases, and prevent recurrences (COR/LOE: IIb/C-EO).

**References**

None.

**PICO 19**

Among successfully resuscitated perioperative cardiac arrest patients who receive mechanical ventilation (P), does permissive hypercapnia in the context of a lung-protective ventilatory strategy with low tidal volumes (i.e., 6 mL/kg predicted body weight) and PEEP/FiO_2_ set according to the ARDSnet protocol (I), compared with normocapnia in the context of a ventilatory strategy employing moderate tidal volumes (i.e., 7.5-10 mL/kg predicted body weight and PEEP levels of 5-10 cmH_2_O (C), change survival with favorable neurologic/functional outcome at discharge, 30 days, 60 days, 180 days, and/or 1 year; survival only at discharge, 30 days, 60 days, 180 days, and/or 1 year (O)?

**Search equation PubMed**

(Heart Arrest[MeSH Terms] OR Cardiopulmonary Arrest[MeSH Terms] OR cardiac arrest[tiab] OR cardiopulmonary resuscit*[tiab]) AND ("Hypercapnia/therapy"[MeSH Terms] OR “protective ventilation”[tiab] OR “low volume”[tiab] OR ARDSnet[tiab])

**Search equation Embase**

('heart arrest'/exp OR 'arrest, heart' OR 'asystole' OR 'asystolia' OR 'asystoly' OR 'cardiac arrest' OR 'circulation arrest' OR 'circulatory arrest' OR 'heart arrest' OR 'heart arrest, induced' OR 'heart asystole' OR 'heart standstill') AND ('protective ventilation' OR 'permissive hypercapnia')

**Search equation Cochrane library**

"Heart Arrest" AND "Surgery" AND "normocapnia" OR “hypercapnia” OR “protective ventilation”

**Study selection**

122 records screened

122 articles discarded (duplicates or excluded after title and abstract evaluation)

0 full-text article excluded due to absence of comparator

0 studies included in qualitative synthesis

**Characteristics of included studies**

| Study | Design | Patients | Interventions | Outcomes |
| --- | --- | --- | --- | --- |
| NA | - | - | - | Primary: - |
|  |  |  |  | Secondary: - |

**Grade evidence table**

| **Outcomes** | **Quality assessment** | | | | | | | **Summary of findings** | | | | | | |
| --- | --- | --- | --- | --- | --- | --- | --- | --- | --- | --- | --- | --- | --- | --- |
|  |  |  |  |  |  |  |  | **No of patients** | | **Effect** | | | **Quality of evidence**  **(GRADE)** | |
|  | **No of participants**  **(studies)** | **Design** | **Risk of bias** | **Inconsistency** | **Indirectness** | **Imprecision** | **Other**  **considerations** | **Permissive hypercapnia in the context of a lung-protective ventilatory strategy and PEEP/FiO_2_ set according to the ARDSnet protocol** | **No permissive hypercapnia in the context of a lung-protective ventilatory strategy and PEEP/FiO_2_ set according to the ARDSnet protocol** | **Relative**  **(95% CI)** | **Risk**  **difference with permissive hypercapnia in the context of a lung-protective ventilatory strategy and PEEP/FiO_2_ set according to the ARDSnet protocol** | **Absolute** |  |  |
| Survival with favorable neurologic/functional outcome at discharge, 30 days, 60 days, 180 days, and/or 1 year; survival only at discharge, 30 days, 60 days, 180 days, and/or 1 year | - | - | - | - | - | - | - | - | - | - | - | - | - |  |

**Consensus of science statement**

In patients with ROSC after PERIOPCA, a lung-protective ventilation strategy (reducing tidal volume, plateau pressure, and driving pressure) and mild hypercapnia (PaCO_2_ of 40-50 mmHg) might be reasonable for improving outcome (COR/LOE: IIb/C-EO).

**References**

None.

**PICO 20**

Among adults with ROSC after cardiac arrest in the perioperative setting (P), does ventilation to a PaO_2_ goal of <200 mmHg (I), compared to PaO_2_ goal of >200 mmHg (C), change survival at discharge, 30days, 60 days, 180 days, and/or 1 year; survival with favorable neurologic/functional outcome at discharge, 30 days, 60 days, 180 days, and/or 1 year (O)?

**Search equation PubMed**

((("heart arrest"[MeSH Terms] OR ("heart"[All Fields] AND "arrest"[All Fields]) OR "heart arrest"[All Fields]) OR ("heart arrest"[MeSH Terms] OR ("heart"[All Fields] AND "arrest"[All Fields]) OR "heart arrest"[All Fields] OR ("cardiac"[All Fields] AND "arrest"[All Fields]) OR "cardiac arrest"[All Fields])) AND (hyperoxemia[All Fields] OR ("hyperoxia"[MeSH Terms] OR "hyperoxia"[All Fields]))) AND (("operating rooms"[MeSH Terms] OR ("operating"[All Fields] AND "rooms"[All Fields]) OR "operating rooms"[All Fields]) OR perioperative[All Fields] OR intraoperative[All Fields])

**Search equation Embase**

('cardiac arrest'/exp OR 'cardiac arrest' OR (cardiac AND ('arrest'/exp OR arrest)) OR 'heart arrest'/exp OR 'heart arrest' OR (('heart'/exp OR heart) AND ('arrest'/exp OR arrest))) AND ('hyperoxia'/exp OR hyperoxia OR 'hyperoxemia'/exp OR hyperoxemia) AND (intraoperative OR 'operating room'/exp OR 'operating room' OR (operating AND room) OR perioperative)

**Search equation Cochrane library**

cardiac arrest OR heart arrest in Title Abstract Keyword AND perioperative OR operating room OR intraoperative in Title Abstract Keyword AND hyperoxia in Title Abstract Keyword - Word variations have been searched

**Study selection**

16 records screened

16 articles discarded (duplicates or excluded after title and abstract evaluation)

0 full-text article excluded due to absence of comparator

0 studies included in qualitative synthesis

**Characteristics of included studies**

| Study | Design | Patients | Interventions | Outcomes |
| --- | --- | --- | --- | --- |
| - | - | - | - | Primary: - |
|  |  |  |  | Secondary: - |

**Grade evidence table**

| **Outcomes** | **Quality assessment** | | | | | | | **Summary of findings** | | | | | | |
| --- | --- | --- | --- | --- | --- | --- | --- | --- | --- | --- | --- | --- | --- | --- |
|  |  |  |  |  |  |  |  | **No of patients** | | **Effect** | | | **Quality of evidence**  **(GRADE)** | |
|  | **No of participants**  **(studies)** | **Design** | **Risk of bias** | **Inconsistency** | **Indirectness** | **Imprecision** | **Other**  **considerations** | **Ventilation to a PaO_2_ goal of <200 mmHg** | **PaO_2_ goal of >200 mmHg** | **Relative**  **(95% CI)** | **Risk**  **difference with ventilation to a PaO_2_ goal of <200 mmHg** | **Absolute** |  |  |
| Survival at discharge, 30days, 60 days, 180 days, and/or 1 year; survival with favorable neurologic/functional outcome at discharge, 30 days, 60 days, 180 days, and/or 1 year | - | - | - | - | - | - | - | - | - | - | - | - | - |  |

**Consensus of science statement**

In patients with PERIOPCA, it may be reasonable to maintain normoxemia and avoid hyperoxemia (PaO_2_ goal of <200 mmHg) in order to improve short and long-term outcome (COR/LOE: IIb/C-EO).

**References**

None.

**PICO 21**

In patients with ROSC after cardiac arrest in the perioperative setting (P), does induction of TTM (target temperature 32-36◦C) for any duration or before some time point (e.g., 1 h after ROSC (I), compared with normothermia (C), change survival with favorable neurologic/functional outcome at discharge, 30 days, 60 days, 180 days, and/or 1 year; survival only at discharge, 30 days, 60 days, 180 days, and/or 1 year (O)?

**Search equation PUBMED**

((((("heart arrest"[MeSH Terms] OR ("heart"[All Fields] AND "arrest"[All Fields]) OR "heart arrest"[All Fields] OR ("cardiac"[All Fields] AND "arrest"[All Fields]) OR "cardiac arrest"[All Fields]) AND ("operating rooms"[MeSH Terms] OR ("operating"[All Fields] AND "rooms"[All Fields]) OR "operating rooms"[All Fields] OR ("operating"[All Fields] AND "room"[All Fields]) OR "operating room"[All Fields])) OR (("heart arrest"[MeSH Terms] OR ("heart"[All Fields] AND "arrest"[All Fields]) OR "heart arrest"[All Fields] OR ("cardiac"[All Fields] AND "arrest"[All Fields]) OR "cardiac arrest"[All Fields]) AND perioperative[All Fields])) OR (("heart arrest"[MeSH Terms] OR ("heart"[All Fields] AND "arrest"[All Fields]) OR "heart arrest"[All Fields] OR ("cardiac"[All Fields] AND "arrest"[All Fields]) OR "cardiac arrest"[All Fields]) AND intraoperative[All Fields])) AND ("hypothermia"[MeSH Terms] OR "hypothermia"[All Fields])) AND ((((("heart arrest"[MeSH Terms] OR ("heart"[All Fields] AND "arrest"[All Fields]) OR "heart arrest"[All Fields] OR ("cardiac"[All Fields] AND "arrest"[All Fields]) OR "cardiac arrest"[All Fields]) AND ("operating rooms"[MeSH Terms] OR ("operating"[All Fields] AND "rooms"[All Fields]) OR "operating rooms"[All Fields] OR ("operating"[All Fields] AND "room"[All Fields]) OR "operating room"[All Fields])) OR (("heart arrest"[MeSH Terms] OR ("heart"[All Fields] AND "arrest"[All Fields]) OR "heart arrest"[All Fields] OR ("cardiac"[All Fields] AND "arrest"[All Fields]) OR "cardiac arrest"[All Fields]) AND perioperative[All Fields])) OR (("heart arrest"[MeSH Terms] OR ("heart"[All Fields] AND "arrest"[All Fields]) OR "heart arrest"[All Fields] OR ("cardiac"[All Fields] AND "arrest"[All Fields]) OR "cardiac arrest"[All Fields]) AND intraoperative[All Fields])) AND ("hypothermia, induced"[MeSH Terms] OR ("hypothermia"[All Fields] AND "induced"[All Fields]) OR "induced hypothermia"[All Fields] OR ("targeted"[All Fields] AND "temperature"[All Fields] AND "management"[All Fields]) OR "targeted temperature management"[All Fields]))

**Search equation EMBASE**

('cardiac arrest'/exp OR 'cardiac arrest' OR (cardiac AND ('arrest'/exp OR arrest)) OR 'heart arrest'/exp OR 'heart arrest' OR (('heart'/exp OR heart) AND ('arrest'/exp OR arrest))) AND ('hypothermia'/exp OR hypothermia OR 'targeted temperature management'/exp OR 'targeted temperature management' OR (targeted AND ('temperature'/exp OR temperature) AND ('management'/exp OR management))) AND (intraoperative OR 'operating room'/exp OR 'operating room' OR (operating AND room) OR perioperative)

**Search equation Cochrane Library**

“cardiac arrest” OR “heart arrest” in Title Abstract Keyword AND “perioperative” OR “operating room” OR “intraoperative” in Title Abstract Keyword AND “hypothermia” OR “induced hypothermia” OR “targeted temperature management” in Title Abstract Keyword - (Word variations have been searched)

**Study selection**

739 records screened

737 articles discarded (duplicates or excluded after title and abstract evaluation)

1 full-text article excluded due to absence of comparator

1 study included in qualitative synthesis

**Characteristics of included studies**

| Study | Design | Patients | Interventions | Outcomes |
| --- | --- | --- | --- | --- |
| Constant et al. | Observational retrospective | Intraoperative cardiac arrest defined as intraoperative loss of a detectable pulse, requiring cardiac compression and/or defibrillation.  The intraoperative period was defined as the time spent in the operating room; this period did not include transport to or stay in the postanesthesia care unit or transport to the ICU | Targeted temperature management 32-34°C for 24 hours | Primary: 1-year functional  outcome as defined by a Cerebral Performance Category (CPC) score of 1 or 2 |
|  |  |  |  | Secondary: proportion  of the adverse events within 72 h after Intraoperative cardiac arrest |

**Grade evidence table**

| **Outcomes** | **Quality assessment** | | | | | | | **Summary of findings** | | | | | | |
| --- | --- | --- | --- | --- | --- | --- | --- | --- | --- | --- | --- | --- | --- | --- |
|  |  |  |  |  |  |  |  | **No of patients** | | **Effect** | | | **Quality of evidence**  **(GRADE)** | |
|  | **No of participants**  **(studies)** | **Design** | **Risk of bias** | **Inconsistency** | **Indirectness** | **Imprecision** | **Other**  **considerations** | **TTM**  **(32-34°C)**  **for 24 hours** | **No TTM** | **Relative**  **(95% CI)** | **Risk**  **difference with TTM**  **(32-34°C)**  **for 24 hours** | **Absolute** |  |  |
| 1-year favorable functional outcome  (CPC 1-2) | 101 (1) | Observational  Retrospective | Serious | No serious | No serious | No serious | None | 14/30 | 32/71 | RR 1.04  (0.65-1.64) | Not significant | AR 0.46 | ⊕⊕⊕🌕  Moderate |  |

**Consensus of science statement**

In comatose patients with PERIOPCA, it may be reasonable to maintain normothermia in order to improve short and long-term outcome (COR/LOE: IIb/C-EO). Potential neurological benefit should be balanced against the hemorrhagic risk related to hypothermia (<37°C) in this surgical setting.

**References**

1. Constant AL, Mongardon N, Morelot Q, Pichon N, Grimaldi D, Bordenave L, Soummer A, Sauneuf B, Merceron S, Ricome S, Misset B, Bruel C, Schnell D, Boisramé-Helms J, Dubuisson E, Brunet J, Lasocki S, Cronier P, Bouhemad B, Carreira S, Begot E, Vandenbunder B, Dhonneur G, Jullien P, Resche-Rigon M, Bedos JP, Montlahuc C, Legriel S. Targeted temperature management after intraoperative cardiac arrest: a multicenter retrospective study. Intensive Care Med 2017;43:485-495.

**PICO 22**

Among adults with ROSC who are treated or not with TTM in the perioperative setting (P), does any clinical variable when abnormal (e.g., clinical exam, EEG, somatosensory evoked potentials [SSEPs], imaging, other) (I), compared with any clinical variable when normal (C), reliably predict death or poor neurologic outcome at discharge, 30 days, 60 days, 180 days, and/or 1year; death only at discharge, 30 days, 60 days, 180 days, and/or 1 year (O)?

**Search equation PUBMED**

(((((((("cardiac arrest"[Title/Abstract]) OR "cardiopulmonary arrest"[Title/Abstract]) OR "circulatory arrest"[Title/Abstract]) OR "heart arrest"[Title/Abstract]))) AND (((("Targeted temperature management"[Title/Abstract]) OR TTM[Title/Abstract]) OR hypothermia[Title/Abstract]) OR "hypothermia protocol"[Title/Abstract])) AND (((((("clinical exam"[Title/Abstract]) OR "somatosensory evoked potentials"[Title/Abstract]) OR SSEPs[Title/Abstract]) OR imaging[Title/Abstract]) OR Electroencephalography[Title/Abstract]) OR EEG[Title/Abstract])) AND (((((((((survival[Title/Abstract]) OR "neurological outcome"[Title/Abstract]) OR "functional outcome"[Title/Abstract]) OR "Hospital discharge"[Title/Abstract]) OR discharge[Title/Abstract])))))

**Search equation EMBASE**

"('cardiac arrest':ti,ab,kw OR 'cardiopulmonary arrest':ti,ab,kw OR 'circulatory arrest':ti,ab,kw OR 'heart arrest':ti,ab,kw) AND ('targeted temperature management':ti,ab,kw OR ttm:ti,ab,kw OR hypothermia:ti,ab,kw OR 'hypothermia protocol':ti,ab,kw) AND ('clinical exam':ti,ab,kw OR 'somatosensory evoked potentials':ti,ab,kw OR sseps:ti,ab,kw OR imaging:ti,ab,kw OR electroencephalography:ti,ab,kw OR eeg:ti,ab,kw) AND (survival:ti,ab,kw OR 'neurological outcome':ti,ab,kw OR 'functional outcome':ti,ab,kw OR 'hospital discharge':ti,ab,kw OR discharge:ti,ab,kw)"

**Search equation Cochrane Library**

“Cardiac arrest” OR “Cardiac arrests” OR “Heart Arrest” OR “Heart Arrests” OR “cardiopulmonary arrest" OR “cardiopulmonary arrests" OR "Cardiopulmonary resuscitation" OR “Cardio Pulmonary resuscitation” in Title Abstract Keyword AND “targeted temperature management” OR “hypothermia” in Title Abstract Keyword AND “clinical exam” OR “somatosensory evoked potentials” OR “sseps” OR “imaging” OR “electroencephalography” OR “ECG” in Title Abstract Keyword - (Word variations have been searched)

**Study selection**

526 records screened

526 articles discarded (duplicates or excluded after title and abstract evaluation)

0 full-text article excluded due to absence of comparator

0 study included in qualitative synthesis

**Characteristics of included studies**

| Study | Design | Patients | Interventions | Outcomes |
| --- | --- | --- | --- | --- |
| - | - | - | - | Primary: - |
|  |  |  |  | Secondary: - |

**Grade evidence table**

| **Outcomes** | **Quality assessment** | | | | | | | **Summary of findings** | | | | | | |
| --- | --- | --- | --- | --- | --- | --- | --- | --- | --- | --- | --- | --- | --- | --- |
|  |  |  |  |  |  |  |  | **No of patients** | | **Effect** | | | **Quality of evidence**  **(GRADE)** | |
|  | **No of participants**  **(studies)** | **Design** | **Risk of bias** | **Inconsistency** | **Indirectness** | **Imprecision** | **Other**  **considerations** | **Abnormal clinical variable** | **Normal clinical variable** | **Relative**  **(95% CI)** | **Risk**  **difference with abnormal clinical variable** | **Absolute** |  |  |
| Prediction of death or poor neurologic outcome at discharge, 30 days, 60 days, 180 days, and/or 1year; death only at discharge, 30 days, 60 days, 180 days, and/or 1 year | – | - | - | - | - | - | - | - | - | - | - | - | - |  |

**Consensus of science statement**

In patients with PERIOPCA and ROSC, it may be reasonable to use a multimodal strategy for prognostication, giving emphasis on allowing sufficient time for neurological recovery and to enable sedatives/paralytics to be cleared (COR/LOE: IIb/C-EO).

**References**

None.
