# Supplementary material for: Clinical practice recommendations on the management of perioperative cardiac arrest: A report from the PERIOPCA Consortium
Source: Crit Care. 2021 Jul 29;25:265. doi: 10.1186/s13054-021-03695-2 (PMC8323279; doi:10.1186/s13054-021-03695-2)
Supplement: Supplementary file 4 — Additional file 4: The full list of PERIOPCA recommendations, including a short introduction, consensus on science, treatment recommendations, and values, preferences, and task force insights. [file 13054_2021_3695_MOESM4_ESM.docx]

**PERIOPCA RECOMMENDATIONS**

**PICO 1**

*Among adults who are in cardiac arrest in the perioperative setting (P), does any ETCO_2_ level value, when present (I), compared with any ETCO_2_ level below that value (C), change survival with favorable neurologic/functional outcome at discharge, 30 days, 60 days, 180 days, and/or 1 year; survival only at discharge, 30 days, 60 days, 180 days, and/or 1 year; ROSC (O)?*

**Introduction**

End-tidal carbon dioxide (ETCO_2_) has been reported to correlate with systemic blood flow and outcome of CPR. Various studies demonstrated that under constant ventilation and carbon dioxide production, an abrupt increase in ETCO_2_ during CPR provides the fastest indication of ROSC (1-6). End-tidal carbon dioxide changes may precede a palpable pulse, and changes and trends in ETCO_2_ values during CPR may be more important than absolute ETCO_2_ levels. However, despite the abundant human and animal studies supporting the usefulness of ETCO_2_, its optimal values during CPR remain controversial and it is therefore necessary to understand the limitation of using any absolute value of ETCO_2_ to predict the prognosis as the cause of PERIOPCA may significantly affect the ETCO_2_ values, particularly if the cause is related to pulmonary function.

**Consensus on science**

We did not identify any evidence to address the critical outcomes of survival with favorable neurologic/functional outcome at discharge, 30 days, 60 days, 180 days, and/or 1 year; survival only at discharge, 30 days, 60 days, 180 days, and/or 1 year; and ROSC.

**Treatment recommendations**

In patients with PERIOPCA, it may be reasonable to maintain an ETCO_2_ ≥ 10 mmHg during advanced life support. However, ETCO_2_ should be evaluated in the context of the patient’s clinical status and individualized targets may be necessary considering the cause of arrest, the degree of hypoxia, the quality of CPR and time to ROSC (COR/LOE: IIb/C-EO).

**Values, Preferences, and Task Force Insights**

In order to make this recommendation, the working group was keen to acknowledge that various causes affect oxygenation and ETCO_2_ levels during the intraoperative period (7,8). Low ETCO_2_ values are observed in low-flow states, supraglottic airways, increased airway resistance, pulmonary edema, and hyperventilation (9). However, hypercapnia is associated with increases in pulmonary vascular resistance and right ventricular afterload, cerebral vasodilation, and negative inotropism, which may necessities the application of increased respiratory rates (10). In consideration of the clear benefits of high-quality CPR, chest compressions in these patients may be tailored to ETCO_2_ in order to promote effective blood flow and organ perfusion. Further research is necessary to evaluate the role of ETCO_2_ as a mean of discriminating patients with poor prognosis or to assess the role of ETCO_2_ during CPR in pulmonary causes of cardiac arrest.

**PICO 2**

*Among adults who are in cardiac arrest in the perioperative setting (P), does the use of physiological feedback regarding CPR quality (e.g., arterial lines, ETCO_2_ monitoring, SpO_2_ waveforms, or others) (I), compared with no feedback (C), change survival with favorable neurologic/functional outcome at discharge, 30 days, 60 days, 180 days, and/or 1 year; survival only at discharge, 30 days, 60 days, 180 days, and/or 1 year; ROSC; change in physiologic values by modifications in CPR (O)?*

**Introduction**

Resuscitation guidelines remain uniform across all cardiac arrest patients, focusing on the delivery of chest compressions to a standardized rate and depth and algorithmic vasopressor dosing (11). However, this approach does not incorporate a patient's individualized response into ongoing resuscitative efforts (12). Physiologic monitoring of CPR is gaining in importance, as it provides a real-time window into the cellular physiology of patients. The American Heart Association (AHA) recommends monitoring CPR quality using ETCO_2_ or invasive hemodynamic data (13). Considering that adequate coronary perfusion pressure is essential for establishing ROSC, providing physiology-targeted CPR may optimize the resuscitation efforts and improve outcomes.

**Consensus on science**

For the important outcome of survival to discharge and survival to discharge with good functional outcomes, low-quality evidence (downgraded for risk of bias) suggest that physiological feedback during CPR may be used to facilitate and/or titrate the resuscitation efforts (14-16).

**Treatment recommendations**

In adults with cardiac arrest in the perioperative setting, the use of physiological feedback may be reasonable to increase CPR quality and improve short- and long-term outcome (COR/LOE: IIb/C-EO).

**Values, Preferences, and Task Force Insights**

In making these recommendations, we considered the lack of specific randomized trials or meta-analyses comparing physiology-guided CPR to standard CPR in the perioperative setting. Cardiac arrest may be anticipated in deteriorating patients or may have a sudden onset. As most patients are highly monitored, physiology-guided CPR could serve as a method for improving tissue perfusion and oxygenation in patients with PERIOPCA.

**PICO 3**

*Among adults who are in ventricular fibrillation or pulseless ventricular tachycardia in the perioperative setting (P), does any interval of CPR first (e.g., 2 min) (I), compared with defibrillation first (C), change survival with favorable neurological/functional outcome at discharge, 30 days, 60 days, 180 days and/or 1 year, survival only at discharge, 30 days, 60days, 180 days and/or 1 year, ROSC, termination of arrhythmia (O)?*

**Introduction**

In the early minutes of cardiac arrest with a shockable rhythm, timing of defibrillation and CPR is debated. According to the “3-phase” model of cardiac arrest, defibrillation success is related to the energy status of the heart (17). In this model, the first 4 minutes of ventricular fibrillation cardiac arrest (electrical phase) are associated with increased likelihood for ROSC after defibrillation attempt. Between 4 and 10 minutes (circulatory phase), energy stores of myocardium are severely depleted. Beyond 10 minutes (metabolic phase), survival rates are abysmal, with no therapy yet identified producing clinical utility.

**Consensus on science**

A prospective multicenter study reported that survival to hospital discharge was higher in patients with time to first shock less than 3 minutes [38% (1037 of 2714) vs. 21% (107 of 500), p<0.001] (18). In another prospective multicenter registry trial that included 3.276 patients, the authors showed a higher rate of survival to discharge in patients defibrillated during first 2 minutes [1863/4744 (39.3) vs. 455/2045 (22.2), p<0.001] (19). This was also associated with higher rates of ROSC, survival at 24h, and neurologic status at discharge. Also, patients with ventricular fibrillation/pulseless ventricular tachycardia lasting more than 3-4 minutes may benefit from CPR. Also, a clear association has been reported between early defibrillation of ventricular fibrillation/pulseless ventricular tachycardia and survival to discharge (20). Another option to improve survival in this population may be the use of automatic external defibrillators (AED). A retrospective observational study reported a higher rate of ROSC [modified stacked shock (90%) vs. initial chest compression (56%), p<0.05] and survival to discharge in monitored ventricular fibrillation/pulseless ventricular tachycardia patients when using expeditious (stacked shocks protocol) defibrillation [modified stacked shock (71%), stacked shock (58%) vs. initial chest compression (18%), p<0.001] (21). However, in another observational trial including hospitalized patients with cardiac arrest, use of AEDs was not associated with improved survival [38.4% vs. 39.8%; RR, 1.00; 95% CI, 0.88-1.13, p<0.001] (22).

**Treatment recommendations**

In adult patients with PERIOPCA, ventricular fibrillation/pulseless ventricular tachycardia should be defibrillated within 3 minutes after the onset of the arrest (COR/LOE: I/C-LD). The use of AEDs in patients with ventricular fibrillation/pulseless ventricular tachycardia can be useful for improving survival (COR/LOE: IIa/C-LD). It is not recommended to defibrillate patients with ventricular fibrillation/pulseless ventricular tachycardia lasting more than 3 minutes without prior chest compressions (COR/LOE: III/C-LD).

**Values, Preferences, and Task Force Insights**

In making these recommendations, we considered the lack of randomized trials or meta-analyses comparing two different approaches (defibrillation first or chest compression before defibrillation) in patients with ventricular fibrillation/pulseless ventricular tachycardia. Due to the relatively low rate of cardiac arrest during the perioperative period, it is difficult to conduct controlled studies on this subject. Therefore, it remains unclear whether these options offer any advantage to patients with perioperative ventricular fibrillation/pulseless ventricular tachycardia cardiac arrest.

**PICO 4**

*Among adults who are in cardiac arrest in the perioperative setting (P), does early epinephrine delivery by IV or IO route (e.g., less than 10 min after the beginning of resuscitation) (I), compared with delayed timing of epinephrine delivery (e.g., more than 10 min after the beginning of resuscitation) (C), change survival with favorable neurologic/functional outcome at discharge, 30 days, 60 days, 180 days, and/or 1 year; survival only at discharge, 30 days, 60 days, 180 days, and/or 1 year; ROSC (O)?*

**Introduction**

Epinephrine administration may increase ROSC rates and short-term survival, but it is not clear if it is associated with long-term survival and survival with a favorable neurological outcome (23). Although epinephrine does not increase ROSC rates during the first 2 min of cardiac arrest (24), it may have an important role in prolonged CPR, during which significant changes in metabolic and physiological profiles occur. Indeed, vasopressors are recommended during the circulatory and metabolic phases (> 4 min) (17), which are characterized by a decrease in adenosine triphosphate levels (25). Although time to drug administration is shorter in the hospital compared to out-of-hospital settings, it remains unknown if early administration is better than late administration in patients with PERIOPCA.

**Consensus on science**

One observational study reported that early epinephrine delivery by intravenous or intraosseous route reduces survival to discharge with good functional outcome [24.7% (357 of 1445) vs. 40.8% (567 of 1389), p<0.001] (26). Two observational studies reported that early epinephrine delivery by intravenous or intraosseous route may result in reduction in survival to discharge [13% (4588 of 35272) vs. 15.7% (2203 of 14012), p<0.001] (26,27). Also, one observational study reported that early epinephrine delivery by intravenous or intraosseous route resulted in slight reduction in ROSC rates [74% (1118 of 1510) vs. 79.1% (1158 of 1464, p<0.001] (27).

**Treatment recommendations**

In adult patients with PERIOPCA, epinephrine administration after the 3^rd^ shock can be beneficial (COR/LOE: IIa/C-LD).

**Values, Preferences, and Task Force Insights**

In making these recommendations, we considered the lack of randomized trials or meta-analyses comparing early to late epinephrine administration, as well as the underlying pathophysiology including the 3-phase cardiac arrest model described by Weisfeldt and Becker (17,28,29). In the perioperative setting, the time-sensitive changes in the physiology of patients with PERIOPCA may be delayed due to immediate recognition of cardiac arrest and the early onset of CPR/defibrillation. It remains unclear whether a hemodynamically-guide epinephrine administration offer any advantage to patients with PERIOPCA.

**PICO 5**

*In adult patients in cardiac arrest in the perioperative setting (P), does low-dose epinephrine (< 1 mg) or high-dose epinephrine (> 1mg) (I), compared with standard-dose epinephrine (1 mg bolus dose) (C), change survival to 180 days with good neurologic outcome, survival to 180 days, survival to hospital discharge with good neurologic outcome, survival to hospital discharge, ROSC (O)?*

**Introduction**

Although epinephrine has been the drug of choice in CPR for more than 100 years, the currently recommended dose (1 mg) is based on experimental data and has not be adequately studied in the clinical setting (30). Until now, several human studies have evaluated higher doses of epinephrine in human cardiac arrest without differences in survival or hospital discharge (31-34). However, epinephrine administration may result in several deleterious effects, especially during the post-resuscitation period. The recommended dose for epinephrine by both the AHA and the ERC is 0.014 mg/kg (1 mg for a 70-kg adult) every 3 to 5 minutes during cardiac arrest (35). However, it has become evident that high doses of epinephrine may further increase coronary perfusion pressure, but fail to improve long-term survival and neurologic outcome (36). Furthermore, even higher doses seem to increase the in-hospital mortality rate, while its cumulative dose (4 mg vs. 1 mg) is an independent predictor of poor neurologic outcome as well (35,37).

**Consensus on science**

We did not identify any evidence to address the critical outcomes of survival to 180 days with good neurologic outcome, survival to 180 days, survival to hospital discharge with good neurologic outcome, survival to hospital discharge, and ROSC.

**Treatment recommendations**

In patients with PERIOPCA, it may be reasonable to administer 1 mg epinephrine for improving coronary perfusion pressure (COR/LOE: IIb/C-EO).

**Values, Preferences, and Task Force Insights**

In order to make this recommendation, the working group acknowledges the lack of studies in the perioperating setting, as well as the discrepancies between laboratory and clinical CPR research. Due to its pharmacological actions, epinephrine is considered as an agent resembling a double-edged sword with potent vasoconstrictive properties essential for improving coronary perfusion pressure on the one side and deleterious adverse effects negatively influencing survival on the other side. The working group makes this recommendation highlighting that epinephrine is one of the many interventions that need to be appropriately integrated into the series of essential steps for optimal CPR.

**PICO 6**

*Among adults who are in cardiac arrest in in the perioperative setting (P), does avoiding the use of any vasopressor during CPR (I), compared with using epinephrine alone or vasopressin alone, or vasopressin in combination with epinephrine (C), change survival with favorable neurologic/functional outcome at discharge, 30 days, 60 days, 180 days, and/or 1 year; survival only at discharge, 30 days, 60 days, 180 days, and/or 1 year (O)?*

**Introduction**

Perioperative cardiac arrest occurs due to cardiac and non-cardiac causes, including hypovolemia, vasodilation, hypoxia, acidosis, electrolyte disturbances, nerve reflex, drug usage, or anesthesia (38). As a result, the patient may experience any phase of cardiac arrest (17). During the electrical phase, the fibrillating myocardium has used up all its energy stores and catecholamine levels are extremely elevated, while the adrenoreceptors are possibly already maximally stimulated (39,40). Therefore, exogenous vasopressor and specifically epinephrine will be ineffective at this time, exerting detrimental effects only. During the hemodynamic phase, generation of adequate coronary and cerebral perfusion pressure is critical and during this phase, evidence supports administration of vasopressors. However, during the metabolic phase, prolonged ischemia, in association with epinephrine-induced vasoconstriction and endotoxin release in the circulation, may result in severe organ injury. From the onset of cardiac arrest and irrespectively to the cardiac arrest phase, the human body is under severe acidosis which further aggravates with time. The acidosis may reduce the effectiveness of vasopressors, leading to the false need for multiple repetitive doses (41, 42).

**Consensus on science**

We did not identify any evidence to address the critical outcomes of survival with favorable neurologic/functional outcome at discharge, 30 days, 60 days, 180 days, and/or 1 year; survival only at discharge, 30 days, 60 days, 180 days, and/or 1 year.

**Treatment recommendations**

In patients with PERIOPCA, it may be reasonable to administer epinephrine every 3 to 5 minutes (COR/LOE: IIb/C-EO).

**Values, Preferences, and Task Force Insights**

In order to make this recommendation, the working group acknowledges the lack of studies in the perioperating setting. Epinephrine is still the recommended vasopressor agent of the International Liaison Committee on Resuscitation and both the ERC and AHA. It may be essential for improving coronary perfusion pressure, but may be also associated with numerous adverse effects. In consideration of the possible benefits of increasing systemic vascular resistance, limiting epinephrine dose may be useful for improving hemodynamics during CPR. Future research should be oriented toward different doses and options, examining the mode of action of different agents, possible drug combinations, and the appropriate timing for their administration during CPR.

**PICO 7**

*Among adults who are in cardiac arrest in the perioperative setting (P), does administration of antiarrhythmic drugs (e.g., amiodarone, lidocaine, other) (I), compared with not using antiarrhythmic drugs (no drug or placebo) (C), change survival with favorable neurologic/functional outcome at discharge, 30 days, 60 days, 180 days, and/or 1 year; survival only at discharge, 30 days, 60 days, 180 days, and/or 1 year; ROSC (O)?*

**Introduction**

Antiarrhythmic medications are commonly administered during and immediately after a ventricular fibrillation/pulseless ventricular tachycardia cardiac arrest. The primary objective of antiarrhythmics is to facilitate successful defibrillation and to reduce the risk of recurrent arrhythmias. According to the updated AHA guidelines (43), it is recommended to use either amiodarone or lidocaine for shock-refractory ventricular fibrillation/pulseless ventricular tachycardia and magnesium for torsades de pointes.

**Consensus on science**

Intravenous amiodarone has been shown to improve survival to hospital admission in patients with out-of-hospital cardiac arrest due to refractory ventricular arrhythmia when compared with placebo or lidocaine (43,44). Some authors reported no benefit of using amiodarone instead of lidocaine among cardiac arrest patients regarding to ROSC [66.7% (95% CI 50% - 80%) vs. 83% (95% CI 72% - 91%), p=0.07] and survival to discharge [36.1% (95% CI 23% - 53%) vs. 55.9% (95% CI 43% - 68%), p=0.06] (45). Another retrospective trial failed to show superiority of amiodarone vs. lidocaine-amiodarone combination or lidocaine alone in patients with ventricular fibrillation/pulseless ventricular tachycardia [survival to 24h: 55 (34%) vs. 63 (43%) vs. 50 (17%), p=0.39; survival to discharge: 39 (29%) vs. 45 (36%) vs. 42 (17%), p=0.72] (46). A prospective, randomized trial reported no difference between magnesium and placebo use in cardiac arrest patients regarding ROSC [41 (54%) vs. 48 (60%), p=0.44], survival to 24h [33 (43%) vs. 40 (50%), p=0.41], and survival to hospital discharge [16 (21%) vs. 17 (21%), p=0.98] (47). Intravenous nifekalant, a pure class III anti-arrhythmic drug, has been approved only in Japan for the treatment of life-threatening ventricular fibrillation/pulseless ventricular tachycardia in patients with structural heart disease who are resistant to other anti-arrhythmic drugs. Nifekalant use was associated with higher rates of ROSC [23 vs. 15 (1.4-18.2) p=0.01] and termination of shock-resistant ventricular fibrillation/pulseless ventricular tachycardia with/without additional shock compared to lidocaine [11 vs. 2 (1.8-45.6) p=0.003, 22 vs. 15 (1.1-13.0), p=0.03], with no differences in survival to discharge and 1-month survival (48).

**Treatment recommendations**

In adult patients with PERIOPCA, it is recommended to administer amiodarone or lidocaine for the treatment of ventricular fibrillation/pulseless ventricular tachycardia (COR/LOE: I/C-LD). Magnesium is not indicated for the treatment of ventricular fibrillation/pulseless ventricular tachycardia in the perioperative setting (COR/LOE: III/C-LD).

**Values, Preferences, and Task Force Insights**

In order to make this recommendation, the working group was keen to acknowledge the lack of randomized trials in the perioperative setting. Limited data comes from few retrospective and small single center trials with mixed in-hospital population. Among the aforementioned antiarrhythmic drugs, amiodarone is the most commonly used drug in the perioperative setting. The majority of evidence regarding the use of antiarrhythmic drugs comes from trials with out-hospital cardiac arrest. This evidence need to be tested in perioperative settings, where the time to antiarrhythmic administration is considerably shorter, given the earlier response times, the preexisting vascular access, and the larger number of trained providers.

**PICO 8**

*Among adults who are in cardiac arrest in the perioperative setting (P), does early antiarrhythmic (e.g., amiodarone, lidocaine, other) delivery by IV or IO route (e.g., less than 6 min after the beginning of resuscitation) (I), compared with delayed timing of antiarrhythmic delivery (e.g., more than 6 min after the beginning of resuscitation) (C), change survival with favorable neurologic/functional outcome at discharge, 30 days, 60 days, 180 days, and/or 1 year; survival only at discharge, 30 days, 60 days, 180 days, and/or 1 year; ROSC (O)?*

**Introduction**

The 2018 AHA focused update recommends either amiodarone or lidocaine for ventricular fibrillation/pulseless ventricular tachycardia that is unresponsive to defibrillation. Administration of antiarrhythmics nay be more beneficial in patients with witnessed arrest, considering the shorter time to drug administration. However, there are very few randomized controlled trials conducted with amiodarone and lidocaine and none of these trials demonstrated significant difference in the improvement of survival to hospital discharge (44,49,50). Moreover, these trials did not assess the impact of timing or sequence of amiodarone vs. epinephrine in the in-hospital setting.

**Consensus on science**

We did not identify any evidence to address the critical outcomes of survival with favorable neurologic/functional outcome at discharge, 30 days, 60 days, 180 days, and/or 1 year; survival only at discharge, 30 days, 60 days, 180 days, and/or 1 year; and ROSC.

**Treatment recommendations**

In adult patients with perioperative ventricular fibrillation/pulseless ventricular tachycardia, it might be reasonable to administer amiodarone or lidocaine after the 3^rd^ shock (COR/LOE: IIb/C-EO).

**Values, Preferences, and Task Force Insights**

In order to make this recommendation, the working group was keen to acknowledge the considerable controversy in the literature. In other settings, ventricular fibrillation/pulseless ventricular tachycardia is regarded as the most treatable cardiac arrest rhythm despite the fact that most defibrillation attempts do not result in sustained ROSC (51-53). Whether these medications have any effect on overall outcomes remains unknown and there is no evidence regarding the optimal timing (early or delayed) of antiarrhythmics administration in the perioperative setting.

**PICO 9**

*Among adults with cardiac arrest with a secure airway receiving chest compressions (in the perioperative setting, and with standard tidal volume) (P), does a ventilation rate of 10 breaths/min (I), compared with any other ventilation rate (C), change survival with favorable neurologic/functional outcome at discharge, 30 days, 60 days, 180 days, and/or 1 year; survival only at discharge, 30 days, 60 days, 180 days, and/or 1 year; ROSC (O)?*

**Introduction**

Although ventilation is important for optimizing oxygen delivery, hyperventilation during CPR may increase intrathoracic pressure, decreasing venous return and the compression-related cardiac output (54-56). Most patients in the operating room are intubated and mechanically ventilated, but the optimal ventilation rate in case of PERIOPCA remains uncertain. In the 2015 guidelines, very-low-quality evidence did not allow to estimate the effect of a ventilation rate of 10/min compared with any other rate for the important outcome of ROSC (57).

**Consensus on science**

We did not identify any evidence to address the critical outcomes of survival with favorable neurologic/functional outcome at discharge, 30 days, 60 days, 180 days, and/or 1 year; survival only at discharge, 30 days, 60 days, 180 days, and/or 1 year; and ROSC.

**Treatment recommendations**

In adult patients with PERIOPCA and a secure airway, a ventilation rate of 10 breaths/min during CPR may be reasonable (COR/LOE: IIb/C-EO).

**Values, Preferences, and Task Force Insights**

In order to make this recommendation, the working group acknowledges the lack of studies in the perioperating setting. Two observational studies (58,59) and one prospective interventional trial (60) reported ventilation rates during CPR in patients with in-hospital cardiac arrest but excluded patients in the operating room environment. Yeung et al. (61) reported ventilation rate in a population that included both out- and in- hospital cardiac arrest. Edelson et al. (62) reported ventilation rates during CPR in patients with in-hospital cardiac arrest but did not report any outcome. High-quality chest compressions remain the most important parameter for increasing the possibility of ROSC and improving survival. In patients with prolonged CPR or in those with hypoxemia, effective ventilation is paramount for allowing adequate carbon dioxide removal and providing sufficient arterial oxygen content while minimizing the risk of impairing circulation. However, the ideal ventilation parameters during CPR remain unknown.

**PICO 10**

*Among adults who are in cardiac arrest due to pulmonary embolism or suspected pulmonary embolism in the perioperative setting (P), does any specific alteration in treatment algorithm (e.g., fibrinolytics, or any other) (I), compared with standard care (C), change survival with favorable neurologic/functional outcome at discharge, 30 days, 60 days, 180 days, and/or 1 year; survival only at discharge, 30 days, 60 days, 180 days, and/or 1 year; ROSC (O)?*

**Introduction**

Massive pulmonary embolism is a serious clinical problem in the perioperative setting and is responsible for 150,000-200,000 deaths per year in the United States (63). All perioperative patients, especially those with trauma or who undergo prostate or orthopedic surgery, are at increased risk of venous thromboembolism. Malignancy, immobility, obesity, smoking, oral contraceptives, hormone replacement therapy, and antipsychotic medications have been associated with the highest risk (64). Clinical outcomes have been shown to improve after prompt recognition and aggressive intervention. However, the diagnosis is difficult and clinical history and assessment, capnography, and echocardiography should be timely used for its confirmation.

**Consensus on science**

We did not identify any evidence to address the critical outcomes of survival with favorable neurologic/functional outcome at discharge, 30 days, 60 days, 180 days, and/or 1 year; survival only at discharge, 30 days, 60 days, 180 days, and/or 1 year; and ROSC.

**Treatment recommendations**

In adult patients with PERIOPCA due to pulmonary embolism or suspected pulmonary embolism, early consideration of thrombolysis and CPR duration of at least 60-90 minutes with or without the use of a mechanical chest compression device may be reasonable before terminating resuscitation attempts (COR/LOE: IIb/C-LD). The emergency treatment option among fibrinolytic therapy, surgical, or mechanical thrombectomy should be selected based on timing and available expertise, since no clear benefit of one approach over the other has been demonstrated.

**Values, Preferences, and Task Force Insights**

Although fibrinolytics may increase the rate of ROSC, survival to discharge, and long-term neurological function (65), a subgroup analysis of patients treated with thrombolytics compared with placebo did not prove survival difference (66). However, the study suffered by methodological limitations and was not powered to reach significance (66). Thrombolytics have been repeatedly used in the treatment of cardiac arrest due to acute pulmonary embolism, but their effectiveness in improving neurologically intact survival remains unknown (67,68).

In patients with cardiac arrest due to pulmonary embolism, the risk of fibrinolysis-associated major bleeding seems relatively low (66,69). Indeed, in the Thrombolysis in Cardiac Arrest (TROICA) study, major bleeding complications were not significantly higher in the thrombolysis group [symptomatic intracranial hemorrhage, 0.8% vs. 0%; RR, 8.93 (95% CI, 0.48-165.45); p=0.13; major non-intracranial hemorrhage, 7.7% vs. 6.4; RR, 1.21 (95% CI, 0.77-1.88); p=0.48; ischemic stroke, 0.8% vs. 0.6%; RR, 1.32 (95% CI, 0.30-5.88); p=1.00] (66). Also, recent evidence suggest that thrombolysis in cardiac arrest patients with confirmed pulmonary embolism is not associated with increased mortality due to hemorrhage compared to the control group (6% vs. 5%; p=0.73). The benefits of fibrinolysis seem to outweigh the potential bleeding complications of the treatment. The optimal fibrinolytic drug and dosing regimen for cardiac arrest due to pulmonary embolism in the perioperative setting should be further investigated.

**PICO 11**

*Among pregnant women who are in cardiac arrest in the perioperative setting (P), do any specific interventions (I), compared with standard care (usual resuscitation practice) (C), change survival with favorable neurologic/functional outcome at discharge, 30 days, 60 days, 180 days, and/or 1 year; survival only at discharge, 30 days, 60 days, 180 days, and/or 1 year; ROSC (O)?*

**Introduction**

Cardiac arrest during pregnancy is one of the most difficult scenarios for both the mother and the healthcare personnel. Cardiac arrest occurs in 1:12000 admissions for delivery in the US, while globally, 800 maternal deaths occur daily (70). The physiological characteristics of the pregnant woman makes the resuscitation process uniquely different and challenging. The most obvious difference is that there are two patients, the mother and the fetus, while survival depends on the underlying etiology of cardiac arrest (71).

**Consensus on science**

We did not identify any evidence to address the critical outcomes of survival with favorable neurologic/functional outcome at discharge, 30 days, 60 days, 180 days, and/or 1 year; survival only at discharge, 30 days, 60 days, 180 days, and/or 1 year; and ROSC.

**Treatment recommendations**

In pregnant women with PERIOPCA, the effectiveness of any special interventions, compared to standard measures, is uncertain, except probably for manual uterine displacement during chest compressions (COR/LOE: IIb/C-EO). In pregnant women with PERIOPCA due to suspected or proven pulmonary embolism, it may be reasonable to use thrombolysis or other measures to remove clot (e.g., surgical or percutaneous pulmonary embolectomy) (COR/LOE: IIb/C-EO). Extracorporeal membrane oxygenation may be considered as an acceptable salvage therapy for pregnant and postpartum patients with PERIOPCA or those with critical cardiac or pulmonary illness (COR/LOE: IIb/C-EO).

**Values, Preferences, and Task Force Insights**

In order to make this recommendation, the working group acknowledges the lack of studies. To date, no randomized trials evaluating the effect of specialized interventions vs. standard care in cardiac arrest associated with pregnancy have been published. However, the gravid uterus may cause aortocaval compression and decrease venous return. Therefore, a left lateral tilt from the horizontal position and/or placing a wedge under the woman’s right side, or manual displacement of the uterus to the left may effectively relieve aortocaval compression. Of note, about 1 in 1000-3000 pregnancies are complicated by pulmonary embolism and about one fourth of women presenting with signs of pulmonary embolism suffers cardiac arrest (72). The management of massive pulmonary embolism in the non-pregnant adult relies on the acute removal of the thrombotic clot, most often by thrombolysis. However, the benefit and risk of thrombolysis in pregnant women with pulmonary embolism and cardiac arrest remain unknown. Published cases of thrombolysis during pregnancy and the postpartum period suggest a high maternal and fetal survival (73,74). Interestingly, data from single-center observational studies report a survival rate for the mother and fetus of 60%-88.9% and 77.8%, respectively, when extracorporeal membrane oxygenation was initiated during pregnancy, childbirth, or postpartum (75,76). In young and healthy population, extracorporeal membrane oxygenation may increase the survival rates and has been considered a salvage therapy for peripartum women with reversible forms of cardiorespiratory failure (77,78). High-quality chest compressions and manual uterine displacement remain the most important parameters for increasing the possibility of ROSC and improving survival. Further high-quality studies are necessary to assess the effectiveness of specific interventions in pregnant women in the perioperative setting.

**PICO 12**

*Among adults who are in cardiac arrest or respiratory arrest due to opioid toxicity in the perioperative setting (P), does any specific therapy (e.g., naloxone, bicarbonate, or other drugs) (I), compared with usual ALS (C), change survival with favorable neurologic/functional outcome at discharge, 30 days, 60 days, 180 days, and/or 1 year; survival only at discharge, 30 days, 60 days, 180 days, and/or 1 year; ROSC (O)?*

**Introduction**

Opioids are the mainstay of nociceptive pain treatment, but they can increase the risk of respiratory depression through mu receptor agonism, especially in fragile patients or patients undergoing major surgery (79). Untreated severe respiratory depression leads to increasing hypoxia and hypercapnia with progressive hemodynamic deterioration and, eventually, asphyxial cardiac arrest (80).

**Consensus on science**

We did not identify any evidence to address the critical outcomes of survival with favorable neurologic/functional outcome at discharge, 30 days, 60 days, 180 days, and/or 1 year; survival only at discharge, 30 days, 60 days, 180 days, and/or 1 year; and ROSC.

**Treatment recommendations**

In patients with PERIOPCA due to opioid toxicity, it might be reasonable to administer specific agents in addition to advanced life support (COR/LOE: IIb/C-EO).

**Values, Preferences, and Task Force Insights**

We found no evidence supporting that any specific therapy, compared with standard advanced life support, changes ROSC rates or survival with favorable neurologic/functional outcome among adults with PERIOPCA due to opioid toxicity. Naloxone is a pure opioid receptor antagonist and it is widely used to antagonize opioid-induced respiratory depression and prevent cardiac arrest (81). Preclinical data suggest that naloxone could exert inotropic and antiarrhythmic effects, but the available results are conflicting (82-86). A retrospective cohort study of patients with cardiac arrest due to suspected opioid overdose reported change of cardiac rhythm in 42% of those who received naloxone (87). However, that study had serious limitations and showed no effect on clinical outcomes (87). Considering that opioid-induced cardiac arrest is caused by hypoxia and that no cardiotonic effect of naloxone has been actually demonstrated, the use of naloxone as antidote in these patients seems reasonable (88). Nevertheless, it remains unclear whether the antidotes used in clinical practice can improve the outcome of opioid-induced PERIOPCA.

**PICO 13**

*Among adults who are in cardiac arrest in the perioperative setting (P), does corticosteroid or mineralocorticoid administration during and/or after CPR or the combined use of vasopressin, epinephrine, and steroids during and/or after CPR (I), compared with not using steroids or epinephrine alone during CPR and no steroids after CPR (C), change survival with favorable neurologic/functional outcome at discharge, 30 days, 60 days, 180 days, and/or 1 year; survival only at discharge, 30 days, 60 days, 180 days, and/or 1 year; ROSC (O)?*

**Introduction**

Emerging evidence demonstrates that the hypothalamic-pituitary-adrenal axis may play an important role in cardiac arrest and CPR due to the ischemia-induced adrenal sufficiency (89,90). In addition, low cortisol levels may aggravate post-resuscitation hemodynamics and are correlated with poor outcomes (91). Corticosteroid therapy could theoretically confer benefit to the outcomes of cardiac arrest patients, but its safety remains vague. Some previous trials with glucocorticoid administration have shown significant benefits (92,93); however, some other reports have showed ineffectiveness in terms of survival rates and neurological recovery (94,95).

**Consensus on science**

Two randomized controlled trials reported that the use of corticosteroid or mineralocorticoid or the combined use of vasopressin, epinephrine, and steroids during/after CPR results in an increase in ROSC [83.1 (148 of 178) vs. 62.1 (118 of 190), p<0.001] (96,97). In addition, these trials showed that the use of corticosteroid or mineralocorticoid or the combined use of vasopressin, epinephrine, and steroids during/after CPR probably results in a large increase in survival to discharge [18.8% (9 of 48) vs. 3.8% (2 of 52, p = 0.039] and survival to discharge with good functional outcomes [13.8% (18 of 130) vs. 5.1 (7 of 138), p = 0.029] (96,97).

**Treatment recommendations**

In adult patients with PERIOPCA, it is reasonable to administer corticosteroid or mineralocorticoid or the combination of vasopressin, epinephrine, and steroids during/after CPR to increase ROSC (COR/LOE: IIa/B-R). In these patients, these drugs can be useful for improving survival to discharge with good functional outcome (COR/LOE: IIa/B-R).

**Values, Preferences, and Task Force Insights**

Although the inherent heterogeneity of the available studies does not allow for reaching a definitive conclusion as to the efficacy of corticosteroid therapy in other settings, the evidence favor its use in patients with PERIOPCA. This is very important because their inexpensive nature and wide availability facilitate their use during the perioperative period. Corticosteroid therapy may also improve outcome if combined with vasopressin and epinephrine. The evidence regarding the administration of corticosteroid therapy alone are scarce and cannot support their use as a monotherapy. Nevertheless, corticosteroids are potent immunosuppressive agents and may increase the possibilities of infection and sepsis (98). In addition, they may increase the risk of adverse gastrointestinal effects (99). In consideration of the clear benefits, steroid therapy combined with vasopressin and epinephrine rather than steroids alone may be administered after PERIOPCA.

**PICO 14**

*In adult patients with cardiac arrest due to suspected drug toxicity (e.g., local anesthetics, tricyclic antidepressants, others) in the perioperative setting (P), does administration of IV lipid (I), compared with no IV lipid (C), change survival with favorable neurologic/functional outcome at discharge, 30 days, 60 days, 180 days, and/or 1 year; survival only at discharge, 30 days, 60 days, 180 days, and/or 1 year; ROSC (O)?*

**Introduction**

Evidence from preclinical models of local anesthetic systemic toxicity (LAST) and clinical case reports of successful reversal of severe LAST and LAST-induced cardiac arrest in the perioperative setting suggest that adult patients with cardiac arrest due to suspected LAST may benefit from the use of intravenous lipid therapy. However, the evidence remains limited.

**Consensus on science**

For the important outcomes of ROSC, survival with favorable neurologic/functional outcome at discharge, and survival at discharge, low-quality evidence (downgraded for imprecision and risk of bias, respectively) from 13 case reports showed that lipid emulsion was effective in reversing PERIOPCA due to mepivacaine, ropivacaine, bupivacaine, lidocaine, with the vast majority of patients being discharged with a favorable neurologic outcome (100-112). One in-hospital death has been attributed to bronchopneumonia, while the reported side effects of lipid emulsion were hyperamylasemia without signs or symptoms of acute pancreatitis and lipemia interfering with blood samples analysis (Common Terminology Criteria for Adverse Events CTCAE Grade 1) (102,113,114).

**Treatment recommendations**

In adult patients with PERIOPCA due to confirmed or suspected LAST, it may be reasonable to use lipid therapy (COR/LOE: IIb/C-LD).

**Values, Preferences, and Task Force Insights**

Given the high mortality of LAST-induced cardiac arrest, the absence of an alternative antidote, and the absence of moderate-severe adverse effects, we recommend the use of lipid therapy in patients with PERIOPCA due to confirmed or suspected LAST. Further research is necessary for the full clarification of the effects of lipid therapy in this setting. However, the Task Force recognizes the ethical issues and hurdles for designing a clinical trial in this population.

**PICO 15**

*Among adults who are in cardiac arrest in the perioperative setting (P), does use of ultrasound (including echocardiography or other organ assessments) during CPR (I), compared with conventional CPR and resuscitation without use of ultrasound (C), change survival with favorable neurologic/functional outcome at discharge, 30 days, 60 days, 180 days, and/or 1 year; survival only at discharge, 30 days, 60 days, 180 days, and/or 1 year; ROSC (O)?*

**Introduction**

Point-of-care ultrasound may demonstrate real time physiologic data and reflect the dynamic changes in response to treatment. In the peri-arrest period, it may assist in the diagnosis of reversible causes of cardiac arrest and rhythm recognition, as well as in the assessment of post-cardiac arrest hypotension (9,57). Another advantage of the point-of-care ultrasound is the direct evaluation of chest compressions, which may guide the adjustment of hands location and improve CPR. This technology may offer additional data in the clinical assessment of patients suffering from cardiac arrest and may enhance individualized management.

**Consensus on science**

We did not identify any evidence to address the critical outcomes of survival with favorable neurologic/functional outcome at discharge, 30 days, 60 days, 180 days, and/or 1 year; survival only at discharge, 30 days, 60 days, 180 days, and/or 1 year; and ROSC.

**Treatment recommendations**

In patients with PERIOPCA, it may be reasonable to use point-of-care ultrasound to improve CPR and increase survival rates (COR/LOE: IIb/C-EO).

**Values, Preferences, and Task Force Insights**

In order to make this recommendation, the Task Force acknowledges the lack of studies in the perioperative setting. Point-of-care ultrasound can enhance the decision-making process during CPR and the latest ERC and AHA guidelines recommend performing an ultrasound when a reversible cause of cardiac arrest is suspected. However, it must not interfere with the standard cardiac arrest treatment protocol and can be detrimental if the pauses of CPR are too long. Considering the advantages of ultrasound, the Task Force considers reasonable to use it in patients with PERIOPCA. In this setting, however, its effectiveness remains unknown and further research is necessary.

**PICO 16**

*Among adults who are in cardiac arrest in the perioperative setting (P), does the use of ECPR techniques (including extracorporeal membrane oxygenation or cardiopulmonary bypass) (I), compared with manual CPR or mechanical CPR (C), change survival with favorable neurologic/functional outcome at discharge, 30 days, 60 days, 180 days, and/or 1 year; survival only at discharge, 30 days, 60 days, 180 days, and/or 1 year; ROSC (O)?*

**Introduction**

Extracorporeal cardiopulmonary resuscitation (ECPR) refers to the use of venoarterial extracorporeal membrane oxygenation when conventional CPR has failed to provide ROSC or non-sustained ROSC. The technique is technically challenging but can be applied in patients with witnessed arrest, initiation of good quality CPR within 5 min from the onset of the arrest, no ROSC within 15 min, and time to cannulation within 30-60 min. ECPR has many advantages as it may provide greater global blood flow than conventional CPR and can be considered as a “bridge” to appropriate treatment of the underlying cause of cardiac arrest.

**Consensus on science**

For the critical outcome of ROSC, survival to hospital discharge, survival to hospital discharge with good neurologic outcome, survival at 30 days, survival at 6 months, survival at one year, and survival at one year with good neurologic outcome only two observational studies were identified (115,116). These studies had very serious (ROSC) and serious risk of bias (all other outcomes) and the overall certainty of evidence was rated as very low for all outcomes.

**Treatment recommendations**

In adult patients with PERIOPCA, it may be reasonable to use ECPR as a rescue therapy when CPR has failed to provide ROSC or non-sustained ROSC (COR/LOE: IIb/C-LD).

**Values, Preferences, and Task Force Insights**

In making this recommendation, we acknowledge that patients with refractory cardiac arrest usually have an extremely high mortality rate. Although these patients are not responsive to standard resuscitation efforts, the perioperative setting facilitate the early onset of high-quality CPR. Therefore, ECPR has a potential benefit despite the overall poor quality of evidence and lack of randomized trials. Despite the fact that ECPR is a complex intervention, it may sustain perfusion while another intervention is performed and may be successful, especially in institutions with significant experience on the technique.

**PICO 17**

*Among adults with ROSC after cardiac arrest in the perioperative setting (P), does titration of therapy to achieve a specific hemodynamic goal (e.g., MAP greater than 65 mm Hg) (I), compared with no hemodynamic goal (C), change survival with favorable neurologic/functional outcome at discharge, 30 days, 60 days, 180 days, and/or 1 year; survival at discharge, 30 days, 60 days, 180 days, and/or 1 year (O)?*

**Introduction**

The postresuscitation period is characterized by a wide range of physiological derangements including hemodynamic instability. Patients may require advanced cardiac output monitoring and inotropes/vasopressors to optimize preload or afterload and achieve specific hemodynamic goals. Optimal management requires consideration of multiple processes simultaneously and specialized institutions capable of providing advanced care therapies. There is paucity of data to definitively recommend one agent or a choice of agents according to the goals of therapy, but there are no definitive data on the optimal target pressure goals. In these patients, adequate organ perfusion should be ensured and hemodynamic parameters should be monitored frequently.

**Consensus on science**

We did not identify any evidence to address the critical outcomes of survival with favorable neurologic/functional outcome at discharge, 30 days, 60 days, 180 days, and/or 1 year; survival at discharge, 30 days, 60 days, 180 days, and/or 1 year.

**Treatment recommendations**

In patients with ROSC after PERIOPCA, it may be reasonable to target the hemodynamics goals to optimize tissue perfusion as indicated by an adequate urine output (1 ml kg^−1^ h^−1^) and normal or decreasing plasma lactate values, taking into consideration the patient’s normal blood pressure, the cause of the arrest and the severity of myocardial dysfunction (COR/LOE: IIb/C-EO).

**Values, Preferences, and Task Force Insights**

In making this recommendation, we acknowledge the lack of evidence in the perioperative setting. Post-cardiac arrest syndrome heavily influences hemodynamics in resuscitated patients and although optimal hemodynamic goals remain undefined, initiatives to maintain adequate tissue perfusion and oxygen delivery must be instituted. Considering that multiple organ systems may be affected secondary to post-cardiac arrest syndrome and taking into account the technological advantages in the perioperative setting, an aggressive management should be pursued following ROSC.

**PICO 18**

*Among adults with ROSC after cardiac arrest in the perioperative setting (P), do prophylactic antiarrhythmic drugs given immediately after ROSC (I), compared with not giving antiarrhythmic drugs (C), change survival with favorable neurologic/functional outcome at discharge, 30 days, 60days, 180 days, and/or 1 year; development of cardiac arrest; survival only at discharge, 30 days, 60 days, 180 days, and/or 1 year; recurrence of ventricular fibrillation/pulseless ventricular tachycardia; incidence of arrhythmias (O)?*

**Introduction**

Cardiac arrest with a shockable rhythm may require the administration of antiarrhythmics. Amiodarone and lidocaine are used in refractory ventricular fibrillation/pulseless ventricular tachycardia to increase the success of subsequent defibrillation attempts and prevent recurrences (117-119). In 2018, the ERC update recommended that amiodarone or lidocaine should be given after three defibrillation attempts irrespective of whether they are consecutive shocks or interrupted by CPR, or for recurrent ventricular fibrillation/pulseless ventricular tachycardia during cardiac arrest (120). However, a weak recommendation based on low certainty evidence applies to both amiodarone or lidocaine and the effects of these drugs on survival to hospital discharge or neurologic outcome remain uncertain (121).

**Consensus on science**

We did not identify any evidence to address the critical outcomes of survival with favorable neurologic/functional outcome at discharge, 30 days, 60days, 180 days, and/or 1 year; development of cardiac arrest; survival only at discharge, 30 days, 60 days, 180 days, and/or 1 year; recurrence of ventricular fibrillation/pulseless ventricular tachycardia; incidence of arrhythmias.

**Treatment recommendations**

In the perioperative setting, it may be reasonable to administer antiarrhythmics immediately after ROSC to treat postresuscitation arrhythmias, especially in refractory cases, and prevent recurrences (COR/LOE: IIb/C-EO).

**Values, Preferences, and Task Force Insights**

Although cardiac arrhythmias are a significant cause of morbidity and mortality in the perioperative period, clinical trials on the use of antiarrhythmics in this setting are scarce. Amiodarone has very little negative inotropic activity and a low rate of ventricular pre-arrhythmia (122). However, it has been associated with a relatively high incidence of side effects, which complicates its use. Although amiodarone and lidocaine may aggravate postresuscitation hypotension, the risks from their administration may be low in the perioperative setting (123). It remains unclear whether antiarrhythmic administration offer any advantage to patients with ROSC after PERIOPCA.

**PICO 19**

*Among successfully resuscitated perioperative cardiac arrest patients who receive mechanical ventilation (P), does permissive hypercapnia in the context of a lung-protective ventilatory strategy with low tidal volumes (i.e., 6 mL/kg predicted body weight) and PEEP/FiO_2_ set according to the ARDSnet protocol (I), compared with normocapnia in the context of a ventilatory strategy employing moderate tidal volumes (i.e., 7.5-10 mL/kg predicted body weight and PEEP levels of 5-10 cmH_2_O (C), change survival with favorable neurologic/functional outcome at discharge, 30 days, 60 days, 180 days, and/or 1 year; survival only at discharge, 30 days, 60 days, 180 days, and/or 1 year (O)?*

**Introduction**

Although mechanical ventilation is a mainstay of therapy in the perioperative setting, it can be a direct cause of injury and may affect morbidity and mortality. Lung-protective ventilation may improve outcome in patients with acute respiratory distress syndrome (ARDS), i.e., by reducing tidal volume (V_t_) (6 ml/kg vs. 12 ml/kg of predicted body weight, PBV) and limiting plateau pressure (P_pl_) to 28-30 cmH_2_O (124). In addition, minimization of driving pressure is a key component in reducing ventilator-induced lung injury (VILI) (125). Several parameters may impair lung function after ROSC (29,30), while VILI *per se* may affect distant organs through the secretion of proinflammatory cytokines, aggravating the postcardiac arrest syndrome and decreasing survival rates (126). Therefore, lung-protective ventilation may be beneficial in patients with PERIOPCA.

**Consensus on science**

We did not identify any evidence to address the critical outcomes of survival with favorable neurologic/functional outcome at discharge, 30 days, 60 days, 180 days, and/or 1 year; survival only at discharge, 30 days, 60 days, 180 days, and/or 1 year (O).

**Treatment recommendations**

In patients with ROSC after PERIOPCA, a lung-protective ventilation strategy (reducing tidal volume, plateau pressure, and driving pressure) and mild hypercapnia (PaCO_2_ of 40-50 mmHg) might be reasonable for improving outcome (COR/LOE: IIb/C-EO).

**Values, Preferences, and Task Force Insights**

The Task Force acknowledges that mechanical ventilation during the post-cardiac arrest period can cause biotrauma (7,8), which can be further aggravated by the systemic inflammatory response (7). Several other factors may cause lung injury, such as the high fraction of inspired oxygen (FiO_2_) during and after resuscitation, lung contusions due to chest compressions, aspiration, impairment of normal respiratory muscle tone, or secondary infections.

Lung-protective mechanical ventilation may significantly reduce mortality in patients with lung injury (124). The addition of a positive end-expiratory pressure (PEEP) is usually part of this strategy in order to prevent de-recruitment and intratidal opening and closing of the alveolar units (127). On the other hand, the National Heart, Lung, and Blood Institute recommendations for PEEP and FiO_2_ do not take into consideration the heterogeneity of critically ill patients (124). A more physiology-driven approach for tailoring PEEP should aim at maximizing respiratory-system compliance (C_RS_) and minimizing driving pressure (V_t_/C_RS_ or P_pl_ - PEEP) (125).

In lung-protective ventilation, permissive hypercapnia is a strategy to minimize VILI (10). Hypercapnia is associated with anti-inflammatory effects, increases in pulmonary vascular resistance and right ventricular afterload, cerebral vasodilation, and negative inotropism with reflex increase in heart rate (10). A recent meta-analysis of heterogenous observational studies confirmed the detrimental effect of hypocapnia and hypercapnia in comparison to normocapnia, but the evidence remains limited to exclude the safety of “mild” hypercapnia (128). Mild hypercapnia (PaCO_2_ 50-55 mmHg) was shown to be beneficial in terms of cerebral oxygen saturation and does not seem to worsen survival or neurologic function at 6 months after cardiac arrest (129,130). In the COMACARE study, targeting low-normal (33-35 mmHg) or high-normal (43-45 mmHg) PaCO_2_ did not affect the serum concentration of neuron-specific enolase at 48 h, while high-normal PaCO_2_ resulted in better cerebral oxygen saturation which may indicate higher cerebral blood flow and oxygen delivery (131). However, higher PaCO_2_ levels may worsen cerebral edema, lead to respiratory acidosis, and impair right ventricular function, which may all contribute to poor outcomes (131-133). In consideration of the clear benefits of limiting lung injury after ROSC, a lung-protective ventilation strategy and mild hypercapnia (PaCO_2_ of 40-50 mmHg) might be reasonable for improving outcome after PERIOPCA.

**PICO 20**

*Among adults with ROSC after cardiac arrest in the perioperative setting (P), does ventilation to a PaO_2_ goal of <200 mmHg (I), compared to PaO_2_ goal of >200 mmHg (C), change survival at discharge, 30days, 60 days, 180 days, and/or 1 year; survival with favorable neurologic/functional outcome at discharge, 30 days, 60 days, 180 days, and/or 1 year (O)?*

**Introduction**

Maintenance of systemic and cerebral oxygenation is paramount for improving survival rates with good functional outcome after cardiac arrest. The brain has an extremely high metabolic rate and its oxygen demands are high, especially after a global ischemic event. In patients with postresuscitation syndrome, hemodynamic instability may also compromise cerebral perfusion and oxygenation, which may be detrimental. The increased oxygen consumption in the central nervous system may lead to ventilation with increased FiO_2_ after PERIOPCA. However, hyperoxemia may aggravate brain injury and neurological outcome (134,135) although controversial data exist (136,137).

**Consensus on science**

We did not identify any evidence to address the critical outcomes of change survival at discharge, 30days, 60 days, 180 days, and/or 1 year; survival with favorable neurologic/functional outcome at discharge, 30 days, 60 days, 180 days, and/or 1 year.

**Treatment recommendations**

In patients with PERIOPCA, it may be reasonable to maintain normoxemia and avoid hyperoxemia (PaO_2_ goal of <200 mmHg) in order to improve short and long-term outcome (COR/LOE: IIb/C-EO).

**Values, Preferences, and Task Force Insights**

In making this recommendation, we place a high value in the devastating consequences of hypoxia or hyperoxia during the peri-arrest period. Exposure to supranormal partial pressures of arterial oxygen after ROSC was previously demonstrated to be detrimental to neurological outcomes (138,139). The main mechanism involves reperfusion injury and the formation of reactive oxygen species, resulting in oxidative impairment of mitochondrial respiration. Preclinical and clinical studies support the hypothesis that hyperoxia after ROSC worsens brain damage and decrease survival (140-146). However, a randomized clinical trial reported no decrease in mortality at 1 year with the use of supplemental oxygen compared with room air in patients with acute myocardial infarction (147). In consideration of the aforementioned evidence, normoxemia should be maintained in patients with PERIOPCA in an effort to improve short and long-term outcome.

**PICO 21**

*In patients with ROSC after cardiac arrest in the perioperative setting (P), does induction of TTM (target temperature 32-36◦C) for any duration or before some time point (e.g., 1 h after ROSC (I), compared with normothermia (C), change survival with favorable neurologic/functional outcome at discharge, 30 days, 60 days, 180 days, and/or 1 year; survival only at discharge, 30 days, 60 days, 180 days, and/or 1 year (O)?*

**Introduction**

Targeted Temperature Management (TTM) has been shown to improve outcome in comatose adult patients successfully resuscitated from cardiac arrest from various causes (148-151). Consequently, guidelines constantly confirm the importance of controlling body temperature after cardiac arrest between 32-36°C (152). However, given the potential hypothermia-induced complications (especially coagulopathy in surgical patients), applicability to the particular setting of PERIOPCA requires rigorous evaluation (153,154).

**Consensus on science**

For the important outcome of 1-year favorable functional outcome (CPC 1-2), moderate quality evidence (downgraded for limitations) showed that TTM use was not independently associated with 1-year favorable outcome (OR 0.82; 95% CI 0.27-2.46, p=0.72) (155).

**Treatment recommendations**

In comatose patients with PERIOPCA, it may be reasonable to maintain normothermia in order to improve short and long-term outcome (COR/LOE: IIb/C-EO). Potential neurological benefit should be balanced against the hemorrhagic risk related to hypothermia (<37°C) in this surgical setting.

**Values, Preferences, and Task Force Insights**

Evaluation of the use of TTM in the perioperative setting suffers from the paucity of available data. Future studies focusing on PERIOPCA should mention the proportion of patients receiving TTM.

**PICO 22**

*Among adults with ROSC who are treated or not with TTM in the perioperative setting (P), does any clinical variable when abnormal (e.g., clinical exam, EEG, somatosensory evoked potentials [SSEPs], imaging, other) (I), compared with any clinical variable when normal (C), reliably predict death or poor neurologic outcome at discharge, 30 days, 60 days, 180 days, and/or 1year; death only at discharge, 30 days, 60 days, 180 days, and/or 1 year (O)?*

**Introduction**

Hypoxic-ischemic brain injury is the main cause of death in patients who remain comatose after resuscitation from cardiac arrest. In these patients, brain dysfunction is the main determinant of prognosis. Time to regaining consciousness varies widely, but today there are several methods for assessing the severity of brain injury. Current guidelines for both out-of-hospital and in-hospital cardiac arrest recommend performing prognostication no earlier than 72 h after ROSC (156). However, even the most robust neurological predictors are not 100% specific while many confounders may exist, such as sedation, hypothermia, and organ injury, especially in patients undergoing high-risk or emergency surgery.

**Consensus on science**

We did not identify any evidence to address the critical outcomes of prediction of death or poor neurologic outcome at discharge, 30 days, 60 days, 180 days, and/or 1year; death only at discharge, 30 days, 60 days, 180 days, and/or 1 year.

**Treatment recommendations**

In patients with PERIOPCA and ROSC, it may be reasonable to use a multimodal strategy for prognostication, giving emphasis on allowing sufficient time for neurological recovery and to enable sedatives/paralytics to be cleared (COR/LOE: IIb/C-EO).

**Values, Preferences, and Task Force Insights**

In order to make this recommendation, the Task Force was keen to acknowledge the lack of data in the surgical population. Although standardized optimal neuroprognostication following cardiac arrest is desirable, the heterogenous population of surgical patients and the several methods for assessing neurological function make the conduction of controlled studies on this subject difficult. Therefore, a multimodal approach combining multiple prognostication tests is recommended. Future studies should focus on objective, reproducible findings on physical examination in conjunction with laboratory and imaging testing.

**REFERENCES**

1. Pantazopoulos C, Xanthos T, Pantazopoulos I, Papalois A, Kouskouni E, Iacovidou N. A Review of Carbon Dioxide Monitoring During Adult Cardiopulmonary Resuscitation. Heart Lung Circ 2015;24:1053-61.
2. Heradstveit BE, Sunde K, Sunde GA, Wentzel-Larsen T, Heltne JK. Factors complicating interpretation of capnography during advanced life support in cardiac arrest--a clinical retrospective study in 575 patients. Resuscitation 2012;83:813-18.
3. Gazmuri RJ, von Planta M, Weil MH, Rackow EC. Arterial PCO2 as an indicator of systemic perfusion during cardiopulmonary resuscitation. Crit Care Med 1989;17:237-40.
4. Pokorna M, Necas E, Kratochvil J, Skripsky R, Andrlik M, Franek O. A sudden increase in partial pressure end-tidal carbon dioxide (P(ET)CO(2)) at the moment of return of spontaneous circulation. J Emerg Med 2010;38:614-21.
5. Sehra R, Underwood K, Checchia P. End tidal CO2 is a quantitative measure of cardiac arrest. Pacing Clin Electrophysiol 2003;26:515-7.
6. Jin X, Weil MH, Tang W, et al. End-tidal carbon dioxide as a noninvasive indicator of cardiac index during circulatory shock. Crit Care Med 2000;28:2415-9.
7. Neumar RW, Nolan JP, Adrie C, et al. Post-cardiac arrest syndrome: epidemiology, pathophysiology, treatment, and prognostication. A consensus statement from the International Liaison Committee on Resuscitation (American Heart Association, Australian and New Zealand Council on Resuscitation, European Resuscitation Council, Heart and Stroke Foundation of Canada, InterAmerican Heart Foundation, Resuscitation Council of Asia, and the Resuscitation Council of Southern Africa); the American Heart Association Emergency Cardiovascular Care Committee; the Council on Cardiovascular Surgery and Anesthesia; the Council on Cardiopulmonary, Perioperative, and Critical Care; the Council on Clinical Cardiology; and the Stroke Council. Circulation 2008;118:2452-83.
8. Johnson NJ, Carlbom DJ, Gaieski DF. Ventilator Management and Respiratory Care After Cardiac Arrest: Oxygenation, Ventilation, Infection, and Injury. Chest 2018;153:1466-77.
9. Link MS, Berkow LC, Kudenchuk PJ, et al. Part 7: Adult Advanced Cardiovascular Life Support: 2015 American Heart Association Guidelines Update for Cardiopulmonary Resuscitation and Emergency Cardiovascular Care. Circulation 2015;132:S444-464. Erratum in: Circulation 2015;132:e385.
10. Barnes T, Zochios V, Parhar K. Re-examining Permissive Hypercapnia in ARDS: A Narrative Review. Chest 2018;154:185-95.
11. Chalkias A, Arnaoutoglou E, Xanthos T. Personalized physiology-guided resuscitation in highly monitored patients with cardiac arrest-the PERSEUS resuscitation protocol. Heart Fail Rev 2019;24:473-80.
12. Sutton RM, Friess SH, Maltese MR, et al. Hemodynamic-directed cardiopulmonary resuscitation during in-hospital cardiac arrest. Resuscitation 2014;85:983-6.
13. Sutton RM, French B, Meaney PA, et al; American Heart Association's Get With The Guidelines–Resuscitation Investigators. Physiologic monitoring of CPR quality during adult cardiac arrest: A propensity-matched cohort study. Resuscitation 2016;106:76-82.
14. Maillard J, Sologashvili T, Diaper J, Licker MJ, Keli Barcelos G. A Case of Persistence of Normal Tissue Oxygenation Monitored by Near-Infrared Spectroscopy (NIRS) Values Despite Prolonged Perioperative Cardiac Arrest. Am J Case Rep 2019;20:21-5.
15. Paarmann H, Heringlake M, Sier H, Schön J. The association of non-invasive cerebral and mixed venous oxygen saturation during cardiopulmonary resuscitation. Interact Cardiovasc Thorac Surg 2010;11:371-3.
16. Yunoki K, Sasaki R, Taguchi A, Maekawa S, Ueta H, Yamazaki K. Successful recovery without any neurological complication after intraoperative cardiopulmonary resuscitation for an extended period of time in the lateral position: a case report. JA Clin Rep 2016;2:7.
17. Weisfeldt ML, Becker LB. Resuscitation after cardiac arrest: a 3-phase time-sensitive model. JAMA 2002;288:3035-8.
18. Peberdy MA, Kaye W, Ornato JP, et al. Cardiopulmonary resuscitation of adults in the hospital: a report of 14720 cardiac arrests from the National Registry of Cardiopulmonary Resuscitation. Resuscitation 2003;58:297-308.
19. Chan PS, Krumholz HM, Nichol G, Nallamothu BK; American Heart Association National Registry of Cardiopulmonary Resuscitation Investigators. Delayed time to defibrillation after in-hospital cardiac arrest. N Engl J Med 2008;358:9-17.
20. Skogvoll E, Nordseth T. The early minutes of in-hospital cardiac arrest: shock or CPR? A population based prospective study. Scand J Trauma Resusc Emerg Med 2008;16:11.
21. Davis D, Aguilar SA, Sell R, Minokadeh A, Husa R. A focused investigation of expedited, stack of three shocks versus chest compressions first followed by single shocks for monitored ventricular fibrillation/ventricular tachycardia cardiopulmonary arrest in an in-hospital setting. J Hosp Med 2016;11:264-8.
22. Chan PS, Krumholz HM, Spertus JA, et al; American Heart Association National Registry of Cardiopulmonary Resuscitation (NRCPR) Investigators. Automated external defibrillators and survival after in-hospital cardiac arrest. JAMA 2010;304:2129-36.
23. Monsieurs KG, Nolan JP, Bossaert LL, et al; ERC Guidelines 2015 Writing Group. European Resuscitation Council Guidelines for Resuscitation 2015: Section 1. Executive summary. Resuscitation 2015;95:1-80.
24. Angelos MG, Butke RL, Panchal AR, et al. Cardiovascular response to epinephrine varies with increasing duration of cardiac arrest. Resuscitation 2008;77:101-10.
25. Neumar RW, Brown CG, Van Ligten P, Hoekstra J, Altschuld RA, Baker P. Estimation of myocardial ischemic injury during ventricular fibrillation with total circulatory arrest using high-energy phosphates and lactate as metabolic markers. Ann Emerg Med 1991;20:222-9.
26. Bircher NG, Chan PS, Xu Y, American Heart Association's Get With The Guidelines-Resuscitation I. Delays in Cardiopulmonary Resuscitation, Defibrillation, and Epinephrine Administration All Decrease Survival in In-hospital Cardiac Arrest. Anesthesiology 2019;130:414-22.
27. Andersen LW, Kurth T, Chase M, et al; American Heart Association’s Get With The Guidelines-Resuscitation Investigators. Early administration of epinephrine (adrenaline) in patients with cardiac arrest with initial shockable rhythm in hospital: propensity score matched analysis. BMJ 2016;353:i1577.
28. Chalkias A, Xanthos T. Post-cardiac arrest syndrome: Mechanisms and evaluation of adrenal insufficiency. World J Crit Care Med 2012;1:4-9.
29. Chalkias A, Xanthos T. Pathophysiology and pathogenesis of post-resuscitation myocardial stunning. Heart Fail Rev 2012;17:117-28.
30. Attaran RR, Ewy GA. Epinephrine in resuscitation: curse or cure? Future Cardiol 2010;6:473-82.
31. Brown CG, Martin DR, Pepe PE, et al. A comparison of standard-dose and high-dose epinephrine in cardiac arrest outside the hospital. The Multicenter High-Dose Epinephrine Study Group. N Engl J Med 1992;327:1051-5.
32. Woodhouse SP, Cox S, Boyd P, Case C, Weber M. High dose and standard dose adrenaline do not alter survival, compared with placebo, in cardiac arrest. Resuscitation 1995;30:243-49.
33. Gueugniaud PY, Mols P, Goldstein P, et al. A comparison of repeated high doses and repeated standard doses of epinephrine for cardiac arrest outside the hospital. European Epinephrine Study Group. N Engl J Med 1998;339:1595-1601.
34. Perondi MB, Reis AG, Paiva EF, Nadkarni VM, Berg RA. A comparison of high-dose and standard-dose epinephrine in children with cardiac arrest. N Engl J Med 2004;350:1722-30.
35. Xanthos T, Pantazopoulos I, Demestiha T, Stroumpoulis K. Epinephrine in ventricular fibrillation: friend or foe? A review for the Emergency Nurse. J Emerg Nurs 2011;37:408-12; quiz 425-6.
36. Niemann JT, Cairns CB, Sharma J, Lewis RJ. Treatment of prolonged ventricular fibrillation. Immediate countershock versus high-dose epinephrine and CPR preceding countershock. Circulation 1992;85:281-7.
37. Herlitz J, Ekstrom L, Wennerblom B, Axelsson A, Bang A, Holmberg S. Adrenaline in out-of-hospital ventricular fibrillation. Does it make any difference? Resuscitation 1995;29:195-201.
38. Han F, Wang Y, Wang Y, et al. Intraoperative cardiac arrest: A 10-year study of patients undergoing tumorous surgery in a tertiary referral cancer center in China. Medicine (Baltimore) 2017;96:e6794.
39. Zhong JQ, Dorian P. Epinephrine and vasopressin during cardiopulmonary resuscitation. Resuscitation 2005;66:263-9.
40. Cooper S, Janghorbani M, Cooper G. A decade of in-hospital resuscitation: outcomes and prediction of survival? Resuscitation 2006;68:231-7.
41. Behringer W, Kittler H, Sterz F, et al. Cumulative epinephrine dose during cardiopulmonary resuscitation and neurologic outcome. Ann Intern Med 1998;129:450-6.
42. Wayne MA, Racht EM, Aghababian RV, Kudenchuk PJ, Ornato JP, Slovis CM. Prehospital management of cardiac arrest: how useful are vasopressor and antiarrhythmic drugs? Prehosp Emerg Care 2002;6:72-80.
43. Panchal AR, Berg KM, Kudenchuk PJ, et al. 2018 American Heart Association Focused Update on Advanced Cardiovascular Life Support Use of Antiarrhythmic Drugs During and Immediately After Cardiac Arrest: An Update to the American Heart Association Guidelines for Cardiopulmonary Resuscitation and Emergency Cardiovascular Care. Circulation 2018;138:e740-9.
44. Kudenchuk PJ, Cobb LA, Copass MK, et al. Amiodarone for resuscitation after out-of-hospital cardiac arrest due to ventricular fibrillation. N Engl J Med 1999;341:871-8.
45. Pollak PT, Wee V, Al-Hazmi A, Martin J, Zarnke KB. The use of amiodarone for in-hospital cardiac arrest at two tertiary care centres. Can J Cardiol 2006;22:199-202.
46. Rea RS, Kane-Gill SL, Rudis MI, et al. Comparing intravenous amiodarone or lidocaine, or both, outcomes for inpatients with pulseless ventricular arrhythmias. Crit Care Med 2006;34:1617-23.
47. Thel MC, Armstrong AL, McNulty SE, Califf RM, O'Connor CM. Randomised trial of magnesium in in-hospital cardiac arrest. Duke Internal Medicine Housestaff. Lancet 1997;350:1272-6.
48. Shiga T, Tanaka K, Kato R, et al; Refractory VT/VF, Prospective Evaluation to Differentiate Lidocaine Efficacy from Nifekalant (RELIEF) Study Investigators. Nifekalant versus lidocaine for in-hospital shock-resistant ventricular fibrillation or tachycardia. Resuscitation 2010;81:47-52.
49. Dorian P, Cass D, Schwartz B, Cooper R, Gelaznikas R, Barr A. Amiodarone as compared with lidocaine for shock-resistant ventricular fibrillation. N Engl J Med 2002;346:884-90.
50. Kudenchuk PJ, Brown SP, Daya M, et al; Resuscitation Outcomes Consortium Investigators. Amiodarone, Lidocaine, or Placebo in Out-of-Hospital Cardiac Arrest. N Engl J Med 2016;374:1711-22.
51. Cushing DJ, Kowey PR, Cooper WD, Massey BW, Gralinski MR, Lipicky RJ. PM101: a cyclodextrin-based intravenous formulation of amiodarone devoid of adverse hemodynamic effects. Eur J Pharmacol 2009;607:167-72.
52. Sharma A, Arora L, Subramani S, Simmons J, Mohananey D, Ramakrishna H. Analysis of the 2018 American Heart Association Focused Update on Advanced Cardiovascular Life Support Use of Antiarrhythmic Drugs During and Immediately After Cardiac Arrest. J Cardiothorac Vasc Anesth 2020;34:537-44.
53. Souney PF, Cooper WD, Cushing DJ. PM101: intravenous amiodarone formulation changes can improve medication safety. Expert Opin Drug Saf 2010;9:319-33.
54. Aufderheide TP, Sigurdsson G, Pirrallo RG, et al. Hyperventilation-induced hypotension during cardiopulmonary resuscitation. Circulation 2004;109:1960-5.
55. Chalkias A, Xanthos T. Timing positive-pressure ventilation during chest compression: the key to improving the thoracic pump? Eur Heart J Acute Cardiovasc Care 2015;4:24-7.
56. Chalkias A, Pavlopoulos F, Koutsovasilis A, d'Aloja E, Xanthos T. Airway pressure and outcome of out-of-hospital cardiac arrest: A prospective observational study. Resuscitation 2017;110:101-6.
57. Soar J, Nolan JP, Böttiger BW, et al; Adult advanced life support section Collaborators. European Resuscitation Council Guidelines for Resuscitation 2015: Section 3. Adult advanced life support. Resuscitation 2015;95:100-47.
58. Abella BS, Alvarado JP, Myklebust H, et al. Quality of cardiopulmonary resuscitation during in-hospital cardiac arrest. JAMA 2005;293:305-10.
59. Abella BS, Edelson DP, Kim S, et al. CPR quality improvement during in-hospital cardiac arrest using a real-time audiovisual feedback system. Resuscitation 2007;73:54-61.
60. Edelson DP, Litzinger B, Arora V, et al. Improving in-hospital cardiac arrest process and outcomes with performance debriefing. Arch Intern Med 2008;168:1063-9.
61. Yeung J, Chilwan M, Field R, Davies R, Gao F, Perkins GD. The impact of airway management on quality of cardiopulmonary resuscitation: an observational study in patients during cardiac arrest. Resuscitation 2014;85:898-904.
62. Edelson DP, Eilevstjonn J, Weidman EK, Retzer E, Hoek TL, Abella BS. Capnography and chest-wall impedance algorithms for ventilation detection during cardiopulmonary resuscitation. Resuscitation 2010;81:317-22.
63. Huang J, Lu Y and Chen J. Perioperative Pulmonary Embolism Prevention and Treatment. Austin Spine 2017;1:1001.
64. Desciak MC, Martin DE. Perioperative pulmonary embolism: diagnosis and anesthetic management. J Clin Anesth 2011;23:153-65.
65. Li X, Fu QL, Jing XL, et al. A meta-analysis of cardiopulmonary resuscitation with and without the administration of thrombolytic agents. Resuscitation 2006;70:31-6.
66. Böttiger BW, Arntz HR, Chamberlain DA, et al; TROICA Trial Investigators; European Resuscitation Council Study Group. Thrombolysis during resuscitation for out-of-hospital cardiac arrest. N Engl J Med 2008;359:2651-62.
67. Janata K, Holzer M, Kürkciyan I, et al. Major bleeding complications in cardiopulmonary resuscitation: the place of thrombolytic therapy in cardiac arrest due to massive pulmonary embolism. Resuscitation 2003;57:49-55.
68. Kürkciyan I, Meron G, Sterz F, et al. Pulmonary embolism as a cause of cardiac arrest: presentation and outcome. Arch Intern Med 2000;160:1529-35.
69. Javaudin F, Lascarrou JB, Le Bastard Q, et al. Thrombolysis During Resuscitation for Out-of-Hospital Cardiac Arrest Caused by Pulmonary Embolism Increases 30-Day Survival: Findings From the French National Cardiac Arrest Registry. Chest 2019;156:1167-75.
70. Jeejeebhoy FM, Zelop CM, Lipman S, et al; American Heart Association Emergency Cardiovascular Care Committee, Council on Cardiopulmonary, Critical Care, Perioperative and Resuscitation, Council on Cardiovascular Diseases in the Young, and Council on Clinical Cardiology. Cardiac Arrest in Pregnancy: A Scientific Statement From the American Heart Association. Circulation 2015;132:1747-73.
71. Mhyre JM, Tsen LC, Einav S, Kuklina EV, Leffert LR, Bateman BT. Cardiac arrest during hospitalization for delivery in the United States, 1998-2011. Anesthesiology 2014;120:810-8.
72. Sultan AA, Tata LJ, West J, et al. Risk factors for first venous thromboembolism around pregnancy: a population-based cohort study from the United Kingdom. Blood 2013;121:3953-61.
73. Jeejeebhoy FM, Zelop CM, Windrim R, Carvalho JC, Dorian P, Morrison LJ. Management of cardiac arrest in pregnancy: a systematic review. Resuscitation 2011;82:801-9.
74. Martillotti G, Boehlen F, Robert-Ebadi H, Jastrow N, Righini M, Blondon M. Treatment options for severe pulmonary embolism during pregnancy and the postpartum period: a systematic review. J Thromb Haemost 2017;15:1942-50.
75. Agerstrand C, Abrams D, Biscotti M, et al. Extracorporeal Membrane Oxygenation for Cardiopulmonary Failure During Pregnancy and Postpartum. Ann Thorac Surg 2016;102:774-9.
76. Webster CM, Smith KA, Manuck TA. Extracorporeal membrane oxygenation in pregnant and postpartum women: a ten-year case series. Am J Obstet Gynecol MFM 2020;2:100108.
77. Ong J, Zhang JJY, Lorusso R, MacLaren G, Ramanathan K. Extracorporeal membrane oxygenation in pregnancy and the postpartum period: a systematic review of case reports. Int J Obstet Anesth 2020;43:106-13.
78. Zhang JJY, Ong JA, Syn NL, et al. Extracorporeal Membrane Oxygenation in Pregnant and Postpartum Women: A Systematic Review and Meta-Regression Analysis. J Intensive Care Med 2021;36:220-8.
79. Boom M, Niesters M, Sarton E, Aarts L, Smith TW, Dahan A. Non-analgesic effects of opioids: opioid-induced respiratory depression. Curr Pharm Des 2012;18:5994-6004.
80. Varvarousis D, Varvarousi G, Iacovidou N, D'Aloja E, Gulati A, Xanthos T. The pathophysiologies of asphyxial vs dysrhythmic cardiac arrest: implications for resuscitation and post-event management. Am J Emerg Med 2015;33:1297-304.
81. Rzasa Lynn R, Galinkin JL. Naloxone dosage for opioid reversal: current evidence and clinical implications. Ther Adv Drug Saf 2018;9:63-88.
82. Chen MH, Liu TW, Xie L, Song FQ, He T. Does naloxone alone increase resuscitation rate during cardiopulmonary resuscitation in a rat asphyxia model? Am J Emerg Med 2006;24:567-72.
83. Wang Y, Gao L, Meng L. Small-dose naloxone combined with epinephrine improves the resuscitation of cardiopulmonary arrest. Am J Emerg Med 2008;26:898-901.
84. Shweta A, Malpas SC, Anderson WP, Evans RG. Effects of naloxone on the haemodynamic and renal functional responses to plasma volume expansion in conscious rabbits. Pflugers Arch 1999;439:150-7.
85. Gervais HW, Eberle B, Hennes HJ, et al. High dose naloxone does not improve cerebral or myocardial blood flow during cardiopulmonary resuscitation in pigs. Resuscitation 1997;34:255-61.
86. Foley PJ, Tacker WA, Voorhees WD, Ralston SH, Elchisak MA. Effects of naloxone on the adrenomedullary response during and after cardiopulmonary resuscitation in dogs. Am J Emerg Med 1987;5:357-61.
87. Saybolt MD, Alter SM, Dos Santos F, et al. Naloxone in cardiac arrest with suspected opioid overdoses. Resuscitation 2010;81:42-6.
88. Chou R, Korthuis PT, McCarty D, et al. Management of Suspected Opioid Overdose With Naloxone in Out-of-Hospital Settings: A Systematic Review. Ann Intern Med 2017;167:867-75.
89. Ito T, Saitoh D, Takasu A, Kiyozumi T, Sakamoto T, Okada Y. Serum cortisol as a predictive marker of the outcome in patients resuscitated after cardiopulmonary arrest. Resuscitation 2004;62:55-60.
90. Schultz CH, Rivers EP, Feldkamp CS, et al. A characterization of hypothalamic-pituitary-adrenal axis function during and after human cardiac arrest. Crit Care Med 1993;21:1339-47.
91. Tavakoli N, Bidari A, Shams Vahdati S. Serum Cortisol Levels as a Predictor of Neurologic Survival inSuccessfully Resuscitated Victims of Cardiopulmonary Arrest. J Cardiovasc Thorac Res 2012;4:107-11.
92. Schwitzer KW. Dexamethasone therapy in bradyasystolic prehospital cardiac arrest, abstract. Ann Emerg Med 1983;12:252.
93. White BC, Petinga TJ, Hoehner PJ, Wilson RF. Incidence, etiology, and outcome of pulseless idioventricular rhythm treated with dexamethasone during advanced CPR. JACEP 1979;8:188-93.
94. Jastremski M, Sutton-Tyrrell K, Vaagenes P, Abramson N, Heiselman D, Safar P. Glucocorticoid treatment does not improve neurological recovery following cardiac arrest. Brain Resuscitation Clinical Trial I Study Group. JAMA 1989;262:3427-30.
95. Tsai MS, Huang CH, Chang WT, et al. The effect of hydrocortisone on the outcome of out-of-hospital cardiac arrest patients: a pilot study. Am J Emerg Med 2007;25:318-25.
96. Mentzelopoulos SD, Zakynthinos SG, Tzoufi M, et al. Vasopressin, epinephrine, and corticosteroids for in-hospital cardiac arrest. Arch Intern Med 2009;169:15-24.
97. Mentzelopoulos SD, Malachias S, Chamos C, et al. Vasopressin, steroids, and epinephrine and neurologically favorable survival after in-hospital cardiac arrest: a randomized clinical trial. JAMA 2013;310:270-9.
98. Dixon WG, Abrahamowicz M, Beauchamp ME, et al. Immediate and delayed impact of oral glucocorticoid therapy on risk of serious infection in older patients with rheumatoid arthritis: a nested case-control analysis. Ann Rheum Dis 2012;71:1128-33.
99. Tseng CL, Chen YT, Huang CJ, et al. Short-term use of glucocorticoids and risk of peptic ulcer bleeding: a nationwide population-based case-crossover study. Aliment Pharmacol Ther 2015;42:599-606.
100. Hasan B, Asif T, Hasan M. Lidocaine-Induced Systemic Toxicity: A Case Report and Review of Literature. Cureus 2017;9:e1275.
101. Litz RJ, Popp M, Stehr SN, Koch T. Successful resuscitation of a patient with ropivacaine-induced asystole after axillary plexus block using lipid infusion. Anaesthesia 2006;61:800-1.
102. Marwick PC, Levin AI, Coetzee AR. Recurrence of cardiotoxicity after lipid rescue from bupivacaine-induced cardiac arrest. Anesth Analg 2009;108:1344-6.
103. Mazoit JX. Arrêt cardiaque et anesthésiques locaux [Cardiac arrest and local anaesthetics]. Presse Med 2013;42:280-6.
104. Rosenblatt MA, Abel M, Fischer GW, Itzkovich CJ, Eisenkraft JB. Successful use of a 20% lipid emulsion to resuscitate a patient after a presumed bupivacaine-related cardiac arrest. Anesthesiology 2006;105:217-8.
105. Scherrer V, Compere V, Loisel C, Dureuil B. Cardiac arrest from local anesthetic toxicity after a field block and transversus abdominis plane block: a consequence of miscommunication between the anesthesiologist and surgeon. A A Case Rep 2013;1:75-6.
106. Smith HM, Jacob AK, Segura LG, Dilger JA, Torsher LC. Simulation education in anesthesia training: a case report of successful resuscitation of bupivacaine-induced cardiac arrest linked to recent simulation training. Anesth Analg 2008;106:1581-4, table of contents.
107. Sonsino DH, Fischler M. Immediate intravenous lipid infusion in the successful resuscitation of ropivacaine-induced cardiac arrest after infraclavicular brachial plexus block. Reg Anesth Pain Med 2009;34:276-7.
108. Warren JA, Thoma RB, Georgescu A, Shah SJ. Intravenous lipid infusion in the successful resuscitation of local anesthetic-induced cardiovascular collapse after supraclavicular brachial plexus block. Anesth Analg 2008;106:1578-80, table of contents.
109. Weber F, Guha R, Weinberg G, Steinbach F, Gitman M. Prolonged Pulseless Electrical Activity Cardiac Arrest After Intranasal Injection of Lidocaine With Epinephrine: A Case Report. A A Pract 2019;12:438-40.
110. Whiteman DM, Kushins SI. Successful Resuscitation With Intralipid After Marcaine Overdose. Aesthet Surg J 2014;34:738-40.
111. Gnaho A, Eyrieux S, Gentili M. Cardiac arrest during an ultrasound-guided sciatic nerve block combined with nerve stimulation. Reg Anesth Pain Med 2009;34:278.
112. Markowitz S, Neal JM. Immediate lipid emulsion therapy in the successful treatment of bupivacaine systemic toxicity. Reg Anesth Pain Med 2009;34:276.
113. Smith NA. Possible side effects of Intralipid rescue therapy. Anaesthesia 2010;65:210-1.
114. Institute. NC. CTC V5. 0 and Common Terminology Criteria for Adverse Events v5. 0 (CTCAE). National Cancer Institute London; 2017.
115. Lin JW, Wang MJ, Yu HY, et al. Comparing the survival between extracorporeal rescue and conventional resuscitation in adult in-hospital cardiac arrests: propensity analysis of three-year data. Resuscitation 2010;81:796-803.
116. Avalli L, Maggioni E, Formica F, et al. Favourable survival of in-hospital compared to out-of-hospital refractory cardiac arrest patients treated with extracorporeal membrane oxygenation: an Italian tertiary care centre experience. Resuscitation 2012;83:579-83.
117. Nichol G, Leroux B, Wang H, et al; ROC Investigators. Trial of Continuous or Interrupted Chest Compressions during CPR. N Engl J Med 2015;373:2203-14.
118. Grasner JT, Lefering R, Koster RW, et al. EuReCa ONE-27 Nations, ONE Europe, ONE Registry: A prospective one month analysis of out-of-hospital cardiac arrest outcomes in 27 countries in Europe. Resuscitation 2016;105:188-95.
119. Andersen LW, Granfeldt A, Callaway CW, et al; American Heart Association’s Get With The Guidelines–Resuscitation Investigators. Association Between Tracheal Intubation During Adult In-Hospital Cardiac Arrest and Survival. JAMA 2017;317:494-506.
120. Soar J, Perkins GD, Maconochie I, et al; European Resuscitation Council. European Resuscitation Council Guidelines for Resuscitation: 2018 Update - Antiarrhythmic drugs for cardiac arrest. Resuscitation 2019;134:99-103.
121. Soar J, Donnino MW, Maconochie I, et al; ILCOR Collaborators. 2018 International Consensus on Cardiopulmonary Resuscitation and Emergency Cardiovascular Care Science With Treatment Recommendations Summary. Resuscitation 2018;133:194-206.
122. Goldschlager N, Epstein AE, Naccarelli GV, et al; Practice Guidelines Sub-committee, North American Society of Pacing and Electrophysiology (HRS). A practical guide for clinicians who treat patients with amiodarone: 2007. Heart Rhythm 2007;4:1250-1259. Erratum in: Heart Rhythm 2007;4:1590.
123. Thompson A, Balser JR. Perioperative cardiac arrhythmias. Br J Anaesth 2004;93:86-94.
124. Acute Respiratory Distress Syndrome Network, Brower RG, Matthay MA, Morris A, Schoenfeld D, Thompson BT, Wheeler A. Ventilation with lower tidal volumes as compared with traditional tidal volumes for acute lung injury and the acute respiratory distress syndrome. N Engl J Med 2000;342:1301-8.
125. Amato MB, Meade MO, Slutsky AS, et al. Driving pressure and survival in the acute respiratory distress syndrome. N Engl J Med 2015;372:747-55.
126. Pelosi P, Rocco PR. The lung and the brain: a dangerous cross-talk. Crit Care 2011;15:168.
127. Tremblay L, Valenza F, Ribeiro SP, Li J, Slutsky AS. Injurious ventilatory strategies increase cytokines and c-fos m-RNA expression in an isolated rat lung model. J Clin Invest 1997;99:944-52.
128. McKenzie N, Williams TA, Tohira H, Ho KM, Finn J. A systematic review and meta-analysis of the association between arterial carbon dioxide tension and outcomes after cardiac arrest. Resuscitation 2017;111:116-26.
129. Eastwood GM, Tanaka A, Bellomo R. Cerebral oxygenation in mechanically ventilated early cardiac arrest survivors: The impact of hypercapnia. Resuscitation 2016;102:11-6.
130. Eastwood GM, Schneider AG, Suzuki S, et al. Targeted therapeutic mild hypercapnia after cardiac arrest: A phase II multi-centre randomised controlled trial (the CCC trial). Resuscitation 2016;104:83-90.
131. Jakkula P, Reinikainen M, Hästbacka J, et al; COMACARE study group. Targeting two different levels of both arterial carbon dioxide and arterial oxygen after cardiac arrest and resuscitation: a randomised pilot trial. Intensive Care Med 2018;44:2112-21.
132. Tiruvoipati R, Pilcher D, Botha J, Buscher H, Simister R, Bailey M. Association of Hypercapnia and Hypercapnic Acidosis With Clinical Outcomes in Mechanically Ventilated Patients With Cerebral Injury. JAMA Neurol 2018;75:818-26.
133. Mekontso Dessap A, Charron C, Devaquet J, et al. Impact of acute hypercapnia and augmented positive end-expiratory pressure on right ventricle function in severe acute respiratory distress syndrome. Intensive Care Med 2009;35:1850-58.
134. Kilgannon JH, Jones AE, Shapiro NI, et al; Emergency Medicine Shock Research Network (EMShockNet) Investigators. Association between arterial hyperoxia following resuscitation from cardiac arrest and in-hospital mortality. JAMA 2010;303:2165-71.
135. Mckenzie N, Finn J, Dobb G, et al. Non-linear association between arterial oxygen tension and survival after out-of-hospital cardiac arrest: A multicentre observational study. Resuscitation 2021;158:130-8.
136. Bellomo R, Bailey M, Eastwood GM, et al; Study of Oxygen in Critical Care (SOCC) Group. Arterial hyperoxia and in-hospital mortality after resuscitation from cardiac arrest. Crit Care 2011;15:R90.
137. Pilcher J, Weatherall M, Shirtcliffe P, Bellomo R, Young P, Beasley R. The effect of hyperoxia following cardiac arrest - A systematic review and meta-analysis of animal trials. Resuscitation 2012;83:417-22.
138. Douzinas EE, Patsouris E, Kypriades EM, et al. Hypoxaemic reperfusion ameliorates the histopathological changes in the pig brain after a severe global cerebral ischaemic insult. Intensive Care Med 2001;27:905-10.
139. Becker LB. New concepts in reactive oxygen species and cardiovascular reperfusion physiology. Cardiovasc Res 2004;61:461-70.
140. Zwemer CF, Whitesall SE, D'Alecy LG. Cardiopulmonary-cerebral resuscitation with 100% oxygen exacerbates neurological dysfunction following nine minutes of normothermic cardiac arrest in dogs. Resuscitation 1994;27:159-70.
141. Vereczki V, Martin E, Rosenthal RE, Hof PR, Hoffman GE, Fiskum G. Normoxic resuscitation after cardiac arrest protects against hippocampal oxidative stress, metabolic dysfunction, and neuronal death. J Cereb Blood Flow Metab 2006;26:821-35.
142. Stub D, Smith K, Bernard S, et al; AVOID Investigators. Air Versus Oxygen in ST-Segment-Elevation Myocardial Infarction. Circulation 2015;131:2143-50.
143. Mickel HS, Vaishnav YN, Kempski O, von Lubitz D, Weiss JF, Feuerstein G. Breathing 100% oxygen after global brain ischemia in Mongolian Gerbils results in increased lipid peroxidation and increased mortality. Stroke 1987;18:426-30.
144. Liu Y, Rosenthal RE, Haywood Y, Miljkovic-Lolic M, Vanderhoek JY, Fiskum G. Normoxic ventilation after cardiac arrest reduces oxidation of brain lipids and improves neurological outcome. Stroke 1998;29:1679-86.
145. Hazelton JL, Balan I, Elmer GI, et al. Hyperoxic reperfusion after global cerebral ischemia promotes inflammation and long-term hippocampal neuronal death. J Neurotrauma 2010;27:753-62.
146. Danilov CA, Fiskum G. Hyperoxia promotes astrocyte cell death after oxygen and glucose deprivation. Glia 2008;56:801-8.
147. Hofmann R, James SK, Jernberg T, et al; DETO2X–SWEDEHEART Investigators. Oxygen Therapy in Suspected Acute Myocardial Infarction. N Engl J Med 2017;377:1240-9.
148. Nielsen N, Wetterslev J, Cronberg T, et al; TTM Trial Investigators. Targeted temperature management at 33°C versus 36°C after cardiac arrest. N Engl J Med 2013;369:2197-206.
149. Lascarrou JB, Merdji H, Le Gouge A, et al; CRICS-TRIGGERSEP Group. Targeted Temperature Management for Cardiac Arrest with Nonshockable Rhythm. N Engl J Med 2019;381:2327-37.
150. Bernard SA, Gray TW, Buist MD, et al. Treatment of comatose survivors of out-of-hospital cardiac arrest with induced hypothermia. N Engl J Med 2002;346:557-63.
151. Hypothermia after Cardiac Arrest Study Group. Mild therapeutic hypothermia to improve the neurologic outcome after cardiac arrest. N Engl J Med 2002;346:549-56.
152. Berg KM, Soar J, Andersen LW, et al; Adult Advanced Life Support Collaborators. Adult Advanced Life Support: 2020 International Consensus on Cardiopulmonary Resuscitation and Emergency Cardiovascular Care Science With Treatment Recommendations. Circulation 2020;142:S92-S139.
153. Mongardon N, Perbet S, Lemiale V, et al. Infectious complications in out-of-hospital cardiac arrest patients in the therapeutic hypothermia era. Crit Care Med 2011;39:1359-64.
154. François B, Cariou A, Clere-Jehl R, et al; CRICS-TRIGGERSEP Network and the ANTHARTIC Study Group. Prevention of Early Ventilator-Associated Pneumonia after Cardiac Arrest. N Engl J Med 2019;381:1831-42.
155. Constant AL, Mongardon N, Morelot Q, et al. Targeted temperature management after intraoperative cardiac arrest: a multicenter retrospective study. Intensive Care Med 2017;43:485-95.
156. Nolan JP, Soar J, Cariou A, et al. European Resuscitation Council and European Society of Intensive Care Medicine Guidelines for Post-resuscitation Care 2015: Section 5 of the European Resuscitation Council Guidelines for Resuscitation 2015. Resuscitation 2015;95:202-22.
